# Implementing a Cognitively Grounded Artificial Moral Advisor: A Multi-LLM Multi-Agent Approach Based on the Cognitive–Reflective Equilibration Model

**DOI:** 10.3390/jintelligence14090212

**Published:** 2026-09-02

**Authors:** Chulmin Kim, Seongjin Ahn

**Affiliations:** Department of Computer Education, Sungkyunkwan University, 25-2, Seonggyungwan-ro, Jongno-gu, Seoul 03063, Republic of Korea; sjahn@skku.edu

**Keywords:** ethical AI, artificial moral advisor, Cognitive–Reflective Equilibration Model, large language models, multi-agent systems, ethical decision-making, Cognitive–Reflective Equilibration Architecture

## Abstract

Large language model (LLM)-based artificial intelligence is increasingly used in ethically consequential human decision-making, yet fully autonomous machine ethics remains unrealistic, motivating architectures that support rather than replace human ethical judgment. This study introduces the Cognitive–Reflective Equilibration Architecture (CREA), a cognitively grounded artificial moral advisor that operationalizes the Cognitive–Reflective Equilibration Model (CREM), in which reflective reasoning guides ethical judgment from intuitive cognition toward a more advanced equilibrium among competing values, drawing on Piaget and Rawls. CREA implements CREM’s 20-step process through four stage-aligned reasoning agents—Cognitive, Reflective, Equilibration, and Evaluation—coordinated via multi-LLM orchestration, in which auxiliary models independently explore principles, generate counterarguments, and score supporting and opposing considerations to externalize reflective deliberation. The architecture was empirically evaluated by comparing four configurations—single-agent, multi-agent, multi-LLM, and multi-LLM with knowledge- and reasoning-bank augmentation—across four indicators of advice quality using 500 matched execution units per configuration. All comparisons are system-internal: advice quality was scored by CREA’s own multi-LLM measurement pipeline rather than by human ethicists, so the findings reflect relative differences among architectures under LLM-based self-evaluation, not normative validity. Within that scope, distributing reflective reasoning across multiple models was associated with higher reason-giving (justifiability) and normative-alignment scores relative to simpler configurations. CREA therefore offers an empirically characterized, auditable advisor architecture whose potential to scaffold human ethical judgment remains a hypothesis for user-centered validation rather than a demonstrated outcome.

## 1. Introduction

Human ethical decision-making is a higher-order cognitive activity situated at the intersection of cognitive science, moral psychology, and philosophy. When confronted with real-world moral dilemmas, human agents must render judgments under the constraints of bounded rationality and pervasive cognitive bias (Kahneman, 2011). These vulnerabilities do not disappear when moral reasoning is delegated to machines. Recent empirical work reports that the moral judgments produced by large language models can amplify, rather than attenuate, the cognitive biases observed in humans (Cheung et al., 2025), and the advice produced by such models presents two additional difficulties. The reasoning behind a model’s recommendation is largely opaque to the person receiving it, and the recommendation itself is unstable across executions: the same dilemma, posed twice, can yield different judgments supported by different reasons. Myers and Everett (2025), in a large-scale study of algorithmic aversion, show that transparency and justifiability in an advisor’s reasoning process are decisive conditions for securing human trust—precisely the properties that current model-generated advice lacks. As systems built on large language models are increasingly embedded in socially consequential domains—healthcare, public administration, education, and law—the question of whether such a system can systematically support, rather than supplant, human ethical reasoning has become a pressing scholarly problem.

Three concepts frame our answer. An artificial moral advisor is a system that supports a person’s ethical reasoning without making the decision for them; it is distinguished from an artificial moral agent, which acts on its own judgment and assumes the corresponding claim to moral authority. This study concerns the former. By “cognitively grounded,” we mean that the system’s procedure is derived from established accounts of how human reasoners revise moral judgments—here, Piaget’s theory of equilibration (Piaget, 1975/1985) and Rawls’s method of reflective equilibrium (Rawls, 2017)—rather than assembled from prompt-engineering heuristics. The claim is about the provenance of the procedure, not about any resemblance between the system’s processing and human cognition. The Cognitive–Reflective Equilibration Model (CREM), proposed by Kim and Ahn (2026), is the procedural model this study implements: a five-stage conceptual cycle and a 20-step executable sequence that takes a system from an intuitive judgment, through the recognition of conflict with relevant principles, to a revised judgment that the system can justify. Section 2.3 describes it in full; for present purposes, it can be understood as a specification of what a well-formed ethical deliberation must contain.

Existing artificial moral systems do not supply such a procedure. Most rely on rule-based inference or consequentialist calculation (Tolmeijer et al., 2020) and therefore fail to model the dynamic, context-sensitive character of human moral cognition (Giubilini & Savulescu, 2018; Landes et al., 2025). As Queloz (2025a) argues, moral knowledge cannot be exhaustively codified into a closed set of exception-free principles, so the appropriate goal is not the full automation of moral judgment but the provision of a structured, human-reviewable process of ethical deliberation. Systems grounded in a single ethical theory or a single language model face the further risks of confirmation bias and non-deterministic variability. We term this common deficit the cognitive grounding problem: the reasoning process of existing systems is misaligned with the structure of human moral cognition, making their advice difficult for humans to scrutinize, understand, and accept.

To address this problem, the present study operationalizes CREM as the Cognitive–Reflective Equilibration Architecture (CREA), a multi-agent, multi-LLM system that implements the four cognitive stages of CREM through six specialized agents—Orchestration, Cognitive, Reflective, Equilibration, Evaluation, and Measurement—coordinated as a LangGraph state machine. A five-model LLM ensemble realizes the plural weighing of perspectives called for by Rawls’s wide reflective equilibrium, while a three-tier Ethical Principles Knowledge Base (EPKB) and an Ethical Reasoning Bank (ERB) enable the accumulation, retrieval, and reuse of moral knowledge across executions through retrieval-augmented generation. To characterize what each architectural element contributes, the advice produced by CREA is evaluated across four configurations—Single-Agent, Multi-Agent, Multi-LLM, and Multi-LLM with knowledge- and reasoning-bank augmentation—across 500 executions per configuration.

The study is guided by three research questions:

RQ1. How should a multi-LLM, multi-agent architecture be designed in order to implement CREM as a functioning Artificial Moral Advisor?

RQ2. Can a CREM-based advisor deliver advice that is consistent, justifiable, procedurally valid, and normatively aligned—as captured by the metrics of Consistency, Justifiability, Procedural Validity, and the Normative Alignment Index (NAI)?

RQ3. How does the quality of the advice vary across the four architectural configurations?

This study makes three principal contributions. First, it systematically analyzes the cognitive grounding problem that has been underexamined in artificial moral advisor research and articulates design principles for an advisor rooted in a theory of human moral cognition. Second, it demonstrates a methodological pathway for operationalizing a theoretical ethical decision-making model as a multi-LLM multi-agent system. Third, it proposes a four-dimensional framework for evaluating advice quality and evaluates it through a large-scale comparative experiment spanning four architectural configurations and 500 executions per configuration.

The remainder of this paper is organized as follows. Section 2 reviews the literature on AMA typologies and the moral-advisor paradigm, the cognitive grounding problem, and LLM-based moral reasoning and computational reflective equilibrium, before introducing CREM. Section 3 presents the evaluation metrics, experimental design, and statistical analysis. Section 4 reports the results, Section 5 discusses their theoretical and practical implications together with the study’s limitations, and Section 6 concludes.

## 2. Theoretical Framework

### 2.1. Artificial Moral Advisors

#### 2.1.1. Conceptual Foundations and Typological Development of Artificial Moral Agents

Research on artificial moral agents has derived its intellectual foundations from fundamental questions about moral agency itself. Nagenborg (2007) pointed out that the ethical norms embedded in autonomous systems carry culturally specific presuppositions, arguing that any effective artificial moral agent framework must accommodate value pluralism across societies. Muntean and Howard (2014) expanded the conceptual landscape of artificial moral agents by proposing a model centered on three generative properties: creativity that goes beyond preprogrammed responses, autonomy as the independent exercise of ethical judgment, and sociality as the capacity to participate in moral discourse within social and multi-agent environments. Cervantes et al. (2020) systematically organized two decades of artificial moral agent scholarship, proposing a classification scheme based on the ethical theories implemented (utilitarian, deontological, and virtue-based), design strategies (top-down versus bottom-up), and levels of autonomy.

Formosa and Ryan (2021) identified three levels of moral adequacy for artificial moral agents: implicit ethical agents that prevent harm through constrained behavior, explicit ethical agents that apply ethical principles to novel cases, and full moral agents equipped with moral intuition and adaptive reasoning. Misselhorn (2022) provided a rigorous philosophical analysis of artificial moral agents in the context of responsible AI development, distinguishing functional moral agents—systems that act morally without bearing moral responsibility—from entities that could attain the status of full moral subjects, and argued that an artificial system can serve as a meaningful functional moral agent if it can recognize the morally relevant features of a situation and act accordingly. Arvan (2022) presented an influential trilemma concerning three possible artificial moral agent types: inhuman artificial moral agents that execute rules without understanding, better-human artificial moral agents that correct human moral biases, and human-like artificial moral agents that replicate human moral psychology. Extending this line of inquiry, Zafar (2025) examined the normative conditions under which AI systems could qualify as moral agents, and Firt (2025) analyzed what would make full artificial agents morally different from existing systems. Ghazali et al. (2025) surveyed the practical engineering challenges of artificial moral agent development, including the implementation of moral emotions, multi-agent ethical interaction, and the preservation of autonomy.

#### 2.1.2. From Autonomous Agents to Advisory Systems: The Artificial Moral Advisor Paradigm

A significant paradigm shift in AMA research emerged with the artificial moral advisor model, which redefines the role of AI from that of an autonomous decision-maker to that of an advisor providing cognitive support for human ethical deliberation. Giubilini and Savulescu (2018) proposed the foundational model of the artificial moral advisor as a quasi-relativist ideal observer implementing Firth’s classical ideal-observer theory, and argued that such a system should assist humans in attaining both narrow and wide reflective equilibrium. Their advisor is characterized by disinterestedness, dispassion, and consistency, yet it remains non-absolutist in that it is calibrated to the value system of the individual user.

With respect to AMA design and development, Behdadi and Munthe (2020) proposed a methodological reorientation away from purely metaphysical debates over whether AI can qualify as a moral agent and toward a practical framework that specifies the properties AI systems must possess in order to function effectively and responsibly in moral contexts; the design criteria prescribed by this approach are sensitivity to normative reasons, procedural transparency, and contextual adaptability. Tassella et al. (2023) extended the advisor concept to the context of moral-dilemma resolution, proposing three functional roles for AI moral advisors: identifying dilemmas by classifying ethical challenges, presenting dilemmas by structuring the normative landscape for deliberation, and resolving dilemmas by providing reasoned recommendations.

Critical objections to artificial moral advisors continue to be raised. Myers and Everett (2025) demonstrated that people trust AI moral advisors less than human advisors, and that this distrust is especially pronounced for advice grounded in utilitarian principles. Queloz (2025b) identified fundamental limitations of both personalized and generalist AI moral advisors, arguing that the non-systematic character of the normative domain—the impossibility of fully codifying moral knowledge into exceptionless general principles—imposes principled limits on what any AI moral advisor can achieve. Liu et al. (2022) contended that most AMA designs wrongly presuppose a static framework of moral values and fail to account for the dynamic, context-sensitive character of human moral psychology; their discussion implies that artificial moral advisors must accommodate not only static ethical principles but also the context dependence and responsiveness of human moral psychology.

Taken together, these critiques converge on a structural point: the non-systematic character of the normative domain and the context sensitivity of moral judgment pose difficulties that a single model operating from a single perspective is poorly positioned to resolve. They thus motivate an architectural response—one in which multiple independent perspectives are generated, confronted, and coordinated within an explicit procedural structure—which the next section develops through the theory of multi-agent systems.

### 2.2. Multi-Agent Systems

Intelligent agents are artificial entities that perceive their environment, reason about it, and act to achieve specific goals. These agents range from single autonomous entities to highly cooperative multi-agent systems (MAS; Piccialli et al., 2025; Xi et al., 2025). Single-agent systems concentrate computational resources and decision-making in one entity, offering design simplicity and rapid task responsiveness, but they exhibit limited scalability and adaptability in complex, dynamic problem spaces (Li et al., 2024).

Multi-agent systems overcome these limitations by distributing decision-making and problem-solving across multiple agents. Each agent independently perceives and judges its environment while retaining partial autonomy and interacts with other agents by emulating real-world coordination patterns such as cooperation, competition, and hierarchical organization (Li et al., 2024). This distributed paradigm enables specialization and emergent collective behavior unattainable by a single agent, and at the architectural level it is shaped by two dimensions: agent composition (homogeneous vs. heterogeneous) and communication structure (decentralized, centralized, hierarchical, or nested; Li et al., 2024).

The advent of large language models (LLMs) has reconfigured this paradigm. LLMs have demonstrated substantial capabilities in reasoning and planning that align with the functional requirements of agents that must perceive and act within an environment (Li et al., 2024; Xi et al., 2025). What separates an LLM used as an agent from an LLM used as a static text generator is architectural rather than parametric. Surveys of LLM-based autonomous agents converge on a unified construction framework comprising a profile module that defines the agent’s role, a memory module that retains information across steps, a planning module that decomposes goals into executable actions, and an action module that executes them within an environment or through a set of tools, together with mechanisms by which the agent acquires or refines capability over time (Wang et al., 2024). Xi et al. (2025) organize the same territory as a perception–decision–action framework and extend it to societies of LLM-based agents in which multiple agents cooperate, compete, or negotiate within a shared environment. Agenthood on this account is a matter of degree rather than a binary property: the more of these modules a system instantiates, and the more of its behavior is governed by them rather than by a single prompt, the greater the degree of agency-related functionality it exhibits, and multi-agent configurations sit at the upper end of that progression (Wang et al., 2024; Xi et al., 2025).

Orchestration architecture. Building an orchestrated MAS goes beyond simply connecting multiple autonomous agents; it requires a coordination layer that governs their interaction. Zhu et al. (2026) define orchestration as five interrelated mechanisms—task decomposition and allocation, inter-agent communication and context sharing, state management and persistence, control-flow sequencing, and error detection and recovery—and show that contemporary frameworks differ chiefly in how strongly each mechanism is guaranteed; graph-based frameworks such as LangGraph, for instance, provide explicit state checkpointing with configurable persistence backends. Complementing this coordination view, a systematic review of foundation-model-based agent designs by Liu et al. (2025) catalogs eighteen architectural patterns from which such systems are assembled. Among them, role-based cooperation differentiates agents by assigned role; retrieval-augmented generation grounds an agent in external knowledge rather than parametric recall; cross-reflection involves one model critiquing the output of another; multimodal guardrails constrain what an agent may emit; and an agent evaluator provides a dedicated component that assesses the output of the task-performing agents. These patterns supply the quality-assurance and oversight functions that an orchestrated system requires in addition to the agents that perform its tasks.

Interaction structures and communication protocols. Agent communication structures can be classified into four types: hierarchical, in which superordinate agents assume responsibility for supervision and decision-making; decentralized, in which agents communicate directly peer-to-peer without central authority; centralized, in which a central agent coordinates the whole; and shared message pools, in which agents exchange information in a publish–subscribe manner (Li et al., 2024). Zhu et al. (2026) observe the same core topologies in production frameworks and add an orthogonal dimension of adaptivity—dynamic routing, changing membership, and learned coordination—that may be layered onto any base topology. At the protocol level they distinguish two complementary layers: the Model Context Protocol (MCP), which standardizes an agent’s access to external tools and data, and Agent-to-Agent (A2A) protocols, which govern peer-level discovery, coordination, and delegation through advertised capability descriptions rather than through a central registry.

Functional workflow. From a workflow perspective, the LLM-based MAS life cycle can be described in terms of five core modules: profile (instantiating agent roles), perception (interpreting environmental information and retrieving knowledge), self-action (memory storage and planning), interaction (message passing and cooperative or competitive relations), and evolution (feedback-based self-reflection; Li et al., 2024)—a decomposition that corresponds closely to the profile, memory, planning, and action modules derived independently by Wang et al. (2024). In combination, these modules are argued to support distributed decision-making, collective intelligence, and the scalability, robustness, and flexibility that a single agent cannot achieve (Piccialli et al., 2025; Li et al., 2024). We note, however, that the evidence for these benefits comes largely from task-performance benchmarks and framework demonstrations; independent replications of deployment-scale claims remain scarce (Zhu et al., 2026).

Among the concepts and components of multi-agent systems reviewed above, the elements subsequently employed in the present study are as follows:

- a role-differentiated multi-agent structure, in which agents are specialized by assigned reasoning role (role-based cooperation; Liu et al., 2025);
- a multi-LLM configuration assigning multiple heterogeneous LLMs to distinct reasoning roles, so that alternatives and counterarguments are generated independently rather than by a single model (cross-reflection and debate-based cooperation; Liu et al., 2025);
- a procedural control structure implementing the orchestration mechanisms of task decomposition and allocation, control-flow sequencing, state management and persistence, and error detection and recovery, realized as an explicit state graph (Zhu et al., 2026);
- a knowledge-base and reasoning-bank design for retrieval-based grounding (retrieval-augmented generation; Liu et al., 2025);
- communication-interface design principles that standardize agent-to-tool and agent-to-agent information exchange (the MCP and A2A concepts; Zhu et al., 2026);
- an execution coordination scheme based on hierarchical and centralized interaction structures (Li et al., 2024; Zhu et al., 2026);
- an agent procedure design reflecting the five functional modules of profile, perception, self-action, interaction, and evolution (Li et al., 2024; Wang et al., 2024);
- a dedicated evaluation component separate from the task-performing agents (agent evaluator; Liu et al., 2025), realized in CREA as the Measurement Agent.

### 2.3. The Cognitive–Reflective Equilibration Model (CREM)

The proliferation of large language models has sharpened a familiar difficulty in AI ethics. Declarative principles—fairness, transparency, explainability, accountability, privacy, safety—supply normative direction but underdetermine action when principles collide, as they routinely do in the fairness–privacy trade-off, and they offer no procedure for resolving such collisions consistently. For systems whose outputs are probabilistically generated and non-deterministic, the difficulty compounds: the same dilemma may elicit different judgments on different runs, and the reasoning that produced either judgment is not recoverable from the output alone. The Cognitive–Reflective Equilibration Model (CREM) responds by shifting the question from which principles a system should follow to how a system should proceed in order to arrive at judgments that are consistent, inspectable, and justifiable (Kim & Ahn, 2026).

CREM rests on an explicit ontological premise that also delimits the claims of the present paper. It does not assert that a language model performs moral judgment in the philosophical sense, which would presuppose free will, conscience, and intentionality. “Ethical decision-making” here denotes the procedural execution of structured ethical reasoning steps—functional ethical processing that yields procedurally consistent, contextually appropriate, and normatively informed output. On this construal, a CREM-based system is an ethics procedure executor, not a moral agent, and its outputs acquire normative standing only through the human deliberation they inform. This premise is why CREA is developed as an artificial moral advisor rather than an autonomous artificial moral agent.

#### 2.3.1. Theoretical Foundations: Integrating Piaget and Rawls

CREM integrates two traditions that describe how a reasoner moves from an unstable state to a better-justified one.

Piaget (1975/1985) characterizes equilibration as a dynamic balance internal to intellectual functioning. A cognitive system handles environmental events with its existing structures (assimilation) and modifies those structures when the environment resists them (accommodation); development is a continual alternation of equilibrium and disequilibrium, and equilibration is a constructive process always oriented toward a more advanced equilibrium. Procedurally, the cycle runs from an initial equilibrium, through contact with the environment and assimilation, into disequilibrium, and via accommodation to a better equilibrium. Crucially for our purposes, accommodation operates through reflective abstraction—differentiation and integration, relativization of concepts, and quantification of relations—which functions as a logico-mathematical reasoning process rather than as mere adjustment.

Rawls (2017) developed reflective equilibrium as a coherentist method of justification in which considered judgments and principles are brought into mutual support through iterative adjustment. Responding to charges of intuitionism and relativism, he distinguished narrow reflective equilibrium, which establishes mutual support between considered judgments and principles, from wide reflective equilibrium, which additionally weighs alternative conceptions of justice together with the comparative strength of the reasons for and against them. Daniels (1979) elaborated wide reflective equilibrium as the philosophically substantive form of the method, and it is this wide form—comparative weighing of competing conceptions and their supporting reasons—that CREM operationalizes.

The integration rests on a structural equivalence between the two procedures rather than on a loose analogy. Both are constructivist rather than foundationalist; both proceed by building coherence; both advance through cycles rather than in a single pass; and both locate the decisive work in a reflective mechanism that reorganizes the reasoner’s commitments. The specific hinge is that the justification of principles in wide reflective equilibrium—assessing the relative weight of supporting and opposing reasons—can be read as Piagetian reflective abstraction applied to normative content. Following Daniels, Rawls’s procedure is first stripped of its justice-theoretic specificity so that it applies to ethical judgment generally; Piaget’s six developmental stages are then refined to five procedural stages; and the two are aligned.

#### 2.3.2. The Humanities-Based Meta-Model of Ethical Decision-Making

A theory of equilibration does not by itself specify what a reasoner should do at each stage. To supply that content, Kim and Ahn (2026) examined four established applied-ethics decision models from different disciplinary traditions—Rest’s four-component model, Loewenberg and Dolgoff’s social-work practice model, Beauchamp and Childress’s biomedical principlism, and Cooper’s public-administration model—and found that they share a strikingly consistent procedural skeleton. Synthesizing this skeleton with six humanistic traditions yielded a six-stage meta-model: problem recognition (phenomenology: epoché and eidetic intuition), analysis of objective information and context (hermeneutics: fusion of horizons), exploration of alternatives (dialectics: sublation and dramatic rehearsal), ethical judgment (Kantian practical reason: the categorical imperative, universalizability, prima facie duties), implementation (existentialism: responsible choice under radical freedom), and reflection and evaluation (Habermasian discourse ethics: communicative and public justification). This meta-model mediates the transition from theory to operation: it specifies the function that each stage of the equilibration cycle must perform.

#### 2.3.3. The Conceptual Model (CCREM)

CREM comprises a conceptual model and an operational model (CREM = CCREM + OCREM). The conceptual model specifies a five-stage cycle in which each stage has a determinate correspondence in both source theories (Table 1).

Two features of this cycle matter for the architecture developed in the present paper. First, disequilibrium is not a failure mode but the engine of the procedure: a system that never registers conflict never accommodates. Second, accommodation is the stage at which plurality does its work, because comparing alternative principles and weighing competing reasons is precisely what wide reflective equilibrium requires. These two features motivate, respectively, CREA’s explicit conflict-detection step and its use of multiple independent models at the accommodation stage.

#### 2.3.4. The Operational Model (OCREM): Four Stages, Twenty Steps

OCREM converts the conceptual cycle into an executable procedure. Each CCREM stage is restated as an ethical-judgment process, mapped onto the procedural elements of the humanities-based meta-model, and paired with functionally appropriate LLM reasoning techniques—zero-shot chain-of-thought and ReAct for information analysis, self-consistency and recursive criticism and improvement for consistency adjudication, Tree of Thoughts and Reflexion for alternative exploration and self-correction, and moral self-correction and constitutional constraints for value alignment. The initial-equilibrium stage is a theoretical precondition rather than an executable operation and is therefore omitted from the operational model, leaving four stages: cognitive processing (steps 1–6), reflective processing (steps 7–15), equilibration and ethical judgment (steps 16–17), and ethical implementation and evaluation (steps 18–20). The resulting twenty prompt requests were refined through iterative prototyping and can be executed on any compatible model in sequential query–response form, without fine-tuning or architectural modification (Table 2 and Table A1).

The procedure is deliberately branched rather than strictly linear, and the branches are theoretically motivated by the equilibration cycle itself. At step 5, if the intuitive judgment and the retrieved principles are found to be in complete mutual support—that is, if no disequilibrium arises—the system writes its final decision statement and skips directly to step 18, because there is nothing to accommodate. At steps 8 to 10, if neither selection nor mutual adjustment is possible, the procedure terminates with an explanation rather than forcing a resolution. At step 16, if one candidate judgment’s supporting weight exceeds its opposing weight by a preset margin, that judgment is adopted and step 17 is skipped. Consequently, a valid execution may comprise nine, seventeen, eighteen, or twenty steps, and the number of executed steps is a property of the dilemma and of the reasoning, not an index of procedural failure. This point becomes important when procedural validity is measured, and we return to it in Section 3 and Section 5.

#### 2.3.5. Prior Validation of OCREM

OCREM was validated in a proof-of-concept study before the architecture reported here was built (Kim & Ahn, 2026), and its design is summarized here because it clarifies what is already known and what remains unresolved. Five contemporaneous models (ChatGPT-4o, Claude Sonnet 3.7, Gemini 1.5 Pro, LLaMA 3 70B Instruct, and DeepSeek-V3) executed the twenty-step procedure on a bilingual Korean–English set of twenty ethical dilemmas—ten from human social contexts and ten from AI technology contexts—producing 200 executions and 4000 request–response pairs. The same five models then rated the procedural validity of each step on a five-point scale, yielding 528 analyzable evaluation cases and 10,560 step-level measurements. Because models of the same families both generated and scored the output, the authors added a supplementary human evaluation in which five experts from practical ethics and pastoral care, clinical psychology, medicine, AI and computer engineering, and law independently assessed twenty-five executions using an instrument that included an item on the validity of the final ethical judgment that the model-based instrument did not measure.

Model-based ratings yielded a mean procedural validity of 4.25 (SD = 1.03) with internal consistency of α = 0.898 and inter-rater agreement of ICC(A,k) = 0.716 and ICC(C,k) = 0.822. Human experts were directionally consistent but systematically more conservative, at M = 3.85 (SD = 1.12), α = 0.858, and ICC(C,k) = 0.678, and their ratings of the final ethical judgment significantly exceeded the scale midpoint (M = 3.85, t(24) = 8.00, *p* < .001, d = 0.79), with 68% of ratings at four or above. A branch-aware analysis of 1000 evaluation cases found that 66% completed all twenty steps, 25% took a partial re-equilibration path of sixteen to nineteen steps, and 8.5% reached early equilibrium at step 6; path type was unrelated to the executing model or the dilemma domain, and scores on the mandatory steps did not differ between complete and branched executions (M = 4.62 vs. 4.63), confirming that early equilibrium is a theoretically expected branch rather than a procedural failure.

The proof-of-concept study also delineated the limits of its conclusions. It included no control or baseline condition—no direct answering, generic chain-of-thought, or constitutional-AI comparison—so it demonstrated feasibility and expert-rated acceptability rather than the causal contribution of the procedure; its scope was confined to five models and twenty predominantly Western-derived dilemmas; prompt-wording sensitivity was not systematically tested; and the expert panel was small, purposively sampled, and uncalibrated. The authors accordingly identified two directions for future work: comparing CREM against baselines under blinded expert rating and embedding CREM in a multi-agent architecture in which role-specialized agents perform iterative deliberation and mutual criticism, supported by retrieval-augmented grounding in curated principles and by persistent memory for reusing prior equilibration outcomes.

#### 2.3.6. From CREM to CREA: The Gap Addressed Here

The present study takes up the second of those lines. CREM specifies what a well-formed ethical reasoning procedure looks like and shows that a single model can execute it; it does not tell us how such a procedure should be distributed across a system, nor whether distributing it helps. That question is not merely an engineering one. Wide reflective equilibrium requires the weighing of alternative conceptions and of the reasons for and against them, and a single model weighing its own alternatives is, in this sense, a single perspective simulating plurality. Whether externalizing that plurality across independent models yields advice that is more consistent, better justified, and more normatively aligned—and what such an arrangement costs in procedural fidelity—is an empirical question about architecture that CREM leaves open.

CREA therefore implements OCREM as a multi-agent, multi-LLM architecture and compares four configurations along the path from single-agent execution to a knowledge- and reasoning-bank-augmented multi-LLM system. The design decisions that constitute this architecture follow directly from the theory outlined above; Table 3 states these correspondences in full, tracing each theoretical commitment to the architectural decision it licenses and to the point in the system where that decision is implemented. CREM therefore serves not merely as background but as a specification for the present study: it fixes the procedure, the stage boundaries, and the point at which plurality is theoretically warranted, and the present study tests what happens when that specification is realized architecturally.

### 2.4. Recent Research on Ethical AI

This section reviews prior research directly related to CREA—an ethical multi-agent architecture that extends a coherence-driven model of reflective equilibrium—organized around two themes: ethical decision-making in AI and ethical multi-agent systems, with a focus on how each line of work resembles the present study and how it differs from it.

#### 2.4.1. Ethical Decision-Making in AI

Early research sought to explain moral judgment by computationally emulating human cognitive processes. The LIDA-based model of Wallach et al. (2010) shares the present study’s orientation in that it integrates emotion and reason to perform moral judgment, but as a single-agent cognitive model it lacks a deliberative process in which conflicting ethical perspectives are distributed and coordinated. Most notably, Yilmaz et al. (2017) formalized reflective equilibrium and multi-coherence theory as a constraint-satisfaction problem, making their work theoretically the closest to CREM; it nevertheless differs from the present study in that it relied on a single reasoning engine and was not extended to multi-agent collaboration.

The logic- and probability-based approaches of Conitzer et al. (2017) and Machado et al. (2024) are similar to the present study in that they pursue general, formal ethical reasoning beyond ad hoc rules, but they presuppose a single unified framework and do not address coordination among agents representing different perspectives. The deep-learning approach of Wiedeman et al. (2020), meanwhile, demonstrates data-driven learning of group morality, but it diverges from the present study with respect to the coherence and explainability of the grounds for judgment.

Research on artificial moral advisors (Tassella et al., 2023; Fabre et al., 2024) focuses on the role of AI in assisting and enhancing human ethical judgment; because it emphasizes the presentation of arguments rather than the derivation of conclusions, it contrasts with the present study’s orientation toward an internally autonomous, coherence-driven deliberation that derives a reasoned conclusion, which is then delivered as advice rather than enacted as an autonomous decision. Principle-, framework-, and domain-specific studies (Ferrell et al., 2024; Osasona et al., 2024; Senghor et al., 2025; Putica et al., 2025) provide procedural frameworks emphasizing organizational and social contexts and stakeholder participation, but they place their emphasis on governance and procedural design rather than on computational mechanisms for coherently reconciling conflicting principles.

#### 2.4.2. Ethical Multi-Agent Systems

Several studies share the recognition that ethics is inherently a multi-agent problem. Robbins and Wallace (2007), the ETHICAA team (Belloni et al., 2015), Murukannaiah et al. (2020), and Gal and Grosz (2022) address ethical conflicts and sociotechnical interactions in open, decentralized environments, and in this respect they align with the premises of the present study; for the most part, however, they stop at presenting conceptual foundations or role definitions and do not provide a concrete deliberative mechanism that brings conflicting principles into convergence on the basis of coherence.

Research on norm emergence and fairness (Mashayekhi et al., 2022) partially shares the present study’s goals in that it designs systems so that desirable norms and fairness emerge at the system level. These studies, however, rely on emergent interaction or fairness constraints, which differs from reconciling plural ethical principles through reflective equilibrium to arrive at a single coherent conclusion. Stenseke (2024) and Woodgate (2025), who operationalized norm-based and virtue ethics, likewise concentrate on the computational implementation of particular ethical theories and thus differ from the present study, which focuses on the coherent adjustment of multiple principles.

Domain-application studies by Stomberg and Tröschel (2024) and Chen et al. (2025) demonstrated the practical feasibility of combining ethical judgment with multiple agents in energy negotiation and clinical decision-making, respectively, but they rely on pre-agreed rules or governance and thus differ from autonomous coherence-based deliberation. Recent LLM-based multi-agent studies (Yamani et al., 2025) are formally the most similar to the present architecture in their role differentiation and structured debate. However, lacking an explicit theoretical foundation in coherence or reflective equilibrium they cannot systematically guarantee the consistency and explainability of judgments, which fundamentally distinguishes them from CREA.

#### 2.4.3. Synthesis: Positioning the Present Study

In sum, the two streams—coherence-driven reflective equilibrium (Yilmaz et al., 2017) and multi-agent-level scalability (together with the LLM-based multi-agent studies reviewed above)—have each developed independently, but architectures that combine these two streams remain rare.

Stated at a finer grain, this combined gap resolves into three distinct gaps. First, procedural accounts of machine ethics specify what a well-formed deliberation should look like, but they are demonstrated on a single model, so what happens when the procedure is distributed across a system is unknown—the question already raised from within the theory in Section 2.3.6. Second, work on multi-agent LLM deliberation reports gains in task performance but rarely holds the reasoning procedure constant across conditions, so architectural effects are confounded with differences in what the agents were asked to do. Third, evaluations of LLM moral reasoning overwhelmingly score final answers rather than the path that produced them, leaving procedural fidelity unmeasured even where the procedure is the contribution. The present study addresses all three: it holds a fully specified 20-step procedure constant, varies only the architecture that executes it, and scores the reasoning path as well as the outcome.

CREA accordingly differs from the systems reviewed above in three respects, each addressing one of these gaps. Its procedure is derived from two established accounts of how reasoners revise moral judgments—Piagetian equilibration and Rawlsian wide reflective equilibrium—rather than assembled from prompt-engineering heuristics, so each step carries a stated theoretical warrant. Its use of multiple models is placed where the theory locates the key deliberative work—at the accommodation stage, where alternative principles and competing reasons are weighed—rather than applied uniformly as an ensembling technique. And its evaluation targets four dimensions of the reasoning process—consistency, Justifiability, procedural validity, and normative alignment—rather than the acceptability of the final recommendation alone. We note at the outset the corresponding limitation: the primary evaluation is performed by the same families of models that generate the advice, so it establishes relative differences among architectures under model-based assessment; a supplementary human expert evaluation addresses this in part, and what neither establishes is the normative validity of any output.

Attending to these gaps, the present study extends CREM into a multi-agent architecture (CREA) in which agents representing different ethical perspectives converge, through interaction, on coherent and explainable conclusions. Table 4 summarizes the similarities and differences between representative prior studies and the present study.

## 3. Methods

This study developed and evaluated CREA (Cognitive–Reflective Equilibration Architecture), a multi-agent system that operationalizes the 20-step Cognitive–Reflective Equilibration Model (CREM) of ethical decision-making. Four architectural configurations of CREA were implemented and compared under identical conditions to isolate the contribution of agent specialization, multi-LLM deliberation, and externalized normative knowledge to the quality of automated ethical reasoning.

### 3.1. System Implementation and Infrastructure

CREA was implemented as a working software system in which a user submits a moral dilemma through a web interface and receives, step by step, the system’s reasoning and final advice. Internally, a workflow engine executes the 20-step CREM procedure according to its specified control flow, calling on five commercial large language models from different providers (OpenAI GPT-4.1, Anthropic Claude Haiku 4.5, Google Gemini 2.5 Flash, xAI Grok-3, DeepSeek V3). To make repeated runs as comparable as possible—a prerequisite for the statistical comparisons reported below—all models were configured to respond as deterministically as their settings allow, and every execution was saved as a complete execution record designed to preserve integrity. Software components, model versions, and infrastructure safeguards are detailed in Appendix B.1.

### 3.2. Agent Architecture and CREM Workflow

The 20-step procedure is divided among six agents, each responsible for one phase of deliberation, in direct correspondence with the stages of CREM. The Cognitive agent (Steps 1–6) forms an initial intuitive judgment about the dilemma and checks whether that judgment conflicts with relevant ethical principles. If a conflict is found, the Reflective agent (Steps 7–15) explores alternatives and scores the considerations for and against each candidate judgment. The Equilibration agent (Steps 16–17) then determines whether one candidate is sufficiently well supported to be adopted; if not, deliberation continues with a further round of mutual adjustment. The Evaluation agent (Steps 18–20) reviews the resulting judgment for consistency, justifiability, and predicted outcomes. Separately from this reasoning pipeline, a Measurement agent scores the quality of the completed advice post hoc, and an Orchestration agent manages the order of execution and the three decision points at which the workflow branches (after Steps 5, 8, and 16). The exact branching rules and state-management mechanics are given in Appendix B.2.

### 3.3. Knowledge Components: EPKB and ERB

Two reference components ground the system’s reasoning in ethical knowledge. The Ethical Principles Knowledge Base (EPKB) is a library of ethical principles—fourteen built-in traditions such as utilitarianism, deontology, virtue ethics, and care ethics, supplemented by material from scholarly encyclopedias of philosophy and from user-supplied documents. The Ethical Reasoning Bank (ERB) is an archive of the system’s own completed deliberations: each finished execution is stored in full, and when a new dilemma arrives, the most similar past cases that met a quality threshold can be retrieved for reference (Dataset S1). At designated steps, the system searches these components for passages relevant to the dilemma at hand and incorporates the retrieved passages into the reasoning context. Retrieval parameters, similarity formulas, and chunking details are given in Appendix B.3.

Despite this machinery, both components function as reference resources rather than learning mechanisms: no model is retrained and nothing about the system’s norms changes at run time. Retrieved material appeared in roughly one step per execution for each component. We therefore treat the components as externalized, reusable references rather than as autonomous moral learning.

The two components also differ in evaluation status. The EPKB was fixed and identical wherever it was available. The ERB grows during use: every execution was written to the bank on completion, but reading from the bank was enabled only in the Multi-LLM + KRB condition, so comparisons among the other three conditions are unaffected by its contents. ERB retrieval does not exclude the dilemma currently being solved—a deliberate design choice, since CREM treats accommodation as building on accumulated equilibration outcomes, much as case-based moral reasoning relies on on-point precedent. Consequently, executions of the same dilemma in the augmented condition are more strongly interdependent than in the other conditions, and that condition’s gain over Multi-LLM conflates cross-dilemma transfer with same-dilemma self-reference; both points are discussed as limitations in Section 5.4.

### 3.4. Multi-LLM Ensemble and Outcome Measures

The five language models served two distinct roles, and the distinction matters for interpreting the results. First, during reasoning, the Multi-LLM and Multi-LLM + KRB conditions consulted all five models at five designated steps: one primary model drafted a response, the other models independently produced their own, and the primary model synthesized them into a single analysis attributing each perspective to its source. This is the mechanism by which the architecture externalizes the plural weighing of perspectives. Second, after reasoning was complete, the Measurement agent always asked all five models to score the finished advice—in every condition, including Single-Agent. This post hoc scoring produced the four quality indicators: Consistency and Justifiability (5-point ratings), Procedural Validity (each of the 20 steps scored individually and aggregated), and the Normative Alignment Index (NAI), a composite reflecting how relevant the advice is to each ethical principle and how well it aligns with that principle. An overall mean across the four indicators was also computed, and the internal consistency of the five models’ scores was estimated with Cronbach’s α in each condition. Scoring formulas and aggregation details are given in Appendix B.4 and Appendix C.

### 3.5. Experimental Conditions

The design elements above define four conditions that differ in how the reasoning is organized, not in how it is scored. (1) Single-Agent executes the 20 steps as a single undivided procedure executed by a single model. (2) Multi-Agent divides the same procedure among the six specialized agents, still with a single model. (3) Multi-LLM adds the five-model deliberation at the designated steps. (4) Multi-LLM + KRB additionally grants access to the EPKB and the ERB. Crucially, the post hoc scoring was identical across all four conditions: the same five-model panel was used to evaluate every execution. Table 5 summarizes the differences.

### 3.6. Data Preparation and Statistical Analysis

Four architectural configurations of CREA—Single-Agent, Multi-Agent, Multi-LLM, and Multi-LLM + KRB—were compared on four core evaluation indicators (Consistency, Justifiability, Procedural Validity, and the Normative Alignment Index [NAI]) and on the equally weighted mean of these indicators. The evaluation dataset was generated from a balanced factorial design crossing the four architectural conditions with 20 moral dilemma cases, five executor LLMs, and five execution rounds. Each condition therefore comprised 500 execution units (20 × 5 × 5), and every execution unit was uniquely indexed by its dilemma case, executor LLM, and round. Execution units were matched across conditions on these three indices, yielding 500 matched pairs for each of the six pairwise architecture comparisons. The 20 dilemmas—ten from human social contexts and ten from AI technology contexts (Table A2)—were fixed in advance and reused across conditions so that the matching would be exact. All architectures were executed under a common deterministic LLM configuration (temperature = 0.0, max_tokens = 3072, extended-thinking and reasoning tokens disabled); five rounds per dilemma and executor were nonetheless run to average over the residual non-determinism at that setting. Repeated executions are therefore not independent: 25 executions share each dilemma within a condition, and we address that dependence with the mixed-effects models described below rather than by treating the units as exchangeable.

The study was designed as an exploratory comparative evaluation: no directional hypotheses were preregistered, and no analysis plan was registered before data collection. The six contrasts comprise the three adjacent transitions that follow the incremental construction of the architecture (Single-Agent to Multi-Agent, Multi-Agent to Multi-LLM, Multi-LLM to Multi-LLM + KRB) and the three cumulative contrasts against the Single-Agent and Multi-Agent baselines. The adjacent transitions isolate the effect of adding one component at a time; the cumulative contrasts capture the net effect of the full development path. All six contrasts are reported for every indicator, so no contrast was selected after inspecting the results.

Architecture differences were examined in two stages. Friedman omnibus tests across the four matched conditions were computed first for each indicator and for the overall mean. Pairwise differences were then evaluated with paired-samples *t*-tests across the 500 matched execution units (df = 499), reported with 95% confidence intervals and Cohen’s dz effect sizes for matched samples, and complemented by Wilcoxon signed-rank tests as a distribution-free check. Holm correction was applied within each indicator across all six contrasts to control the family-wise Type I error rate. Effect sizes were interpreted using conventional benchmarks for dz (approximately 0.2, 0.5, and 0.8 for small, medium, and large), and statistical significance is reported separately from practical importance throughout. Cross-family procedural-validity contrasts are additionally qualified by the measurement asymmetry, which is taken up in Section 5.2.3.

Internal consistency was assessed with Cronbach’s α, treating the five LLM-derived ratings of one execution unit as items. So construed, α indexes the internal consistency of the measurement scores in a form equivalent to a standardized agreement index related to ICC(2,k); it is not conventional inter-rater reliability, evidence of normative correctness, or evidence of freedom from biases shared across models trained on overlapping corpora (Zheng et al., 2023). Alpha was computed at the metric-specific level (the five-item scale underlying each indicator), for the 15-item overall measurement scale, and for the execution-level four- and three-indicator composite scales; the composite coefficients pool conceptually heterogeneous dimensions and are reported as summaries of measurement coherence rather than as evidence of a single underlying construct. Differences in α between successive architectures were tested with paired bootstrap 95% confidence intervals and paired permutation tests (3000 resamples each). These analyses are secondary and exploratory: they characterize the behavior of the measurement instrument rather than the architectures, and they were not planned before data collection.

Finally, score distributions were characterized with extended descriptive statistics (mean, standard deviation, variance, coefficient of variation, minimum, median, and maximum) and box-plot visualizations (median, interquartile range, whiskers, and outliers) for each architecture and indicator.

All statistical analyses were performed in Python 3.11.15 running in a Linux container environment; the analysis script requires Python 3.10 or later. Data handling was performed with pandas 3.0.2 and NumPy 2.4.4. Paired-samples t tests and their 95% confidence intervals were derived from the t distribution, and Wilcoxon signed-rank tests and Friedman omnibus tests were computed with SciPy 1.17.1; Holm correction within each indicator was implemented as a custom NumPy routine. Cronbach’s α was computed using the standardized formula implemented with NumPy, and the paired bootstrap confidence intervals and paired permutation tests for α differences were implemented as custom NumPy routines with a seeded pseudo-random generator (numpy.random.default_rng, seed = 20260805; 3000 resamples each). Linear mixed-effects models were fitted with statsmodels 0.14.6 (MixedLM, restricted maximum likelihood) with dilemma case as a random intercept. Figures were produced with matplotlib 3.10.9 and the result workbooks with openpyxl 3.1.5. The analysis code—which reconstructs execution-level scores from the evaluator-level raw data and regenerates every reported table and figure in a single run—and the parser used to recover model calls, tokens, latency, and cost from the stored execution records were developed and executed with the assistance of Claude (Anthropic; Claude Opus 4, accessed April–May 2026; Claude Opus 5, accessed August 2026), in the Python environment specified above. The analysis script, the parser, and the evaluator-level rating data are provided as Supplementary Material (Documents S2–S4, Tables S2 and S3, Dataset S2). All outputs were reviewed and verified by the authors, who bear full responsibility for the accuracy and integrity of the reported results.

## 4. Results

### 4.1. Design and Implementation (RQ1)

**Figure 1:**
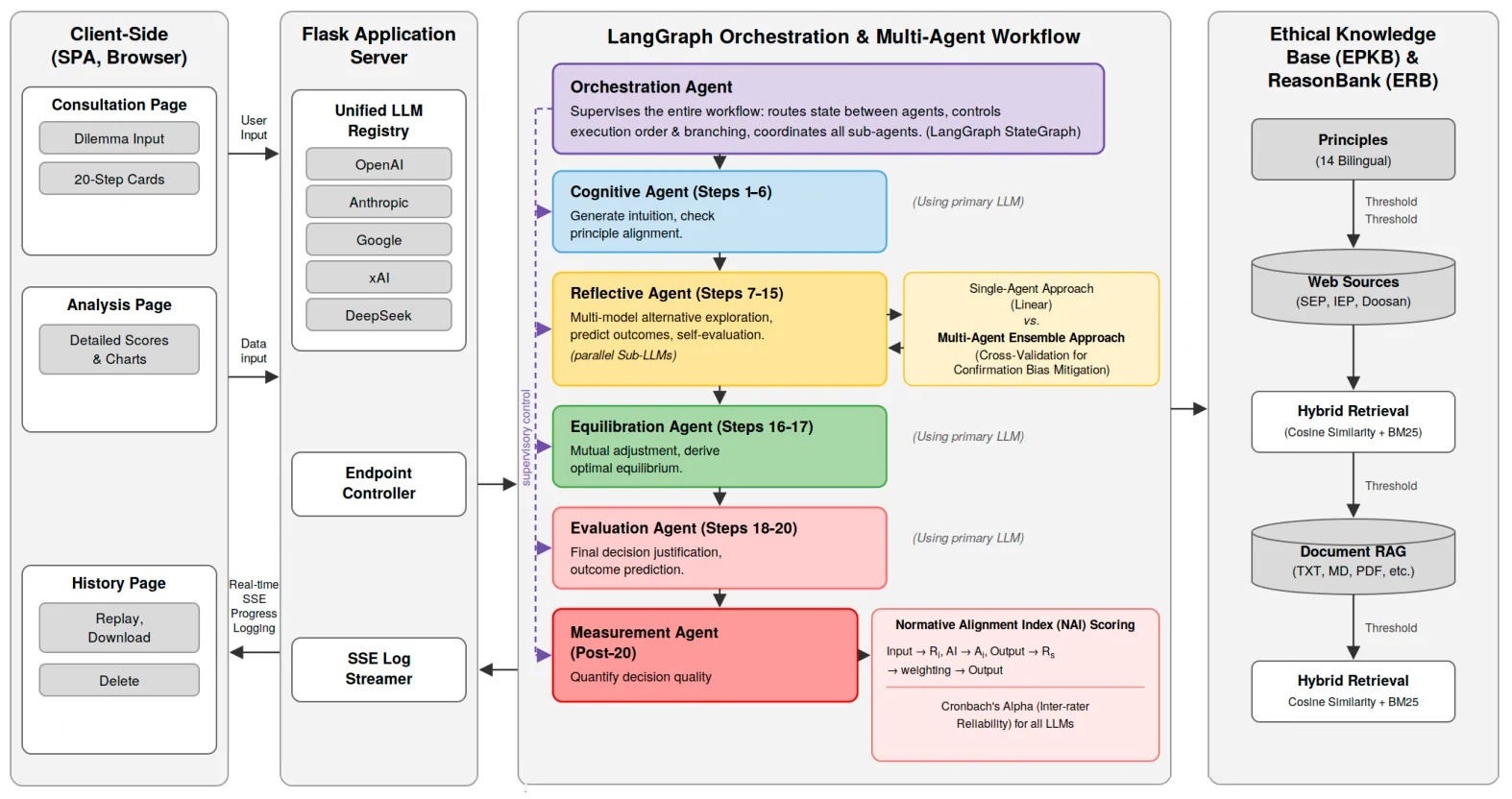
CREA system architecture: a multi-LLM, multi-agent framework for ethical decision-making.

The CREA system was implemented as a modular, multi-tier application connecting a single-page client interface to a Flask-based web server that orchestrates a LangGraph workflow engine executing the 20-step CREM procedure. The backend was built in Python (3.12+), using LangChain as a unified abstraction layer for invoking five heterogeneous LLM providers (OpenAI GPT-4.1, Anthropic Claude Haiku 4.5, Google Gemini 2.5 Flash, xAI Grok-3, and DeepSeek V3), Pydantic for type-safe agent-state and inter-agent message contracts, and Server-Sent Events (SSE) for real-time streaming of step-by-step reasoning progress to the client. All LLMs were configured with temperature = 0.0 and extended-thinking/reasoning tokens disabled, to maximize the determinism and reproducibility of outputs across repeated runs—a design requirement for the statistical comparisons reported in this study. Infrastructure-level design choices included thread-local state isolation for concurrent requests, ThreadPoolExecutor-based parallel querying for multi-LLM ensembles, and atomic (temp-file-then-rename) JSON writes to guarantee persistence integrity for each execution record (Figure 1, Document S1).

#### 4.1.1. Client Interface

**Figure 2:**
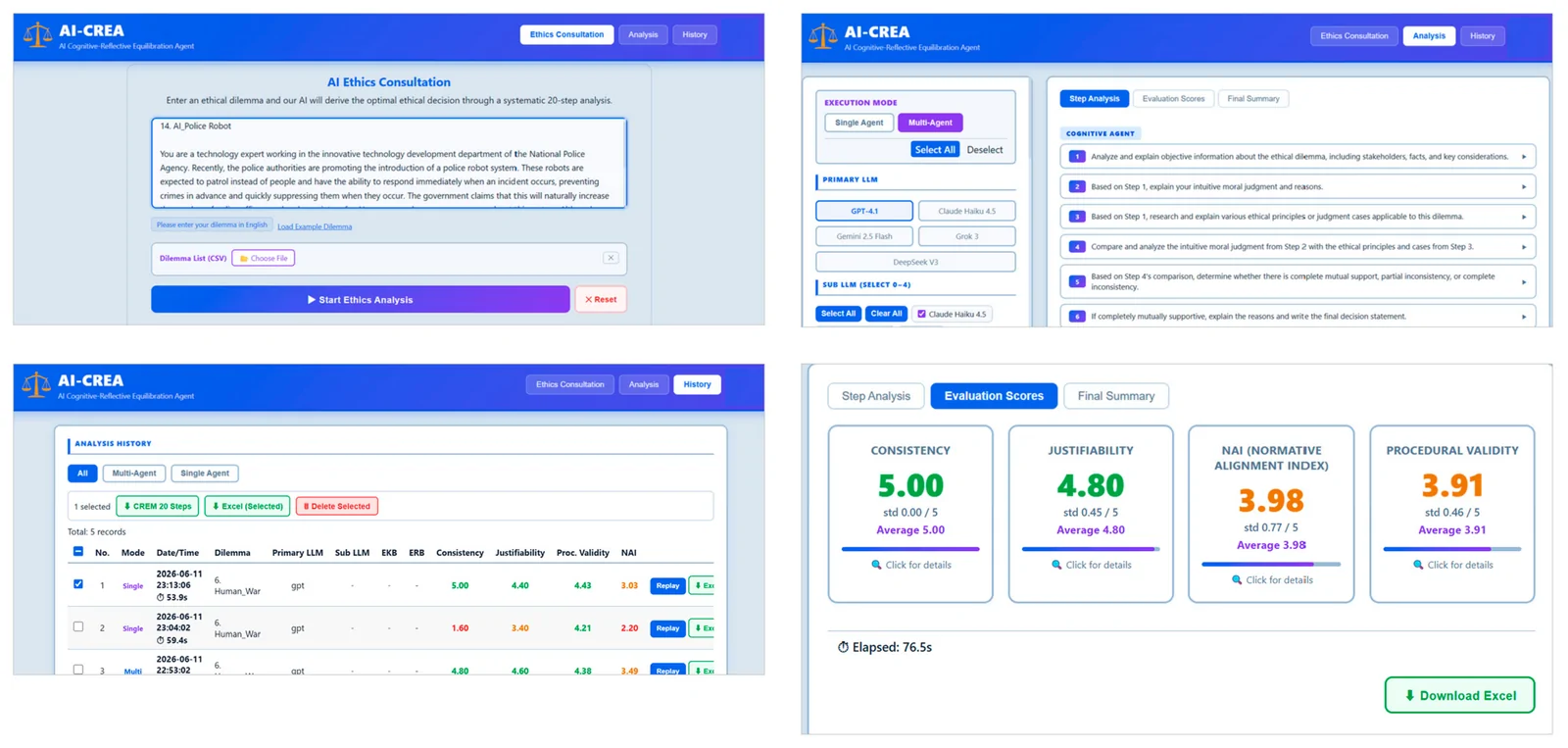
Implemented CREA client interface: Ethical Consultation Page, Analysis Page, Evaluation Scores Page, and History Page.

The client-side single-page application exposes three views: a Consultation Page, where the user enters a dilemma (or uploads a CSV batch of dilemmas), configures the LLM and operating mode, and observes a real-time step-by-step progress log alongside the resulting 20-step decision cards and evaluation scores; an Analysis Page, presenting per-LLM score breakdowns and statistical charts; and a History Page, providing replay, Excel export, and deletion of past analysis records (Figure 2, Document S1).

#### 4.1.2. Agent Architecture and Workflow

The 20-step CREM procedure was operationalized as six role-specialized agents within a LangGraph state machine: a Cognitive agent (Steps 1–6) that generates an intuitive moral judgment and checks its alignment with retrieved ethical principles; a Reflective agent (Steps 7–15) that resolves inconsistencies between intuition and principle through alternative exploration and a support/opposition scoring procedure; an Equilibration agent (Steps 16–17) that derives an optimal equilibrium under a 30-point threshold rule; an Evaluation agent (Steps 18–20) that analyzes consistency, justifiability, and predicted outcomes; and a Measurement agent that quantifies advice quality post hoc using multi-LLM ensemble scoring. A cross-cutting RAG component retrieves context from the knowledge components described below at each agent’s request.

The workflow includes three conditional branch points that route execution based on intermediate state: (1) at Step 5, a consistency check routes to the final decision statement (Step 6) if intuition and principle are mutually supportive, or to reflective analysis (Step 7) otherwise; (2) at Step 8, an adjustment-feasibility check routes to alternative exploration (Step 9) if adjustment is judged possible, or terminates with a prediction/self-evaluation (Step 10) otherwise; and (3) at Step 16, a 30-point threshold rule routes to evaluation (Step 18) if the support–opposition score differential meets or exceeds 30, or to a further mutual-adjustment step (Step 17) otherwise (Document S1).

#### 4.1.3. Knowledge Components: EPKB and ERB

Ethical grounding is provided by two retrieval-augmented components. The Ethical Principles Knowledge Base (EPKB) combines three source layers: (1) 14 built-in, bilingual ethical principles (e.g., utilitarianism, deontology, virtue ethics, care ethics, non-maleficence); (2) web sources collected dynamically (Stanford Encyclopedia of Philosophy, Internet Encyclopedia of Philosophy, Doosan Encyclopedia, and user-specified sites), filtered by BM25 relevance; and (3) document RAG over user-supplied files (.txt, .md, .pdf, .docx, .csv), retrieved at a hybrid-similarity threshold ≥ 0.45. In our implementation, the EPKB corpus is populated exclusively from dynamically crawled web sources—the Stanford Encyclopedia of Philosophy (SEP) and the Internet Encyclopedia of Philosophy (IEP)—retrieved at query time rather than from a pre-assembled static corpus. Crawled entries are cached locally to ensure consistency across experimental runs. The Ethical Reasoning Bank (ERB) stores the full result of each prior execution (dilemma, 20-step trace, evaluation scores, and metadata) as an individual JSON record, and retrieves the top three most similar past cases at a hybrid-similarity threshold ≥ 0.60, restricted to prior multi-agent runs with NAI ≥ 3.5. Both components use a hybrid retrieval score (0.6 × cosine similarity + 0.4 × normalized BM25) over paragraph-boundary-aware document chunks (500 characters, 50-character overlap), with Jaccard token overlap as a fallback when embeddings are unavailable. Pre-enrichment via EPKB/ERB retrieval occurs at Steps 3, 11, 12, 13, and 18 (Document S1).

#### 4.1.4. Multi-LLM Ensemble Design

To mitigate single-model confirmation bias, Steps 3, 11, 12, 13, and 18 employ parallel multi-LLM querying: the primary LLM generates a baseline response, auxiliary LLMs independently generate responses in parallel, and the primary LLM synthesizes all responses into a unified, attributed analysis. Independently, the Measurement agent always queries all registered LLMs—overriding any user model selection—to prevent an LLM from evaluating its own output and to preserve evaluation objectivity. This ensemble computes four quality dimensions: Consistency and Justifiability (5-point Likert scale, 5-LLM ensemble), Procedural Validity (each of the 20 steps individually scored by all 5 LLMs, aggregated as mean, SD, sum, and coefficient of variation), and the Normative Alignment Index (NAI), a composite metric weighting Ethical Relevance and Normative Alignment per principle. Cronbach’s α is computed across LLM-derived scores to quantify inter-model score consistency for each metric.

These design elements jointly define the four architectural conditions compared in this study—Single-Agent, Multi-Agent, Multi-LLM, and Multi-LLM + KRB—which differ systematically in agent specialization, LLM plurality, and access to the EPKB/ERB knowledge components, as summarized in Table 5 (Document S1).

### 4.2. Evaluation

Four architectural configurations of CREA (Single-Agent, Multi-Agent, Multi-LLM, and Multi-LLM + KRB) were compared on four core evaluation indicators—Consistency, Justifiability, Procedural Validity, and the Normative Alignment Index (NAI)—and on the overall mean of these indicators. All comparisons were based on 500 matched execution units per architecture (N = 500 per condition). This evaluation is organized as a descriptive comparison of the four architectures, pairwise inferential tests, internal-consistency (Cronbach’s α) analyses, adjacent-stage α-difference tests, and an extended distributional analysis based on box plots.

#### 4.2.1. Level of Advice Quality Across Architectures (RQ2)

**Figure 3:**
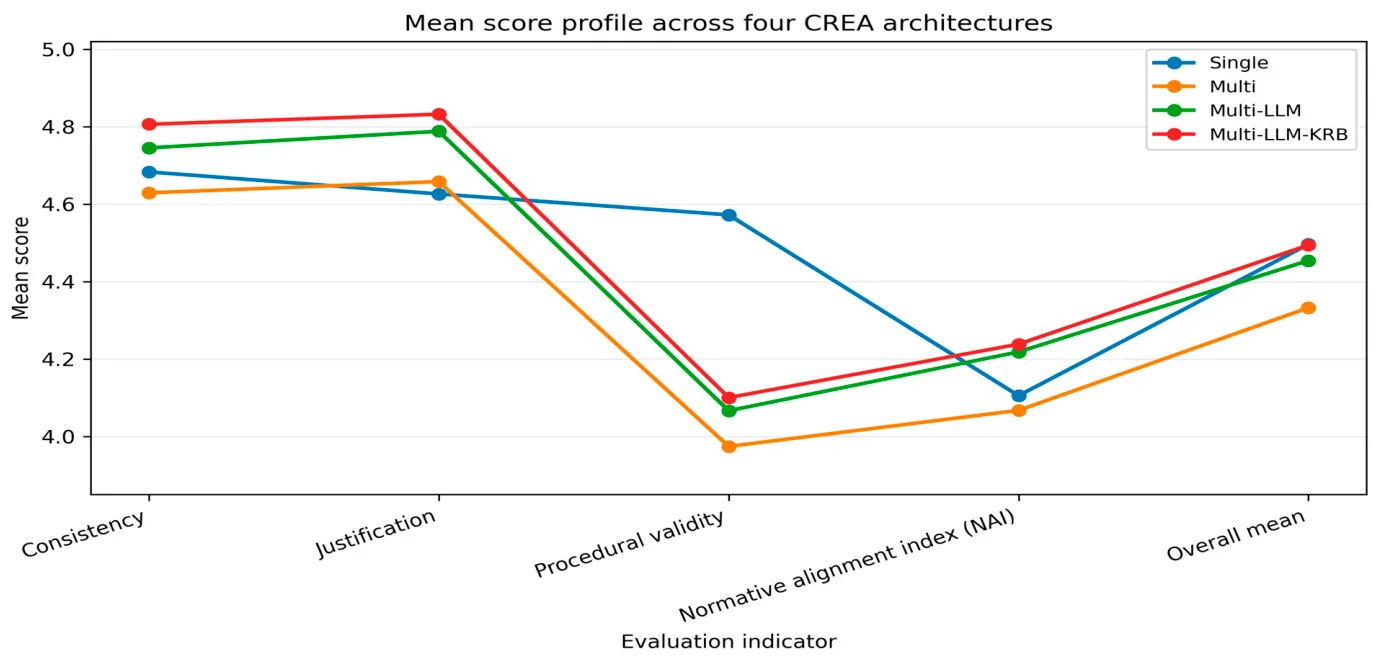
Mean score profiles across the four CREA agent architectures.

**Figure 4:**
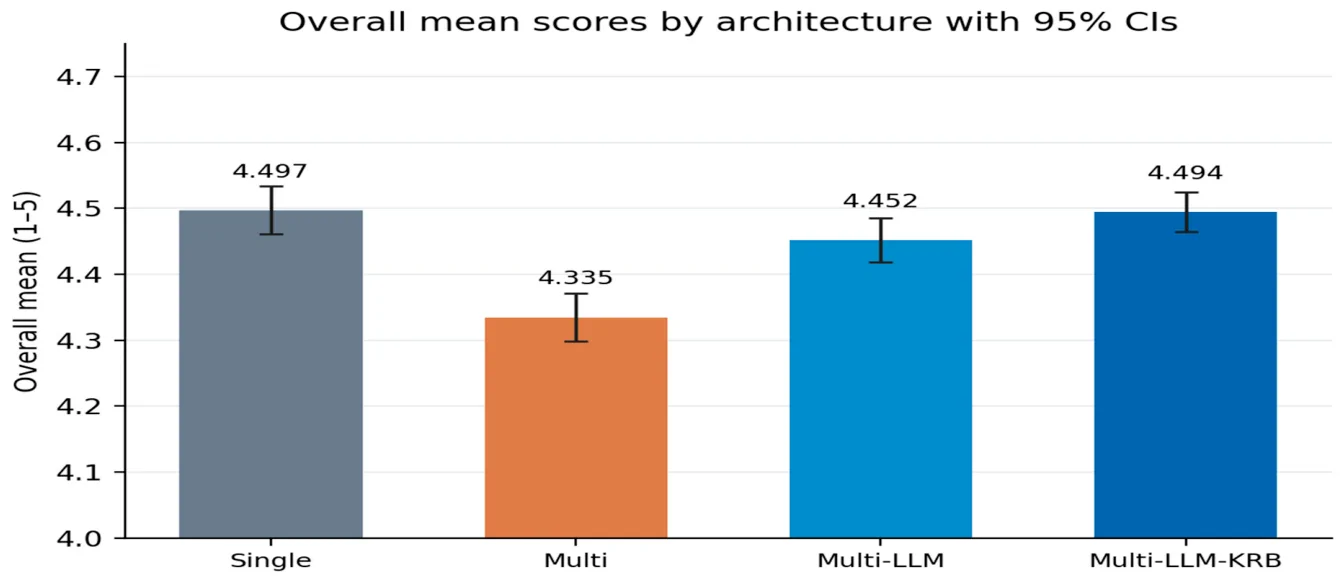
Overall mean scores by architecture with 95% confidence intervals.

This subsection reports the performance of the four architectures on each indicator without comparing them inferentially. Table 6 and Table 7 give means, standard deviations, and coefficients of variation for the four indicators and their overall mean; Figure 3 and Figure 4 display the mean-score profiles. System-internal scores are high throughout, and the distributions are compressed near the scale maximum on consistency and justifiability, so ceiling effects should be assumed when interpreting the differences reported in Section 4.2.2. The Multi-LLM + KRB architecture produced the highest means for Consistency (M = 4.806), Justifiability (M = 4.832), and NAI (M = 4.238). Procedural Validity, however, remained highest in the Single-Agent condition (M = 4.574), whereas the baseline Multi-Agent condition showed the lowest Procedural-Validity mean (M = 3.975).

#### 4.2.2. Pairwise Comparisons Among Architectures (RQ3)

**Figure 5:**
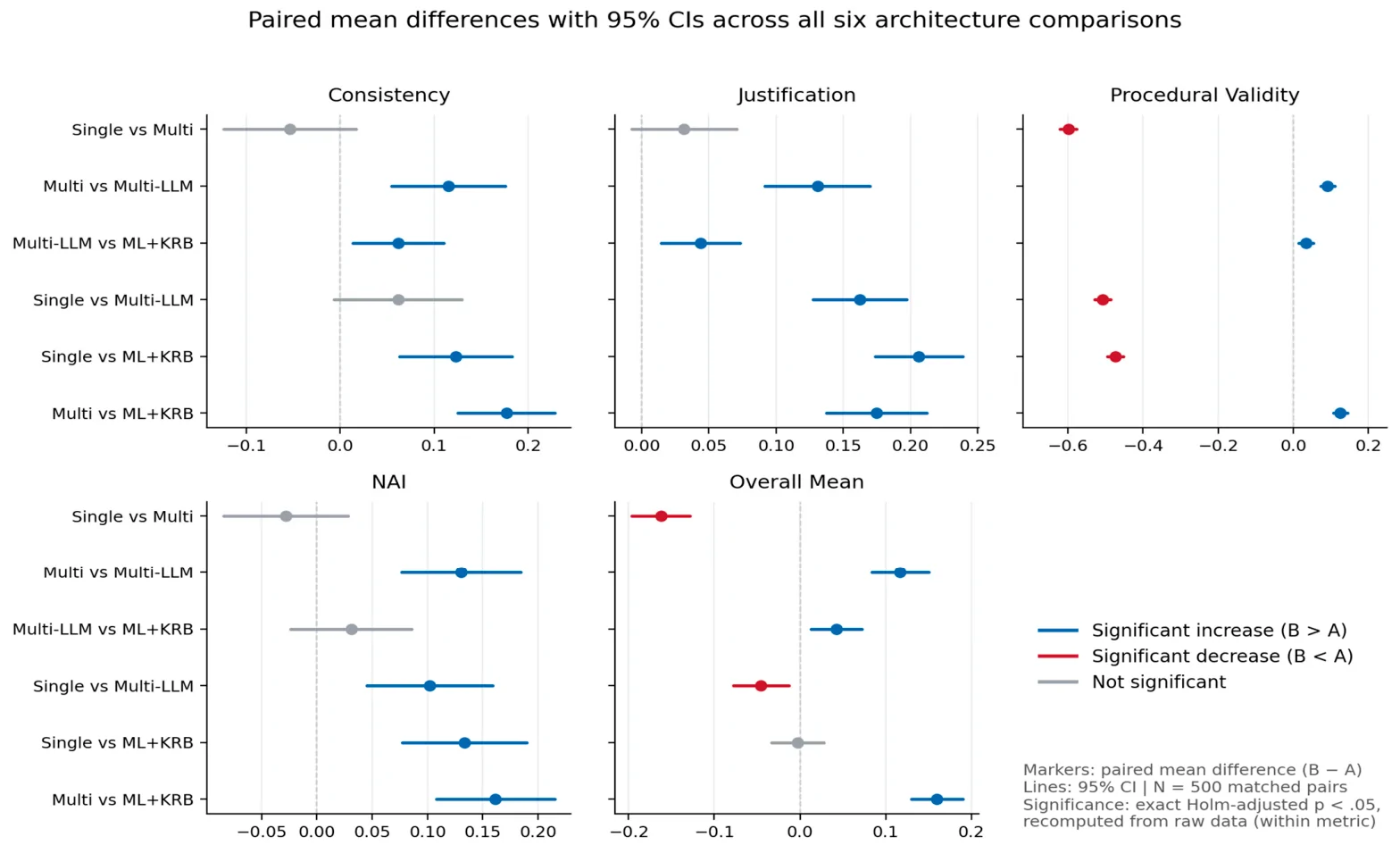
Paired mean differences with 95% confidence intervals for all six architecture comparisons. Blue = significant increase, red = significant decrease, gray = not significant after Holm correction.

**Figure 6:**
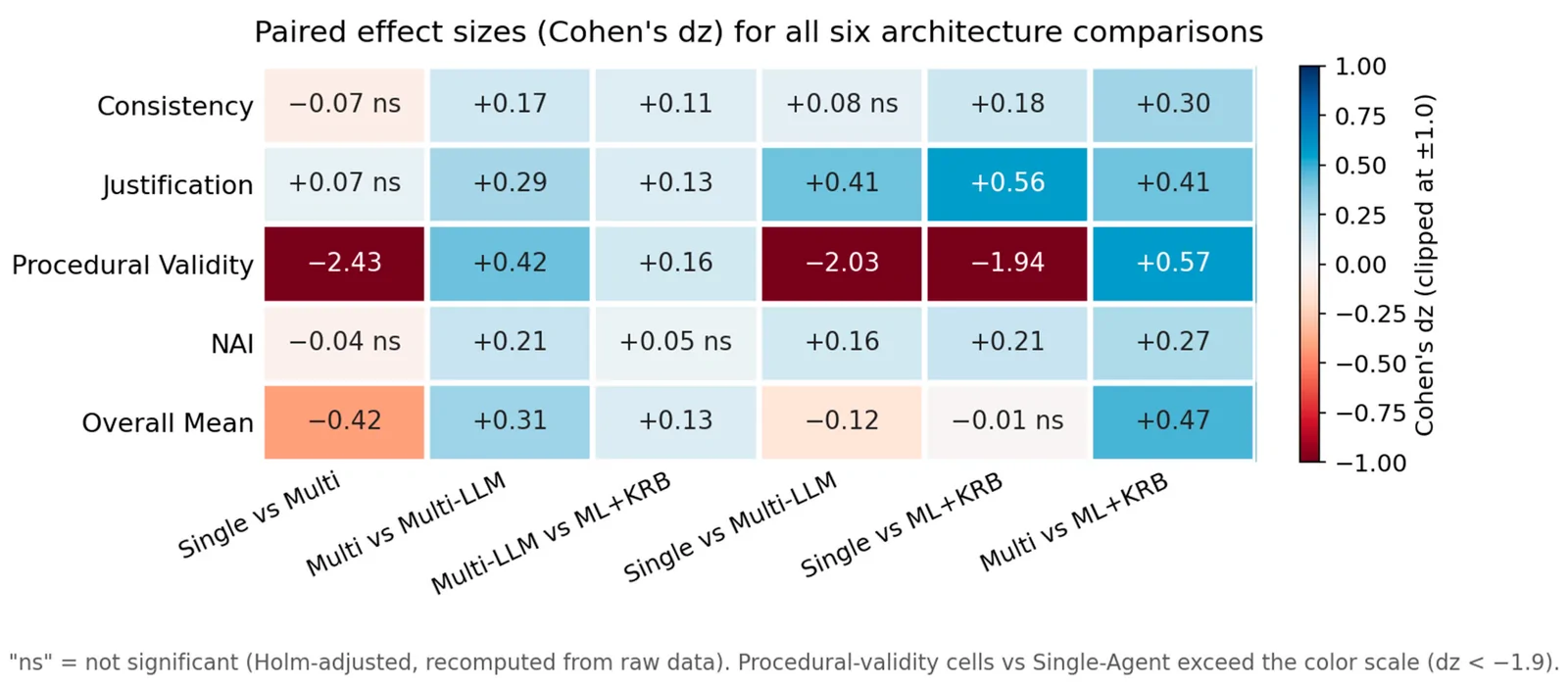
Paired effect sizes (Cohen’s dz) for all six architecture comparisons across the five outcome measures.

This subsection reports the six pairwise architecture comparisons for each indicator and answers RQ3. Primary analyses are paired-samples *t*-tests over the 500 matched execution units with Holm correction applied within each metric across the six comparisons; Wilcoxon signed-rank tests are reported as a distribution-free check and Cohen’s dz as the paired effect size. The complete set of comparisons is reported in Table 8 and visualized in Figure 5 and Figure 6. Effect sizes are described as small, medium, or large following Cohen’s convention (dz ≈ 0.2/0.5/0.8).

Table 8 reports all six pairwise contrasts among the four architectures for each indicator, with Holm correction applied within each metric. Along the incremental construction path, the transition from single-agent to the baseline multi-agent condition produced no significant change in consistency, justifiability, or NAI, but a large, significant reduction in procedural validity (Δ = −0.599, dz = −2.435) that carried the overall mean downward (Δ = −0.162, dz = −0.417). Extending the multi-agent condition with multi-LLM deliberation significantly improved all five outcomes (dz = 0.168–0.424), and adding KRB produced further small but significant gains in consistency, justifiability, procedural validity, and the overall mean (dz = 0.112–0.157), with no significant change in NAI (Δ = +0.031, *p* = .264).

The cumulative contrasts against the single-agent baseline summarize the net effect of the full architectural development. Relative to single-agent, the complete multi-LLM + KRB configuration scored significantly higher on consistency (Δ = +0.123, dz = 0.181), justifiability (Δ = +0.206, dz = 0.559), and NAI (Δ = +0.133, dz = 0.208), while remaining significantly lower on procedural validity (Δ = −0.474, dz = −1.936); no significant difference was detected on the overall mean (Δ = −0.003, *p* = .859). We emphasize that a non-significant difference does not establish equivalence: demonstrating that the two configurations are practically indistinguishable would require a formal equivalence test against a prespecified, justified margin, which we did not conduct. Descriptively, the KRB component reduced the overall-mean deficit relative to single-agent from −0.045 to −0.003 and narrowed the procedural-validity gap by 0.034 points. Apart from the procedural-validity contrasts against single-agent (|dz| ≈ 1.9–2.4), every significant difference corresponds to a small-to-moderate effect (|dz| ≤ 0.57), and several statistically significant contrasts—particularly those introduced by KRB—are small enough that their practical importance should be interpreted as modest.

One further qualification concerns consistency in the single-agent versus multi-agent comparison. The Wilcoxon test returned *p* < .001 despite a negligible mean difference, which reflects a ceiling effect (218 pairs tied at the maximum of 5.0) together with a directional asymmetry among the 282 non-tied pairs (177 multi < single vs. 105 multi > single). It should not be read as a substantively important difference.

The procedural-validity contrasts require a methodological caveat before they are interpreted. As described in Section 3, the two families of conditions were not scored the same way. Single-agent executions were evaluated with all twenty steps presented together, so an evaluator judging a given step could see the steps it refers to; multi-agent, multi-LLM, and multi-LLM + KRB executions were evaluated one step at a time, with only that step supplied. Steps whose procedural adequacy depends on their linkage to earlier judgments were therefore assessed without access to that linkage in three of the four conditions. The observed gap consequently reflects a difference in measurement design as well as any difference in execution, and Section 5 examines the extent to which each contributes.

Because execution units are nested within dilemma cases, linear mixed-effects models were fitted as robustness analyses for each comparison, with the architecture condition as the fixed effect of interest, executor LLM and execution round as additional fixed effects (each with five levels, at or below the number of groups usually recommended for stable variance-component estimation), and dilemma case as a random intercept. Between-dilemma variance was substantial: the dilemma random intercept accounted for approximately 39 to 43 percent of the variance in NAI and 47 to 50 percent in the overall mean, indicating that some dilemmas elicited systematically different scores across all conditions. The architecture effects nevertheless reproduced the paired-test significance pattern in all thirty comparisons (Table 9), so clustering within dilemmas does not account for the differences we report.

#### 4.2.3. Internal Consistency of Architecture-Level Measurement Scores

**Figure 7:**
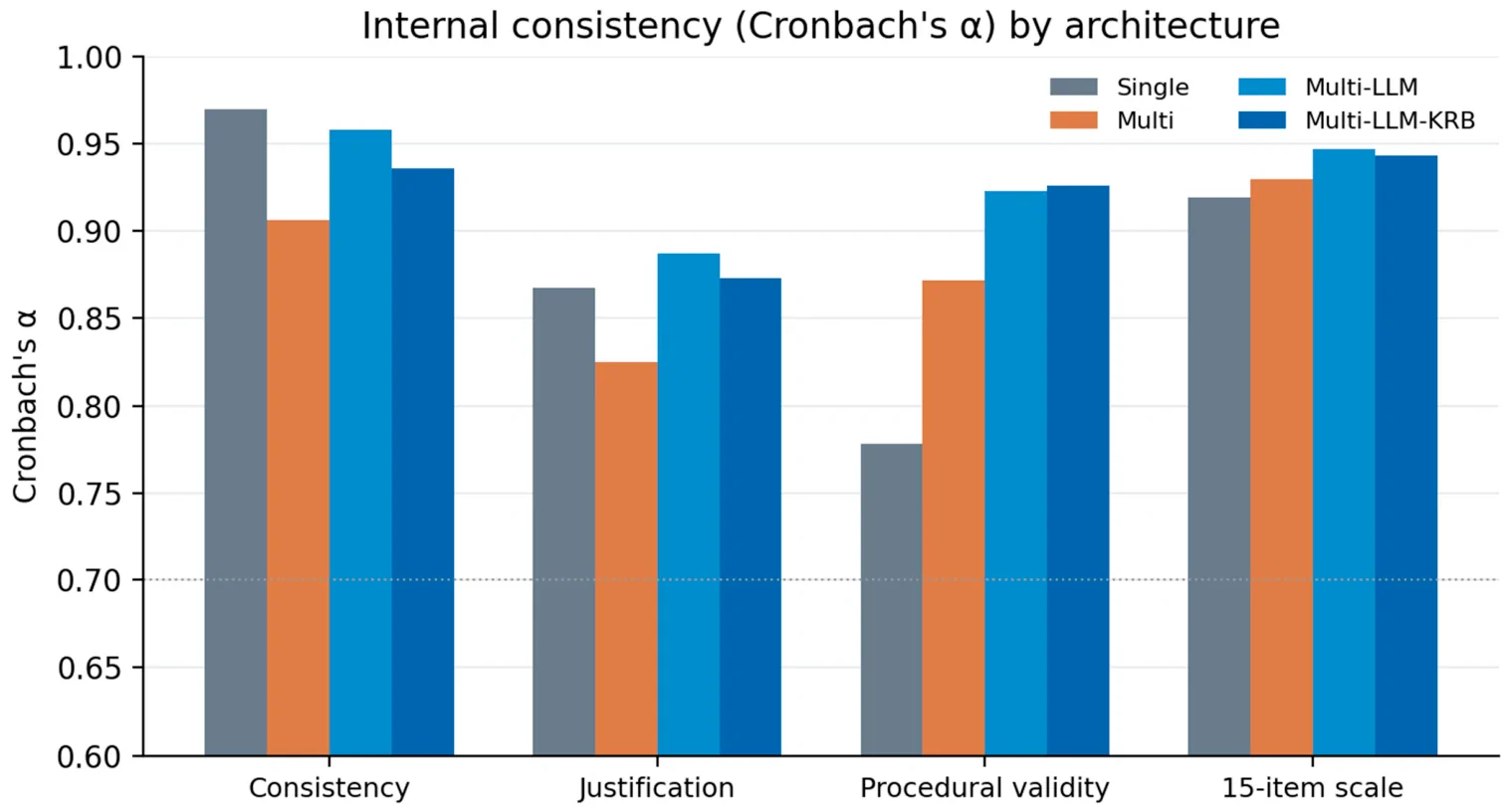
Metric-specific internal consistency (Cronbach’s α) by architecture.

Cronbach’s α was used to examine the internal consistency of the LLM-derived measurement scores within each architecture (Table 10; Figure 7), rather than to provide direct evidence of evaluator reliability or normative correctness. At the metric-specific level, Consistency showed excellent internal consistency across all architectures (α = 0.906–0.969), and Justifiability remained in the good range (α = 0.825–0.887). Procedural-Validity α rose from acceptable in the Single-Agent condition (α = 0.778) to good or excellent in the multi-agent variants, reaching α = 0.926 in Multi-LLM + KRB. For the 15-item LLM-derived measurement scale, all architectures showed excellent internal consistency (α = 0.919–0.947). For the execution-level composites, Single-Agent showed weaker internal consistency than the multi-agent variants, most notably for the three-indicator scale excluding NAI (α = 0.670).

#### 4.2.4. Differences in Cronbach’s α Across Architecture Development Stages

The analyses in this subsection are secondary and exploratory. They characterize the behavior of the measurement instrument rather than the architectures, and they were not planned before data collection. Differences in Cronbach’s α between conditions were tested with paired bootstrap confidence intervals and paired permutation tests (3000 resamples each, seed 20260805); we report them because a change in measurement coherence across conditions is itself informative, not as evidence about advice quality.

Adjacent-stage α-difference tests characterized how measurement consistency changed as the architecture was extended (Table 11). Moving from Single-Agent to Multi-Agent significantly reduced α for Consistency but significantly increased Procedural-Validity α and the execution-level composite α values. Moving from Multi-Agent to Multi-LLM significantly increased Consistency α, Procedural-Validity α, and the 15-item measurement-scale α. However, moving from Multi-LLM to Multi-LLM + KRB produced no significant additional α gains. The KRB extension should therefore be interpreted primarily as improving mean-level performance rather than fundamentally altering the internal consistency of the measurement scores.

#### 4.2.5. Extended Descriptive Statistics and Distributional (Box-Plot) Analysis

To interpret the score distributions more directly, the descriptive analysis was extended to include the minimum, median, and maximum in addition to the mean and standard deviation (Table 12). Across the four architectures, Multi-LLM + KRB showed the highest median for the overall mean and reached the highest median for Consistency and Justifiability (tied with other high-scoring conditions), whereas the highest NAI median was observed in the Multi-LLM condition (4.390) and Single-Agent retained the highest median Procedural Validity. For the overall mean, the medians were 4.606 (Single), 4.420 (Multi), 4.589 (Multi-LLM), and 4.615 (Multi-LLM + KRB); the corresponding minima were 2.927, 2.842, 2.964, and 3.094, and the maxima were 4.989, 4.938, 4.920, and 4.933. Multi-LLM + KRB thus achieved the highest central tendency while maintaining a relatively compressed, stable overall-score range, whereas the baseline Multi architecture showed the lowest central tendency and the weakest lower-tail performance.

**Figure 8:**
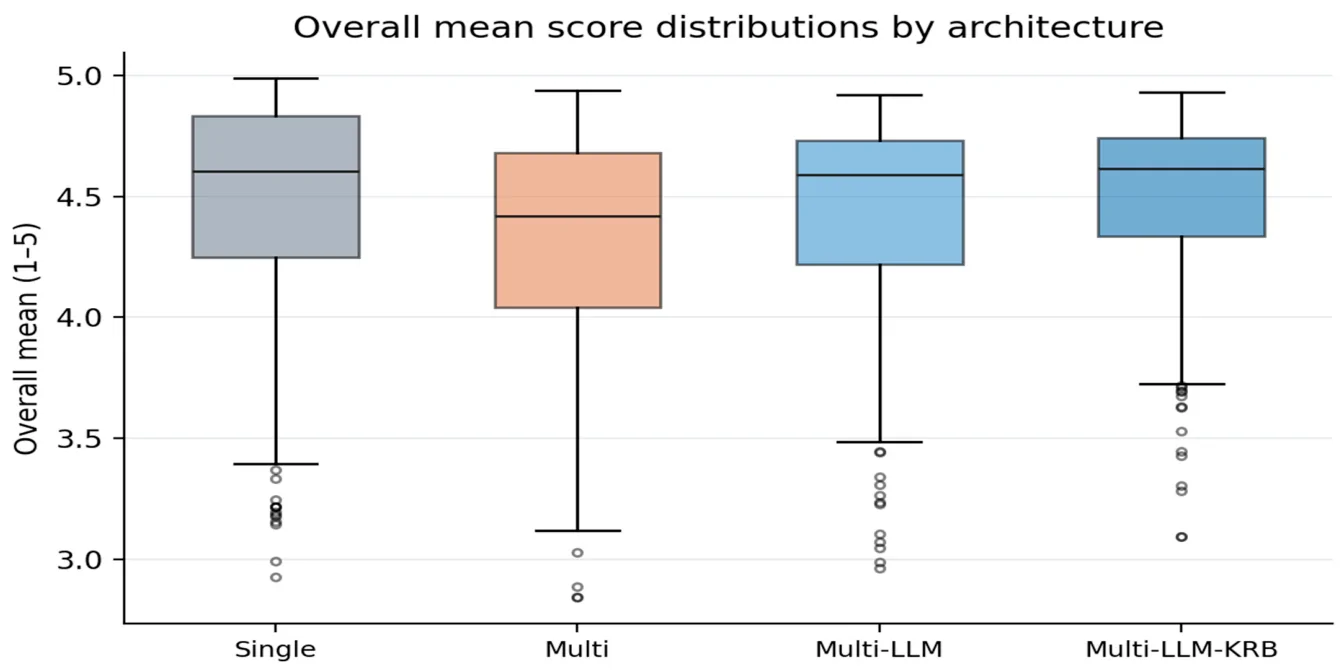
Box plot of overall mean scores across the four architectures. The central line is the median, the box the interquartile range, and the whiskers the range of values excluding outliers; circles denote outliers.

**Figure 9:**
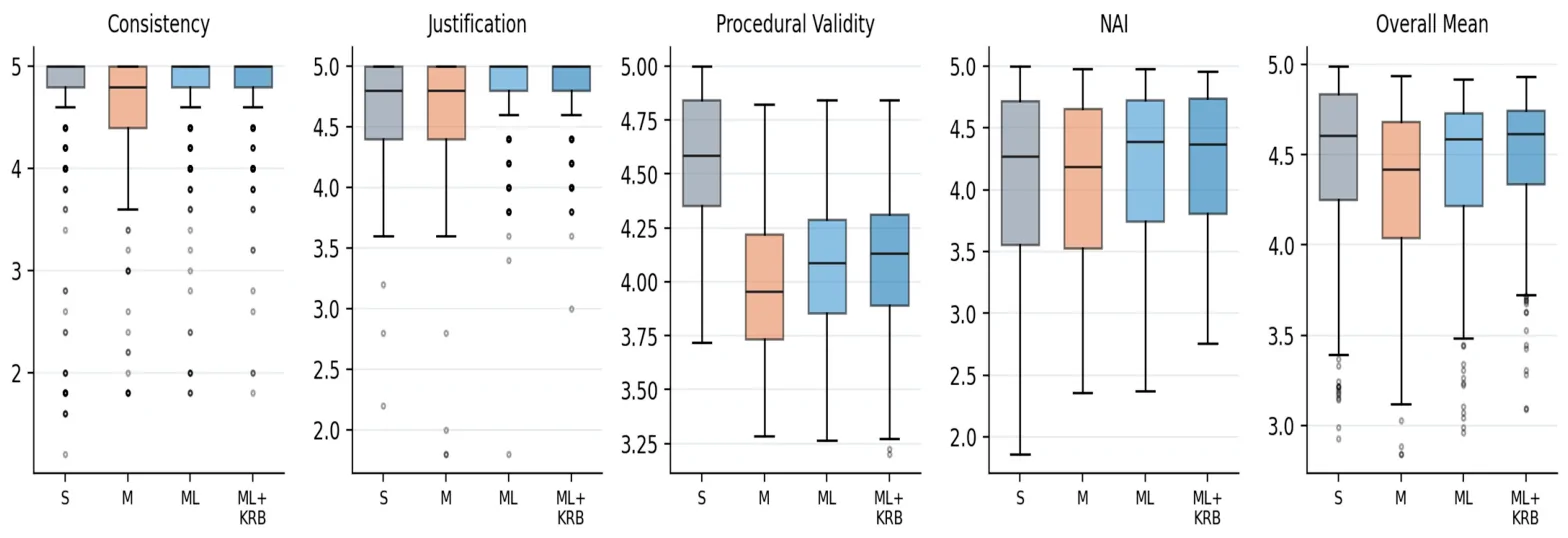
Metric-wise box-plot comparison of the four architectures across Consistency, Justifiability, Procedural Validity, NAI, and Overall Mean. In the box plot image, the circles represent outlier values other than the minimum, center, and maximum.

The box plots make these distributional contrasts explicit. Figure 8 presents the overall-mean distributions: Multi-LLM + KRB occupies the highest median position with a relatively narrow interquartile range, Single-Agent shows a comparably high median but several lower outliers, the baseline Multi architecture exhibits the lowest median and widest lower spread, and Multi-LLM improves substantially over Multi. Figure 9 provides the metric-wise comparison. For Procedural Validity, the Single-Agent distribution is clearly shifted upward relative to the other architectures, whereas for Consistency, Justifiability, NAI, and the overall mean, Multi-LLM + KRB tends to occupy the highest position with medians clustered near the top of the scale. Together, the extended descriptive statistics and box plots support the interpretation that Multi-LLM + KRB provides the strongest balance of high central tendency and distributional stability, while Single-Agent retains a distinct advantage in procedural adherence.

#### 4.2.6. Reported Call Counts, Tokens, Latency, and Cost

**Figure 10:**
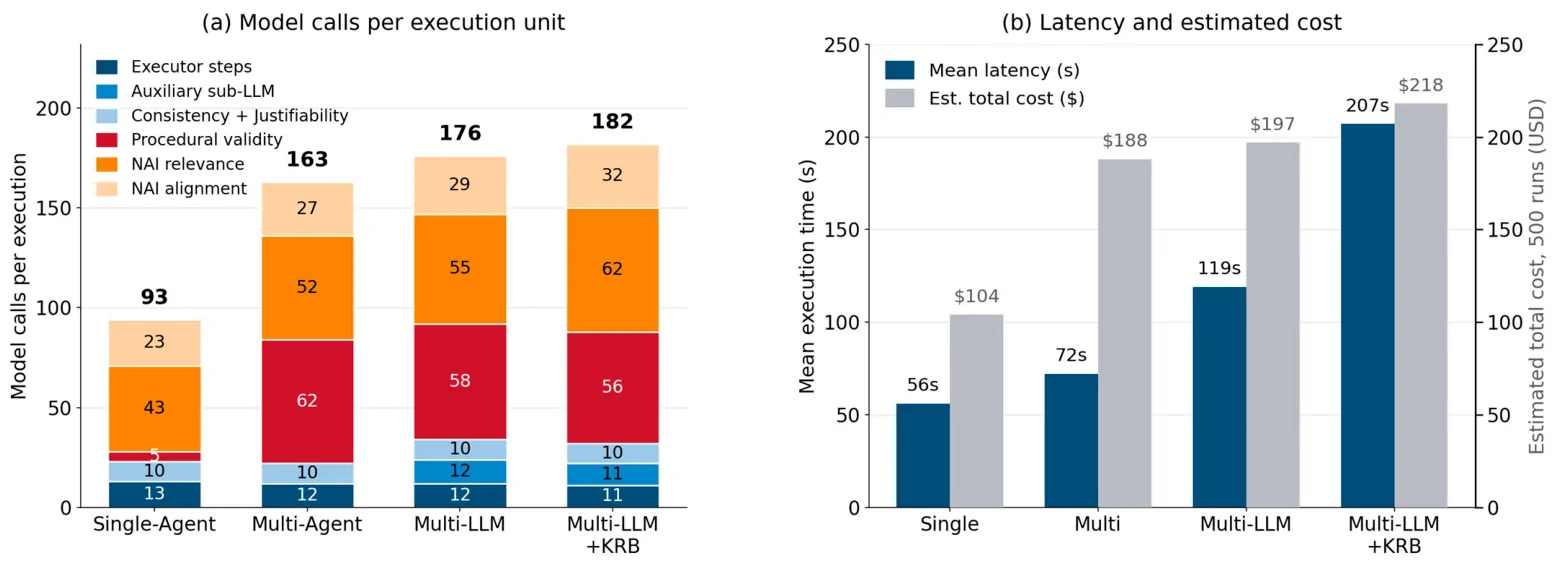
(**a**) Model calls per execution unit by component; (**b**) mean latency and estimated total cost for 500 executions.

Because the conditions were not matched on inference budget, we quantified their computational expenditure from the complete execution records. Wall-clock time per execution rose monotonically along the development path: 56.1 s (SD = 41.2) for single-agent, 72.5 s (SD = 58.5) for multi-agent, 119.1 s (SD = 98.4) for multi-LLM, and 207.3 s (SD = 269.6) for multi-LLM + KRB, that is, 1.29, 2.12, and 3.70 times the single-agent baseline. Model calls and tokens are reported in Table 13, Table 14 and Table 15 and Figure 10. Three observations qualify interpretations based on inference budget. First, the executor-side workload is comparable across conditions—12.5 to 23.5 calls per execution—so the architectures do not differ markedly in the amount of deliberation they perform. Second, the measurement pipeline dominates expenditure in every condition, issuing 80 to 160 calls per execution and accounting for 64 to 76 percent of estimated cost; the largest single component in the multi-agent family is the per-step procedural-validity scoring described in Section 5.2.3. Third, the estimated totals (approximately USD 104, 188, 197, and 218 for 500 executions) therefore reflect the evaluation regime at least as much as the architecture. Inference budgets were nonetheless not experimentally controlled, so improvements attributable to architecture cannot be fully separated from those attributable to greater computational expenditure.

#### 4.2.7. Summary of Findings

Taken together, the results support a differentiated interpretation of CREA architecture development rather than a single dominant configuration. The baseline Multi-Agent architecture did not outperform Single-Agent execution and was limited mainly by reduced Procedural Validity. The Multi-LLM extension significantly improved the baseline Multi-Agent architecture across all measured domains. The additional KRB component produced further significant gains in Consistency, Justifiability, Procedural Validity, and the overall mean, while leaving NAI statistically unchanged relative to Multi-LLM. Overall, Multi-LLM + KRB emerged as the highest-scoring architecture for justifiability- and alignment-oriented performance and did not differ significantly from Single-Agent on the overall mean (Δ = −0.003, *p* = .859; Table 8), whereas Single-Agent remained strongest for strict procedural-validity preservation. This pattern indicates that architectural expansion can improve measured advice quality (consistency, justifiability, NAI) and the internal consistency of the measurement scores (Cronbach’s α; Table 8, Table 10 and Table 11) when supported by multi-LLM execution and KRB resources, but that procedural-control mechanisms remain necessary to preserve stepwise fidelity. These findings are summarized by research question in Table 16.

## 5. Discussion

### 5.1. Principal Findings in Relation to the Research Questions

This study addressed the cognitive grounding problem in artificial moral advising by translating the Cognitive–Reflective Equilibration Model (CREM) into an executable multi-LLM, multi-agent architecture. Regarding RQ1, CREM was operationalized through a six-role structure comprising an orchestration agent, four stage-aligned reasoning agents, and a measurement agent. The Cognitive, Reflective, Equilibration, and Evaluation agents implement the four CREM stages while preserving the 20-step sequence; multi-LLM deliberation broadens the range of principles, counterarguments, and supporting and opposing reasons considered; and the knowledge/reasoning-bank augmentation (KRB), consisting of the Ethical Principles Knowledge Base (EPKB) and the Ethical Reasoning Bank (ERB), supplies external normative resources and reusable prior reasoning. CREA thereby operationalizes cognitive grounding as a traceable process linking intuitive judgment, normative comparison, conflict recognition, mutual adjustment, and outcome evaluation.

Regarding RQ2, all four configurations received generally high system-internal advice-quality scores, but their strengths differed by dimension. Multi-LLM + KRB achieved the highest means for Consistency (M = 4.806), Justifiability (M = 4.832), and the Normative Alignment Index (NAI; M = 4.238), whereas Single-Agent retained the highest Procedural Validity (M = 4.574). The 15-item LLM-derived measurement scale showed excellent internal consistency in every condition (α = 0.919–0.947), and metric-specific coefficients ranged from acceptable to excellent (minimum α = 0.778). These coefficients indicate coherent measurement patterns rather than normative correctness or conventional inter-rater reliability. The results support the feasibility of generating advice that is consistent, reason-giving, procedurally assessable, and normatively aligned under system-internal evaluation, while confirming that AMA quality is multidimensional.

Regarding RQ3, adding agents was not by itself sufficient to improve moral advice. Relative to Single-Agent, the baseline Multi-Agent condition did not significantly improve Consistency, Justifiability, or NAI, and it substantially reduced Procedural Validity (Δ = −0.599, dz = −2.435) and the overall mean (Δ = −0.162). Adding multi-LLM deliberation then significantly improved all four core indicators and the overall mean, and KRB produced further, generally small, gains in Consistency, Justifiability, Procedural Validity, and overall performance, though not in NAI. Multi-LLM + KRB did not differ significantly from Single-Agent on the overall mean (Δ = −0.003, *p* = .859), while exceeding Single-Agent in Consistency, Justifiability, and NAI. Thus, the results did not support an advantage for agent separation alone but did indicate an advantage for multi-LLM deliberation, and the incremental benefit of externalized knowledge and reasoning memory received partial support.

### 5.2. Theoretical Implications

#### 5.2.1. Cognitive Grounding and Computational Reflective Equilibrium

A primary theoretical contribution of CREA is a concrete mechanism for the mutual adjustment central to reflective equilibrium. Rawls explains moral justification as coherence among considered judgments, principles, and background theories, but does not prescribe an algorithmic procedure for achieving it. CREM supplements this structure with Piagetian equilibration, in which conflict produces disequilibrium and motivates assimilation, accommodation, and movement toward a better equilibrium (Piaget, 1975/1985; Rawls, 2017). Its 20 steps record where intuition conflicts with principle, how alternatives are generated, which reasons support or oppose them, and how the final judgment is selected or adjusted. This extends coherence-based computational reflective-equilibrium models organized around a single reasoning engine (Yilmaz et al., 2017), and it evaluates justifiability as an observable quality rather than assuming that ethical vocabulary entails sensitivity to reasons, consistent with normative approaches emphasizing reasons, transparency, and contextual adaptability (Behdadi & Munthe, 2020). CREA does not establish moral truth; it contributes an auditable procedure through which reasons can be inspected, challenged, and revised by a human decision-maker.

#### 5.2.2. Comparative Interpretation with Prior Work

First, relative to coherence-driven computational models of reflective reasoning (Yilmaz et al., 2017; Wallach et al., 2010), CREA operationalizes their single-engine proposals as a 20-step procedure executed by role-differentiated agents. The comparisons qualify the distribution hypothesis: bare agent decomposition (Multi-Agent) did not outperform Single-Agent execution, whereas distribution supported by multi-LLM execution and KRB resources did not differ significantly from Single-Agent on the overall mean and significantly surpassed it on Consistency, Justifiability and NAI.

Second, relative to general formal frameworks for ethical reasoning (Conitzer et al., 2017; Machado et al., 2024), CREA adjudicates through reason comparison rather than a single unified framework and measures four indicators separately. This separation was diagnostically informative, revealing that the two strongest architectures did not differ significantly on the overall mean while excelling on different indicators (Table 8)—a pattern an aggregate framework would have collapsed.

Third, relative to multi-agent conceptions of machine ethics (Murukannaiah et al., 2020; Mashayekhi et al., 2022; Yamani et al., 2025), CREA supplies the concrete deliberative mechanism this strand left unspecified, and its stepwise architectural decomposition and step-level traces directly address the variability and verification problems Yamani et al. identified. Mashayekhi et al.’s caution that consensus must not be equated with ethicality anticipates our scope condition: all gains were measured under LLM-based self-evaluation, not normative validity.

Fourth, relative to single-theory operationalizations (Stenseke, 2024; Woodgate, 2025), CREA targets coherent adjustment among plural principles and shows this improves with deliberative resources—yet Single-Agent execution remained strongest in strict procedural fidelity, suggesting a partial tension between principled pluralism and stepwise control.

Taken together, CREA connects conceptual strands that provide theoretical grounding without distributed implementation with computational strands that provide distributed implementation without coherence-theoretic grounding, yielding a differentiated verdict rather than a single dominant configuration: resourced distribution yields justification quality and normative alignment, single-agent execution retains stepwise fidelity, and translation into scaffolded human judgment remains a hypothesis for user-centered validation.

#### 5.2.3. The Trade-Off Between Procedural Fidelity and Deliberative Expansion

The most conspicuous finding is the procedural-validity gap between single-agent execution and every multi-agent configuration (Δ = −0.599 against the multi-agent baseline; −0.474 against the full configuration). Procedural validity was not measured in the same way across the two families. In the single-agent condition the Measurement Agent assembled all twenty step slots into one prompt and asked each evaluator to score every executed step within that context. In the multi-agent conditions it issued a separate query for each executed step, supplying that step’s output and its procedural requirement without the surrounding trace—45–90 such queries per execution. Several of the twenty requirements are intrinsically relational: Step 4 asks whether the intuitive judgment of Step 2 has been correctly compared with the principles retrieved at Step 3; Step 18 asks whether the final decision remains consistent with the initial intuition. An evaluator shown only the text of such a step cannot verify the relation it is supposed to instantiate and must rely on the surface adequacy of the isolated step presented to it. Systematically lower scores are the expected consequence of that design, independent of how the steps were actually executed.

We therefore interpret the reduced procedural-validity scores as substantially an artifact of context-free per-step measurement rather than as direct evidence that multi-agent execution degrades procedural fidelity. Three considerations keep this interpretation appropriately modest. First, an execution-level contribution cannot be excluded: measurement design and architecture covary in these data, and only a context-inclusive re-evaluation of the stored traces would separate them. Second, the single-agent instrument was not itself unconstrained—the bundled prompt truncated each step to its first 400 characters, so roughly half of each step reached the evaluator—which means neither regime observed the procedure in full. Third, and most informative, the ordering within the multi-agent family is measured under a constant regime: multi-LLM deliberation improved procedural validity over the multi-agent baseline (Δ = +0.092, dz = 0.424) and the knowledge and reasoning banks improved it further (Δ = +0.034, dz = 0.157). Where measurement was held constant, added deliberative resources were associated with better, not worse, procedural adherence.

Two independent lines of evidence support this account. The measurement code paths differed, as the stored records show: single-agent records carry a per-step executed flag that the multi-agent records lack, indicating different serialization and therefore different code. And the evaluator outputs themselves differ in the ways that a bundled reply and a standalone reply differ—procedural-validity reasons average 167 characters in the single-agent condition against 433 to 438 in the multi-agent conditions, 1.3 percent of single-agent reasons are empty after parsing against none in the multi-agent conditions, and 0.8 per cent of single-agent reasons restate the step label against 55 to 58 per cent of multi-agent ones, as expected for a standalone response to a single-step query (Table 17). Accordingly, we do not claim that a monolithic agent preserves procedural fidelity better than a distributed one; we claim that the present measurement cannot support that comparison, and we identify a context-inclusive re-evaluation of the stored execution traces as the highest priority for future work. Orchestration quality—state schemas, mandatory step checks, context-integrity validation—remains an important design requirement for procedural integrity, but it must be evaluated with an instrument that respects the procedure’s relational structure.

#### 5.2.4. Contribution to Artificial Moral Advisor Theory

CREA is best understood as an explicit, functional moral advisor rather than a fully autonomous moral agent: it identifies morally relevant features, compares principles, and offers a reasoned recommendation, but authority and responsibility remain with the human user (Formosa & Ryan, 2021; Misselhorn, 2022; Queloz, 2025a, 2025b). The architecture also provides a technical implementation of the ideal-observer of informed and consistent advice (Giubilini & Savulescu, 2018). No LLM can be assumed genuinely impartial, but independent model responses and cross-model synthesis reduce dependence on any single model’s framing. Multi-LLM operation is therefore intended as a structural device for plural normative deliberation, corresponding to wide reflective equilibrium’s weighing of alternative conceptions. The improvement from multi-agent to multi-LLM across every outcome is consistent with that intention, but we did not measure whether the alternatives generated by different models are normatively distinct, so this remains an interpretation of the result pattern rather than a demonstrated mechanism. KRB further externalizes normative memory: the EPKB supplies retrievable principles and sources, while the ERB makes prior dilemmas and reasoning traces available to later runs. Because its effect was modest and did not significantly increase NAI, it should not be described as autonomous moral learning. Since that component does not update model weights, we do not characterize this as normative memory or moral learning.

### 5.3. Confounds and Alternative Explanations

Before the comparisons reported in Section 4 are given a causal reading, five features of the design warrant explicit consideration. For three of them we can report measurements that bound the concern; the remaining two are properties of a system-internal evaluation and cannot be resolved within such an evaluation.

First, evaluator overlap. The five models that generated the advice also scored it, and the overlap is total rather than partial: the measurement stage enabled the full five-model registry irrespective of which model executed the run (Section 3.4), so every execution was scored by an ensemble that included its own executor. The high internal-consistency coefficients reported in Section 4.2.3 are therefore compatible with two readings we cannot distinguish—convergent judgment about advice quality, or agreement produced by biases shared across models trained on overlapping corpora. We regard this as the most serious limitation on what the reported scores can mean.

Second, unmatched inference budgets. Execution time rose from 56.1 s to 207.3 s per unit across the development path (Table 13), so architecture and computational expenditure covary, and one could argue that the extended configurations scored better simply because more computation was spent on them. Two measured features weigh against that reading. The number of executed procedural steps was nearly constant across conditions (11.2 to 13.1 per execution), so the growth in executor-side calls (12.5 to 23.5) reflects ensemble fan-out at five designated steps rather than more extended deliberation. And the measurement pipeline, not the architecture, accounts for 64 to 76 percent of estimated cost, with the multi-agent share inflated by the per-step procedural-validity regime. Most of the additional expenditure therefore arises from evaluation, which cannot improve the scores it produces. This does not eliminate the confound—inference budgets were not experimentally controlled—but it locates most of the growth outside the causal path in question.

Third, dependence between repeated executions. Each dilemma is shared by twenty-five executions within a condition. Mixed-effects models treating dilemma as a random intercept reproduced the paired-test significance pattern in all thirty comparisons (Section 4.2.2), so clustering within dilemmas does not account for the differences we report. That model does not, however, cover the further dependence introduced by reasoning-bank retrieval in the augmented condition, where later executions could retrieve earlier ones.

Fourth, overlap between generation and evaluation criteria. The indicators were operationalized by the same research team that designed the 20-step procedure, so the instrument and the object it measures were developed by the same research team, and an architecture that follows that procedure closely may be favored by design. No independent operationalization of advice quality was applied, and none of the four indicators was validated against an external criterion.

Fifth, non-equivalent measurement of procedural validity. As set out in Section 5.2.3, the single-agent condition was scored with all twenty steps in view and the multi-agent conditions one step at a time in isolation. We do not restate that argument here, but note its status among the confounds: it is specific to one indicator, yet it is the indicator carrying the study’s most conspicuous negative result, and it is the reason we treat that result as a finding about the instrument rather than about execution.

The first and fourth confounds share a structure. Both arise because the system that produces the advice, the instrument that scores it, and the criteria the instrument encodes originate together, and no arrangement of internal comparisons can break that circle. They are, in our view, the strongest argument for the independent expert validation identified as the highest-priority next step in the Limitations subsection below—not as an optional extension of this work, but as a condition for interpreting its scores as externally meaningful.

### 5.4. Limitations and Future Work

First, the study relied on LLM-as-a-judge measurement without direct assessment by human ethicists or domain experts. The high Cronbach’s alpha values show that the LLM-derived scores formed coherent measurement patterns, but they do not establish inter-rater reliability in the conventional human-evaluator sense, normative validity, or freedom from shared model bias, and the high mean scores suggest possible ceiling effects. Second, the empirical scope was limited to 20 dilemma cases and a predefined set of ethical principles and retrieval sources. Although the EPKB contains bilingual Korean- and English-language materials, this does not demonstrate that CREA accommodates culturally distinct moral traditions or language-dependent interpretations, given that moral priorities differ across societies, institutions, and professional communities (Nagenborg, 2007). Third, the study evaluated the quality of generated advice rather than its effect on human deliberation; whether CREA helps users identify neglected considerations and make better decisions, or instead induces automation bias and excessive deference, remains unknown. Fourth, the results depend on the specific LLMs, provider versions, prompts, retrieval resources, and settings used in 2025–2026, so reproducibility requires versioned prompts, archived knowledge sources, fixed evaluation sets, execution logs, and periodic re-evaluation. Fifth, several workflow constants—the hybrid-similarity weights and thresholds, the NAI ≥ 3.5 retrieval filter, and the 30-point adoption margin at Step 16—were fixed heuristically during pilot runs for stable branching and lack independent normative justification. We did not conduct a sensitivity analysis of their influence on the reported outcomes. In particular, the Step 16 margin determines whether the procedure terminates at seventeen or twenty steps and therefore affects the executed-step count over which procedural validity is computed, so its influence on the reported procedural-validity means is unquantified. Finally, the reasoning bank permits information leakage between executions. Retrieval selects stored executions whose dilemma-text similarity exceeds a threshold, without excluding the dilemma currently being solved; because each dilemma was executed twenty-five times per condition and every execution was written to the bank, later executions of a dilemma could retrieve earlier executions of that same dilemma, including their complete 20-step traces. The augmented condition is therefore not independent across repetitions of a dilemma, and its incremental gains over the multi-LLM condition should be interpreted in light of that dependence. A leakage-controlled replication—excluding same-dilemma entries at retrieval time—is required before the knowledge and reasoning banks can be credited with the improvement observed here.

Future work should proceed in three connected directions. The first is human-user and expert validation: controlled experiments comparing unaided decision-making, conventional LLM advice, and CREA-supported deliberation with ethicists, clinicians, public officials, and legal professionals in high-risk domains, alongside blinded expert panels benchmarking the LLM-based ratings. The second is stronger procedural control: monitoring the completeness and integrity of context transferred between agents, enforcing step-specific input–output contracts, and automatically returning the workflow to an earlier step when procedural drift is detected, evaluated specifically against the observed procedural-validity gap. The third is broader normative and technical generalization: multilingual and multicultural dilemma sets, dynamic weighting of external principles, more diverse EPKB/ERB resources, additional LLM families, extended self-improvement cycles, and cross-cultural expert review so that improved NAI scores reflect defensible normative alignment rather than merely greater agreement among computational evaluators.

## 6. Conclusions

This study designed, implemented, and evaluated CREA as a cognitively grounded artificial moral advisor that operationalizes CREM through a multi-LLM, multi-agent system. We separate what the evidence establishes, what it invites us to interpret, and what remains to be tested.

The study’s evidential claims are limited but concrete. Within a system-internal evaluation over 500 matched execution units per configuration, adding agents alone did not improve advice quality; multi-LLM deliberation improved every measured indicator relative to the multi-agent baseline; and knowledge- and reasoning-bank augmentation added further small but significant gains in consistency, justifiability, and procedural validity, though not in normative alignment—gains whose attribution to the banks awaits the leakage-controlled replication described in Section 5.4. Against the single-agent baseline, the full configuration scored higher on consistency, justifiability, and normative alignment, no significant difference was detected in the overall mean, and procedural-validity scores remained lower—a gap consistent with the difference in scoring regimes documented in the stored measurement records (multi-agent steps scored in isolation versus single-agent steps scored within the full 20-step context; Section 5.2.3, Table 17), although an execution-level contribution cannot be excluded.

What the study invites us to interpret is the relationship between these results and the cognitive account that motivated the architecture. The improvement in reason-giving and normative alignment under multi-model deliberation is consistent with the Rawlsian claim that plural weighing of competing conceptions strengthens justification, and the 20-step trace renders that weighing inspectable. This is an interpretation supported by the pattern of results, not a demonstration of moral validity: CREA produces auditable reasoning, not correct answers.

What remains to be tested is the claim that matters most for practice: whether such advice actually helps people reason better. Establishing this requires controlled studies with ethicists, clinicians, public officials, and legal professionals comparing unaided decision-making, conventional LLM advice, and CREA-supported deliberation, together with blinded expert panels benchmarking the LLM-derived ratings and cross-cultural dilemma sets. Until then, claims that CREA scaffolds human moral judgment, that it is suitable for deployment in high-risk professional domains, or that it can serve as a reference architecture for ethical decision-support systems should be read as design hypotheses rather than as findings of this study.

For researchers and practitioners building AI-supported ethical decision-support systems, three design lessons follow from the evidence. First, evaluation pipelines themselves require validation: a measurement pipeline built from the same models that generate the output may produce internally coherent scores with limited external interpretability, and its granularity can itself create apparent architectural differences—in this study, converging forensic evidence indicated that the single largest reported effect was substantially a property of the instrument (Section 5.2.3), although an execution-level contribution could not be excluded. Second, deliberative diversity, not agent count, was associated with the improvements we observed; the multi-LLM extension, not agent decomposition, was the component whose addition coincided with consistent gains across all measured indicators (Table 8), although inference budgets were not experimentally controlled (Section 5.3), and decomposition without diversity was associated with lower procedural-validity scores and no measured gain. Third, procedural control deserves first-class status: state schemas, mandatory step checks, and context-integrity validation should be treated as normative design components and evaluated with instruments that respect the procedure’s relational structure.

## Figures and Tables

**Table 1:** Five-stage structure and content of CCREM.

CCREM Stage	Theoretical Correspondence	Content
1. Original State of Equilibrium	Piaget: initial equilibrium/Rawls: original position	The preparatory condition for both procedures: a cognitively and ethically optimized starting state. It is dynamic rather than static, oriented toward a better equilibrium and in interaction with its environment.
2. Ethical assimilation	Piaget: assimilation/Rawls: narrow reflective equilibrium	A coherence judgment that determines whether the moral judgment held by the current cognitive schema and the ethical principles supplied by the environment support one another. Both source procedures share the method of consistency adjudication.
3. State of Disequilibrium	Piaget: disequilibrium/Rawls: (implicit) inconsistency or conflict	The state exposed when assimilation fails: perturbation, contradiction, or logical inconsistency. It supplies the motivation for the transition to accommodation, and thereby the connecting link between the two theories.
4. Ethical accommodation	Piaget: accommodation via reflective abstraction/Rawls: wide reflective equilibrium	Alternative ethical principles are examined and compared (differentiation and integration), supporting and opposing reasons are analyzed (relativization of concepts), and the weight of those reasons is assessed (quantification of relations), so that a principle is selected or the commitments are mutually adjusted.
5. Better State of Reflective Equilibrium	Piaget: better equilibrium/Rawls: reflective equilibrium	A state more advanced than the starting condition, in which coherence between moral judgment and ethical principles is secured and the revised principles have been justified.

**Table 2:** Four-Stage Structure and Functions of OCREM.

Stage	Steps	Function
1. Cognitive Processing	1–6	Compares and analyzes the initial moral judgment and ethical principles on the basis of objective information
2. Reflective Processing	7–15	Recognizes inconsistencies between judgments and principles and explores and compares alternative principles and cases
3. Equilibration (Ethical Decision)	16–17	Executes the optimal ethical judgment through weighted selection and adjustment of judgments
4. Ethical Implementation & Evaluation	18–20	Implements the justified decision and evaluates its outcomes

**Table 3:** From theoretical commitment to architectural decision.

Theoretical Commitment	Architectural Decision It Licenses	Where Implemented
CREM specifies four executable stages (Kim & Ahn, 2026)	Four stage-aligned agents, so the division of labor tracks the equilibration cycle rather than an arbitrary functional decomposition	Section 3.2
Assimilation requires ethical principles supplied by the environment, not recalled from within (Piaget, 1975/1985)	The EPKB supplies a curated, citable principle set retrieved at the assimilation step, instead of relying on principles recalled from model parameters	Section 3.3; Step 3
Disequilibrium is the engine of the procedure, not a failure mode (Piaget, 1975/1985)	An explicit conflict-detection step, with a branch that terminates the cycle early when no conflict is found rather than manufacturing one	Section 3.2; Steps 5–6
Accommodation proceeds by reflective abstraction: differentiation, relativization of concepts, quantification of relations (Piaget, 1975/1985)	The Reflective Agent generates alternative principles, produces counterarguments, and scores the relative weights of supporting and opposing reasons as three distinct operations rather than one judgment call	Section 3.2; Steps 7–15
Assimilation requires ethical principles supplied by the environment, not recalled from within (Piaget, 1975/1985), Wide reflective equilibrium requires weighing alternative conceptions and the comparative strength of the reasons for them (Rawls, 2017; Daniels, 1979)	The five-model ensemble is applied at the accommodation stage only, so alternatives are generated by independent models rather than by one model simulating plurality	Section 3.4; Steps 3, 11, 12, 13, 18
Development presupposes accumulated equilibration outcomes (Piaget, 1975/1985)	The ERB stores completed executions for retrieval. Qualification: retrieval is a static similarity lookup and no weights are updated, so this enables reuse rather than development	Section 3.3
Procedural consistency, not outcome acceptability, is what the theory constrains	Procedural validity is measured step by step rather than only at the final recommendation, and is reported as a separate indicator	Section 3.6

Note. Rows are ordered by the stage of the equilibration cycle to which they apply. The final row states a commitment about what the theory constrains, and therefore about what the evaluation must measure.

**Table 4:** Comparison of representative prior studies with CREA and implications for CREA.

Representative Prior Study	Evidence Type	Similarities and Differences with CREA	Implications for CREA
Yilmaz et al. (2017)	Conceptual proposal	Similarity: Implements coherence-driven reflective equilibrium as a computational model Difference: Confined to a single reasoning engine; no multi-agent collaboration structure distributing perspectives	The closest theoretical counterpart to CREM, sharing coherence-driven mutual adjustment between intuitions and principles, which CREA extends with Piagetian equilibration, a 20-step procedure, procedural validity, external principle verification, and multi-LLM evaluation.
Wallach et al. (2010)	Conceptual proposal	Similarity: Moral judgment integrating emotion and reason on a cognitive-process substrate Difference: A single-agent cognitive model; no deliberation among conflicting perspectives	Provides cognitive-architecture grounding for CREM’s cognition–reflection–equilibration–decision–action hierarchy, which CREA operationalizes through LLM agent roles, external principle retrieval, and memory/evaluation loops.
Conitzer et al. (2017)	Conceptual proposal	Similarity: Oriented toward general, formal ethical reasoning frameworks Difference: A single unified framework; no coordination mechanism for multi-perspective agents	Supports CREA’s multi-LLM adjudication based on reason comparison rather than voting, with separate measurement of justifiability, procedural validity, and normative alignment, complemented by step-level explanations ensuring transparent and verifiable reasoning.
Machado et al. (2024)	Computational evaluation		
Murukannaiah et al. (2020)	Conceptual proposal	Similarity: Defines ethics as an inherently multi-agent problem Difference: Stops at conceptual foundations for sociotechnical systems; no concrete deliberative mechanism	Provides a basis for theorizing CREA as an ethical MAS with role-specific responsibilities, mutual critique, and procedural legitimacy, reframing the Reasoning Bank as an institutional record of norms, cases, and accountability rather than simple memory.
Mashayekhi et al. (2022)	Computational evaluation	Similarity: System-level norm emergence and fairness assurance Difference: Centered on norm emergence and constraints; does not reach conclusions through coherence deliberation among principles	Extends the EPKB/ERB toward norm learning by recording stakeholder effects, violations, and long-term outcomes, while cautioning that multi-LLM consensus must not be equated with ethicality.
Stenseke (2024)	Computational evaluation	Similarity: Computational implementation of norm-based and virtue ethics Difference: Centered on implementing a single ethical theory rather than coherent adjustment of plural principles	Supports extending CREA’s equilibration into sustained moral learning via the Reasoning Bank, with multi-LLM agents representing and reconciling distinct ethical perspectives, evaluated in terms of behavioral consistency and the preservation of dissent rather than consensus alone.
Woodgate (2025)	Conceptual proposal		
Yamani et al. (2025)	Computational evaluation	Similarity: Role differentiation and structured debate in LLM multi-agent settings Difference: No theoretical grounding in coherence or reflective equilibrium; weak guarantees of judgment explainability	The closest recent multi-agent LLM comparison: MALEA addresses design-time ethics requirements while CREA covers runtime dilemma deliberation, empirically mitigating the variability and verification problems MALEA identified.

**Table 5:** Comparison of the four experimental architecture conditions.

Design Dimension	Single-Agent	Multi-Agent	Multi-LLM	Multi-LLM + KRB
Agent structure	Monolithic 20-step state graph	Six role-specialized agents	Six role-specialized agents	Six role-specialized agents
LLM plurality	1 primary LLM	1 primary LLM	one primary LLM + up to five auxiliary LLMs	one primary LLM + up to five auxiliary LLMs
Ethical Principles Knowledge Base (EPKB)	Not used	Not used	Not used	Principles + web + document RAG
Ethical Reasoning Bank (ERB)	Not used	Not used	Not used	Hybrid-similarity case retrieval (KRB only)
In-process LLM deliberation	Primary LLM only	Primary LLM only	5-LLM ensemble (Steps 3, 11, 12, 13, 18)	5-LLM ensemble (Steps 3, 11, 12, 13, 18)
Measurement (post hoc) scoring	5-LLM ensemble	5-LLM ensemble	5-LLM ensemble	5-LLM ensemble

Note. The multi-LLM ensemble was used in two distinct roles. In-process deliberation across multiple LLMs occurred only in the Multi-LLM and Multi-LLM + KRB conditions. Post hoc measurement scoring by the Measurement agent, in contrast, always queried all five LLMs in every condition—including Single-Agent; this is why inter-rater internal-consistency coefficients (Cronbach’s α) are reported for all four architectures.

**Table 6:** Descriptive summary of evaluation scores by architecture, presented as mean (standard deviation).

Indicator	Single	Multi	Multi-LLM	Multi-LLM + KRB
Consistency	4.683 (0.723)	4.630 (0.567)	4.745 (0.528)	4.806 (0.430)
Justifiability	4.626 (0.383)	4.658 (0.420)	4.788 (0.351)	4.832 (0.304)
Procedural validity	4.574 (0.284)	3.975 (0.318)	4.066 (0.319)	4.100 (0.311)
Normative alignment index (NAI)	4.105 (0.672)	4.076 (0.633)	4.207 (0.596)	4.238 (0.554)
Overall mean	4.497 (0.414)	4.335 (0.410)	4.452 (0.378)	4.494 (0.341)

Note. N = 500 matched execution units per architecture. Values are mean (SD) on a five-point scale.

**Table 7:** Coefficient of variation (%) by architecture.

Metric	Single	Multi	Multi-LLM	Multi-LLM + KRB
Consistency	15.43	12.25	11.12	8.95
Justifiability	8.27	9.01	7.33	6.28
Procedural validity	6.20	7.99	7.84	7.58
NAI	16.38	15.54	14.16	13.07
Overall mean	9.21	9.46	8.49	7.58

Note. Dispersion falls along the development path within the multi-agent family on every indicator (and monotonically across all four conditions for Consistency and NAI), so the extended configurations produced not only higher but also more stable scores.

**Table 8:** All six pairwise architecture comparisons (paired samples, df = 499).

Metric	Comparison (A vs. B)	Δ (B − A)	95% CI	t(499)	*p*	Holm *p*	dz	Wilcoxon *p*
Consistency	Single vs. Multi	−0.054	[−0.124, 0.017]	−1.494	.136	.150	−0.067	<.001
	Multi vs. Multi-LLM	+0.115	[0.055, 0.175]	3.755	<.001	<.001	0.168	<.001
	Multi-LLM vs. ML + KRB	+0.062	[0.013, 0.110]	2.505	.013	.038	0.112	.007
	Single vs. Multi-LLM	+0.062	[−0.006, 0.129]	1.783	.075	.150	0.080	.265
	Single vs. ML + KRB	+0.123	[0.063, 0.183]	4.054	<.001	<.001	0.181	<.001
	Multi vs. ML + KRB	+0.177	[0.125, 0.228]	6.763	<.001	<.001	0.302	<.001
Justifiability	Single vs. Multi	+0.032	[−0.008, 0.071]	1.582	.114	.114	0.071	.046
	Multi vs. Multi-LLM	+0.131	[0.092, 0.170]	6.548	<.001	<.001	0.293	<.001
	Multi-LLM vs. ML + KRB	+0.044	[0.015, 0.073]	2.946	.003	.007	0.132	.003
	Single vs. Multi-LLM	+0.162	[0.128, 0.197]	9.151	<.001	<.001	0.409	<.001
	Single vs. ML + KRB	+0.206	[0.174, 0.239]	12.497	<.001	<.001	0.559	<.001
	Multi vs. ML + KRB	+0.175	[0.137, 0.212]	9.189	<.001	<.001	0.411	<.001
Procedural validity	Single vs. Multi	−0.599	[−0.621, −0.578]	−54.438	<.001	<.001	−2.435	<.001
	Multi vs. Multi-LLM	+0.092	[0.073, 0.111]	9.470	<.001	<.001	0.424	<.001
	Multi-LLM vs. ML + KRB	+0.034	[0.015, 0.053]	3.517	<.001	<.001	0.157	.003
	Single vs. Multi-LLM	−0.508	[−0.530, −0.486]	−45.348	<.001	<.001	−2.028	<.001
	Single vs. ML + KRB	−0.474	[−0.495, −0.452]	−43.295	<.001	<.001	−1.936	<.001
	Multi vs. ML + KRB	+0.125	[0.106, 0.145]	12.697	<.001	<.001	0.568	<.001
NAI	Single vs. Multi	−0.028	[−0.085, 0.028]	−0.984	.325	.529	−0.044	.279
	Multi vs. Multi-LLM	+0.130	[0.076, 0.185]	4.742	<.001	<.001	0.212	<.001
	Multi-LLM vs. ML + KRB	+0.031	[−0.024, 0.086]	1.117	.264	.529	0.050	.354
	Single vs. Multi-LLM	+0.102	[0.045, 0.159]	3.530	<.001	.001	0.158	.003
	Single vs. ML + KRB	+0.133	[0.077, 0.190]	4.659	<.001	<.001	0.208	<.001
	Multi vs. ML + KRB	+0.162	[0.108, 0.215]	5.944	<.001	<.001	0.266	<.001
Overall mean	Single vs. Multi	−0.162	[−0.197, −0.128]	−9.316	<.001	<.001	−0.417	<.001
	Multi vs. Multi-LLM	+0.117	[0.084, 0.150]	6.974	<.001	<.001	0.312	<.001
	Multi-LLM vs. ML + KRB	+0.043	[0.013, 0.072]	2.838	.005	.014	0.127	.018
	Single vs. Multi-LLM	−0.045	[−0.078, −0.013]	−2.724	.007	.014	−0.122	<.001
	Single vs. ML + KRB	−0.003	[−0.033, 0.028]	−0.178	.859	.859	−0.008	.060
	Multi vs. ML + KRB	+0.160	[0.130, 0.190]	10.421	<.001	<.001	0.466	<.001

Note. Δ = B minus A over 500 matched execution units. Holm adjustment applied within each metric across the six comparisons. For the Consistency metric in Single vs. Multi, the significant Wilcoxon *p* reflects a ceiling effect (218 tied pairs at 5.0) with directional asymmetry among the non-tied pairs (177 vs. 105), not a substantively important difference.

**Table 9:** Linear mixed-effects robustness checks: β (*p*) for each contrast.

Metric	S → M β (*p*)	M → ML β (*p*)	ML → KRB β (*p*)	S → ML β (*p*)	S → KRB β (*p*)	M → KRB β (*p*)
Consistency	−0.054 (.141)	+0.115 (<.001)	+0.062 (.021)	+0.062 (.085)	+0.123 (<.001)	+0.177 (<.001)
Justifiability	+0.032 (.121)	+0.131 (<.001)	+0.044 (.010)	+0.162 (<.001)	+0.206 (<.001)	+0.175 (<.001)
Procedural validity	−0.599 (<.001)	+0.092 (<.001)	+0.034 (<.001)	−0.508 (<.001)	−0.474 (<.001)	+0.125 (<.001)
NAI	−0.028 (.351)	+0.130 (<.001)	+0.031 (.272)	+0.102 (<.001)	+0.133 (<.001)	+0.162 (<.001)
Overall mean	−0.162 (<.001)	+0.117 (<.001)	+0.043 (.008)	−0.045 (.011)	−0.003 (.869)	+0.160 (<.001)

Note. Model: metric~architecture + executor LLM + execution round (fixed effects), with dilemma case as a random intercept. N = 1000 observations per contrast (500 matched units in each condition). Between-dilemma variance ranged from 0.027 to 0.174; residual variance from 0.025 to 0.331. All thirty contrasts reproduce the significance pattern of the paired tests in Table 8.

**Table 10:** Internal consistency (Cronbach’s α) by architecture.

Scale	Items	Single	Multi	Multi-LLM	Multi-LLM + KRB
Consistency	5	0.969	0.906	0.958	0.936
Justifiability	5	0.867	0.825	0.887	0.873
Procedural validity	5	0.778	0.872	0.923	0.926
Overall 15-item measurement scale	15	0.919	0.929	0.947	0.943
Execution-level 4-indicator scale	4	0.750	0.838	0.833	0.845
Execution-level 3-indicator scale	3	0.670	0.834	0.810	0.824

Note. Alpha coefficients represent the internal consistency of measurement scores, not direct evidence of evaluator reliability or normative correctness.

**Table 11:** Adjacent architecture comparisons of Cronbach’s α. Δα is defined as B minus A.

Scale	Comparison	Δα (B − A)	95% Bootstrap CI	Perm. *p*	Result
Consistency	S vs. M	−0.063	[−0.101, −0.036]	<.001	Significant
	M vs. ML	0.051	[0.022, 0.089]	0.006	Significant
	ML vs. ML-KRB	−0.022	[−0.054, 0.008]	0.144	n.s.
Justifiability	S vs. M	−0.043	[−0.117, 0.013]	0.229	n.s.
	M vs. ML	0.062	[0.001, 0.136]	0.090	n.s.
	ML vs. ML-KRB	−0.014	[−0.052, 0.028]	0.600	n.s.
Procedural validity	S vs. M	0.094	[0.062, 0.126]	<.001	Significant
	M vs. ML	0.051	[0.031, 0.071]	<.001	Significant
	ML vs. ML-KRB	0.003	[−0.008, 0.014]	0.605	n.s.
Overall 15-item scale	S vs. M	0.010	[−0.006, 0.026]	0.313	n.s.
	M vs. ML	0.017	[0.005, 0.031]	0.014	Significant
	ML vs. ML-KRB	−0.004	[−0.012, 0.005]	0.433	n.s.
Exec. 4-indicator scale	S vs. M	0.087	[0.057, 0.120]	<.001	Significant
	M vs. ML	−0.004	[−0.033, 0.024]	0.752	n.s.
	ML vs. ML-KRB	0.011	[−0.016, 0.041]	0.453	n.s.
Exec. 3-indicator scale	S vs. M	0.164	[0.114, 0.217]	<.001	Significant
	M vs. ML	−0.024	[−0.055, 0.006]	0.132	n.s.
	ML vs. ML-KRB	0.014	[−0.018, 0.046]	0.396	n.s.

Note. Confidence intervals were estimated with paired bootstrap resampling; *p* values were estimated with paired permutation tests (3000 resamples). n.s. = not significant.

**Table 12:** Extended descriptive statistics by architecture, including median, minimum, and maximum.

Metric	Architecture	N	Mean	SD	Median	Min	Max
Consistency	Single	500	4.683	0.723	5.000	1.200	5.000
	Multi	500	4.630	0.567	4.800	1.800	5.000
	Multi-LLM	500	4.745	0.528	5.000	1.800	5.000
	Multi-LLM + KRB	500	4.806	0.430	5.000	1.800	5.000
Justifiability	Single	500	4.626	0.383	4.800	2.200	5.000
	Multi	500	4.658	0.420	4.800	1.800	5.000
	Multi-LLM	500	4.788	0.351	5.000	1.800	5.000
	Multi-LLM + KRB	500	4.832	0.304	5.000	3.000	5.000
Procedural validity	Single	500	4.574	0.284	4.588	3.718	5.000
	Multi	500	3.975	0.318	3.956	3.284	4.822
	Multi-LLM	500	4.066	0.319	4.088	3.266	4.844
	Multi-LLM + KRB	500	4.100	0.311	4.134	3.200	4.844
NAI	Single	500	4.105	0.672	4.270	1.860	5.000
	Multi	500	4.076	0.633	4.190	2.360	4.980
	Multi-LLM	500	4.207	0.596	4.390	2.370	4.980
	Multi-LLM + KRB	500	4.238	0.554	4.370	2.760	4.960
Overall mean	Single	500	4.497	0.414	4.606	2.927	4.989
	Multi	500	4.335	0.410	4.420	2.842	4.938
	Multi-LLM	500	4.452	0.378	4.589	2.964	4.920
	Multi-LLM + KRB	500	4.494	0.341	4.615	3.094	4.933

**Table 13:** Model calls, tokens, latency, and estimated cost by condition.

Condition	Execution Dates	Exec. Calls/Run	Meas. Calls/Run	Total Calls	Exec. Input Tok	Exec. Output Tok	Meas. Tokens	Latency s (vs. Single)	Cost $
Single-Agent	2026-04-20–04-27	13.07	80.35	46,709	15,265,057	1,962,621	24,250,987	56.1 (1.00×)	104.48
Multi-Agent	2026-04-25–05-30	12.50	150.93	81,715	20,022,906	2,717,971	66,631,329	72.5 (1.29×)	188.12
Multi-LLM	2026-05-02–05-18	23.54	152.40	87,971	21,086,434	3,074,001	73,041,111	119.1 (2.12×)	196.68
Multi-LLM + KRB	2026-05-04–05-09	22.07	160.19	91,132	25,356,503	3,746,483	85,392,022	207.3 (3.70×)	218.09

Note. N = 500 matched execution units per condition, recovered from 2140 stored execution records (duplicate retries removed, keeping the latest run per dilemma × executor × round). Executor calls = executed CREM steps plus, in the multi-LLM conditions, auxiliary sub-model calls (ensemble size × fan-out steps). Measurement calls = evaluator-LLM calls; procedural validity contributes 5 calls per execution in the single-agent condition and 5 per executed step in the multi-agent family. Token counts use the heuristic estimator max(words × 1.3, characters/4) because provider tokenizers were unavailable offline (approximately ±15%). Costs use the unit prices in effect on the execution dates (Table 14).

**Table 14:** Unit prices used for the cost estimates (USD per 1 M tokens).

Model	Input	Output	Price Basis, as of the Execution Dates (2026-04-20 to 2026-05-30)
GPT-4.1	2.00	8.00	Standard uncached API rate; cached input $0.50/M; batch discount excluded
Claude Haiku 4.5	1.00	5.00	Standard global rate; prompt caching and batch discounts excluded
Gemini 2.5 Flash	0.30	2.50	Stable rate; output price includes thinking tokens
Grok-3	3.00	15.00	Original Grok-3 rate. From 2026-05-15, the grok-3 model identifier was billed as Grok-4.3 ($1.25/$2.50); this affects 9 of 400 Grok executions
DeepSeek V3	0.27	1.10	Official list rate; input at cache-miss price (cache hit $0.07/M)

Note. Prices are those applicable on the execution dates recorded in the logs, not prices at the time of writing; four of the five were verified as unchanged as of August 2026. Rates are standard on-demand list prices with uncached or cache-miss input, and exclude batch discounts, prompt caching, tool and search charges, taxes, and reseller markups.

**Table 15:** Measurement calls per execution, by component.

Component	Single	Multi	Multi-LLM	Multi-LLM + KRB
Consistency (one bundled prompt × 5 evaluators)	5.0	5.0	5.0	5.0
Justifiability (one bundled prompt × 5 evaluators)	5.0	5.0	5.0	5.0
Procedural validity	5.0 (bundled)	62.3 (per step)	58.5 (per step)	55.9 (per step)
NAI relevance (5 × principle)	42.8	51.6	54.9	61.8
NAI alignment (5 × filtered principle)	22.5	27.1	29.0	32.5
Total measurement calls per execution	80.4	150.9	152.4	160.2
Executor calls per execution	13.1	12.5	23.5	22.1
Measurement share of estimated cost	64.2%	73.0%	74.7%	76.4%

**Table 16:** Summary of findings by research question.

Research Question	What the Evidence Shows	Supporting Tables/Figures
RQ1. How should a multi-LLM, multi-agent architecture be designed in order to implement CREM as a functioning artificial moral advisor?	CREM was realized as six agent modules under LangGraph orchestration: four stage-aligned reasoning agents, an orchestration agent, and a measurement agent. All four configurations executed the 20-step procedure to completion across 500 execution units each.	Section 4.1; Figure 1 and Figure 2
RQ2. Can a CREM-based AMA deliver advice that is consistent, justifiable, procedurally valid, and normatively aligned?	Under the system’s own measurement, all four configurations scored high on a five-point scale: consistency 4.63–4.81, justifiability 4.63–4.83, NAI 4.08–4.24, procedural validity 3.98–4.57. Distributions are compressed near the maximum on consistency and justification, so ceiling effects apply. These are self-evaluated scores and do not establish normative validity.	Section 4.2.1; Table 6 and Table 7; Figure 3 and Figure 4
RQ3. How does advice quality vary across architectural configurations?	Agent decomposition alone produced no significant gain on any indicator and a large procedural-validity decline. Multi-LLM deliberation improved all five outcomes over the multi-agent baseline (dz = 0.168–0.424). KRB added small further gains (dz = 0.112–0.157) except on NAI. Against single-agent, the full configuration was higher on consistency, justifiability, and NAI, lower on procedural validity, and not significantly different on the overall mean.	Section 4.2.2; Table 8; Figure 5 and Figure 6

**Table 17:** Signatures of the two procedural-validity scoring regimes in the stored evaluation records.

Indicator	Single	Multi	Multi-LLM	Multi-LLM + KRB
Mean length of a procedural-validity reason (characters)	167.0	433.1	437.7	438.0
Median length	151	426	430	430
Reasons empty after parsing	1.3%	0.0%	0.0%	0.0%
Reasons naming a step (“Step N” or “AN”)	0.8%	55.1%	57.6%	58.1%
Per-step “executed” flag written to the record	present	absent	absent	absent
Procedural-validity calls per execution	5	5 × executed steps	5 × executed steps	5 × executed steps

Note. Computed over all stored procedural-validity reasons (42,035 single-agent and 88,345 multi-agent). Standalone per-step replies are longer than items parsed from one bundled twenty-item reply, never fail extraction, and restate the step label; a bundled reply carries the label outside the reason text. The per-step “executed” flag appears only in single-agent records, indicating a different serialization and therefore a different code path at the time of execution.

## Data Availability

The original data presented in the study are included in the article and its Supplementary Materials; further inquiries can be directed to the corresponding author.

## Supplementary Materials

The following supporting information can be downloaded at: https://www.mdpi.com/article/10.3390/jintelligence14090212/s1. Table S1: 20-dilemma_cases; Table S2: Four_architecture_analysis_total_data; Table S3: Mutil-LLM_calls_tokens_latency_cost_total_data. Document S1: AI-CREA_Technical_Documentation; Document S2: Four_Architecture_Statistics_Python_Sourcecode; Document S3: Full_pairwise_analysis_Python_Sourcecode; Document S4: MultiLLM_latency_cost_analysis_Python_Sourcecode. Dataset S1: Four_Architectures_ReasonBank_Datasets; Dataset S2: Four_Architectures_Measurement_Rawdata_Datasets.

## Abbreviations

The following abbreviations are used in this manuscript:

A2A	Agent-to-Agent (protocol)
AMA	Artificial Moral Advisor
BM25	Best Matching 25 (lexical ranking function)
CI	Confidence Interval
CREA	Cognitive–Reflective Equilibration Architecture
CREM	Cognitive–Reflective Equilibration Model
CSV	Comma-Separated Values
*d_z_*	Cohen’s dz (matched-samples effect size)
EPKB	Ethical Principles Knowledge Base
ERB	Ethical Reasoning Bank
JSON	JavaScript Object Notation
KRB	Knowledge/Reasoning Bank
LLM	Large Language Model
MCP	Model Context Protocol
NAI	Normative Alignment Index
OCREM	Operational Cognitive–Reflective Equilibration Model
RAG	Retrieval-Augmented Generation
RQ	Research Question
SD	Standard Deviation
SSE	Server-Sent Events

## Appendix A. OCREM Requests and Moral Dilemma Cases

**Table A1:** Twenty-Step OCREM Procedural Requests.

Stages	Procedural Requests
Stage 1 Cognitive Processing	The text of the ethical dilemma or judgment case is inserted at the head of the prompt, and the model is directed to work through the numbered requests in order.
	1. Identify and explain the objective elements of the case: the stakeholders involved, the established facts, and the ethical considerations at stake.
	2. State an intuitive moral judgment about the case, with its grounds, building on the Step 1 analysis.
	3. Drawing on the Step 1 analysis, survey a range of ethical principles or ethical judgment cases that bear on the dilemma.
	4. Set the Step 2 intuition against the principles and cases collected in Step 3 and characterize how they relate.
	5. Classify that relation as fully mutually supportive, partially inconsistent, or completely inconsistent.
	6. Where full mutual support holds, give the rationale, adopt the supported judgment as the final decision, write a decision statement, and jump to Step 18.
Stage 2 Reflective Processing	7. For each partial or complete inconsistency found in Step 5, articulate its underlying reason.
	8. Assess whether the conflict can be resolved by selecting among, or mutually adjusting, the intuitive judgment, the principles, and the cases.
	9. If resolution is judged feasible, justify this and proceed directly to Step 11 (Step 10 is skipped); if infeasible, proceed to Step 10.
	10. Where resolution is infeasible, explain why, forecast and appraise the consequences of the impasse, and terminate the procedure.
	11. Sketch alternative ethical principles or judgment cases beyond those examined in Step 3.
	12. Lay out the considerations for and against the Step 2 intuition, and for and against the Step 3 principles and cases.
	13. Do the same for each alternative introduced in Step 11.
	14. Consolidate Steps 12–13 into the intuitive judgment and its competing judgments; for every competitor, tally supporting and opposing opinions drawn from external information (statistics, expert views, etc.) and convert the tallies into percentage support and opposition ratios.
	15. Justify the scores assigned in Step 14 by citing the external evidence used.
Stage 3 Equilibration (Ethical Judgment)	16. If one or more competing judgments lead the opposition by 30 points or more in support score, select one of them or adjust among them to determine the optimal ethical judgment, write a decision statement, and jump to Step 18; otherwise continue to Step 17.
	17. Where no judgment clears the threshold, draft a decision statement that coordinates the competing judgments summarized in Step 14.
Stage 4 Ethical Implementation and Evaluation	18. Compare the initial intuition with the final decision reached through Steps 1–17, and state whether consistency has been preserved and justification achieved through the refined ethical principles.
	19. Project the outcomes to be expected if the final decision were enacted.
	20. Appraise whether the outcomes projected in Step 19 would contribute to resolving the dilemma originally posed.

Note. Table A1. Functional summary of the 20 OCREM procedural requests, paraphrased from the request texts first published in Kim and Ahn (2026; https://doi.org/10.3390/systems14070881). In CREA these requests are operationalized as step-specific prompts within the implementation described in Document S1.

**Table A2:** Twenty Moral Dilemma Cases.

Category	No.	Field	Moral Dilemma Cases
Human	1	Medical	How a physician should proceed when financial hardship leads a patient to decline treatment they need
	2	Environment	Weighing zero-packaging eco-friendly goods against plastic-wrapped goods that keep products fresher
	3	Counseling	What a social worker should do when a domestic-violence victim asks that the abuse be kept confidential
	4	Social Welfare	How to triage several urgent aid requests when welfare resources cannot cover them all
	5	School	Whether, and in what way, to step in upon witnessing a friend being bullied
	6	War	A wartime situation in which an infant’s crying threatens to reveal hiding villagers to searching enemy troops
	7	Rescue	Whether an already-full lifeboat should take aboard additional survivors
	8	Finance	How a financial professional should act when a client’s interests collide with the firm’s
	9	Construction	What a site supervisor should do on discovering safety hazards at a construction site
	10	Workplace	Whether to disclose product defects or to honor an obligation of corporate confidentiality
AI	11	AI Recruitment	Whether an AI hiring system shown to be biased should remain in use
	12	AI Speaker	Trading off the emergency-response capability of AI speakers against personal privacy
	13	Facial Recognition CCTV	Crime-preventive facial-recognition CCTV versus the stigmatization of former offenders
	14	Police Robot	The ethics of deploying police robots in view of their malfunction risks
	15	Deepfake	Therapeutic uses of deepfake technology to recreate deceased individuals versus its potential for abuse
	16	Cleaning Robot	Adopting cleaning robots at the cost of employment opportunities for older workers
	17	Drone Delivery	Job losses among delivery workers that a drone-delivery rollout may cause
	18	AI Weapons	The defense benefits of AI-weapons development weighed against their malfunction risks
	19	AI-Generated Images	Whether AI-generated artworks should be eligible for exhibition prizes
	20	Care Robot	Effects of emotional care robots on the parent–child relationship

Note. Table A2. The 20 moral dilemma cases (ten from human-society and ten from AI-technology contexts), restated in condensed form. The cases were first reported in Kim and Ahn (2026; https://doi.org/10.3390/systems14070881); the full case texts are given in Table S1.

## Appendix B. Technical Implementation Details

### Appendix B.1. Software Stack and Infrastructure

CREA comprises a single-page client interface communicating with a Flask-based application server orchestrating a LangGraph workflow engine. The backend was written in Python (3.12+), with LangChain as a unified abstraction layer over five LLM providers (OpenAI GPT-4.1, Anthropic Claude Haiku 4.5, Google Gemini 2.5 Flash, xAI Grok-3, DeepSeek V3). Pydantic enforced type-safe agent-state and inter-agent message contracts; Server-Sent Events streamed step-level progress to the client. All LLMs were configured with temperature = 0.0 and extended-thinking/reasoning tokens disabled. Infrastructure provisions included thread-local state isolation for concurrent requests, ThreadPoolExecutor-based parallel querying for ensembles, and atomic (temp-file-then-rename) JSON writes.

### Appendix B.2. Workflow Branching

The Orchestration agent executes state transitions and evaluates three branch conditions: a consistency check at Step 5 (routing to a final decision statement or to reflective analysis), an adjustment-feasibility check at Step 8 (routing to alternative exploration or to prediction/self-evaluation), and a 30-point threshold rule at Step 16 (routing to evaluation or to a further mutual-adjustment step).

### Appendix B.3. Retrieval Parameters

Both knowledge components use a hybrid retrieval score (0.6 × embedding cosine similarity + 0.4 × normalized BM25) computed over paragraph-boundary-aware chunks (500 characters, 50-character overlap), with Jaccard token overlap as a fallback when embeddings are unavailable. EPKB chunks are retrieved at a threshold of ≥0.45; ERB cases at ≥0.60 with top-k = 3, restricted to prior multi-agent runs with NAI ≥ 3.5. EPKB sources include the Stanford Encyclopedia of Philosophy and the Internet Encyclopedia of Philosophy (BM25-filtered) and document-level RAG over user-supplied files. Each ERB record stores the dilemma, 20-step trace, evaluation scores, and metadata as an individual JSON file. Pre-enrichment retrieval occurs at Steps 3, 11, 12, 13, and 18; retrieved material appears in 1.21 (EPKB) and 1.13 (ERB) steps per execution on average and, being a similarity lookup rather than a model call, incurs no additional model-token cost.

### Appendix B.4. Measurement Aggregation

Consistency and Justifiability are 5-point Likert ratings from the five-LLM panel. Procedural Validity is scored for each of the 20 steps individually by all five LLMs and aggregated as the mean, standard deviation, sum, and coefficient of variation. The NAI weights Ethical Relevance and Normative Alignment per principle.

## Appendix C. Measurement Agent—Evaluation Metric Definitions

Evaluation Prompts and Computation Formulas for the Four Metrics of the CREM-Based Ethical Decision-Making System.

### Appendix C.1. Overview of Evaluation Metrics

The Measurement Agent quantitatively evaluates the output of the CREM-based ethical decision-making process on four metrics. Each metric is measured in parallel by the full set of five LLMs and aggregated into a representative value.

**Table A3:** Overview of Evaluation Metrics.

Metric	Measurement Target	Scale	Representative Value
Consistency	Integrated data from Steps 18–20 (initial judgment ↔ final decision)	1–5 points	mean
Justifiability	Integrated data from Steps 18–20 (justification by the enhanced principle)	1–5 points	mean
Procedural Validity	Each stage of the 20-step CREM process (A1–A20)	1–5 points	mean of stage means
NAI (Normative Alignment Index)	Final decision ↔ ethical-principle alignment	Relevance 0–5 pts Alignment 1–5 pts	GAI

**Table A4:** Per-Metric Evaluation Query Prompts and Formulas.

Metric	Evaluation Query (LLM Prompt)	Response Format	Formula
Consistency	Across the outcomes derived from the CREM-based ethical decision-making procedure, compare the intuitive moral judgment of the ethical dilemma with the final ethical decision and assess whether logical consistency has been established. Target: integrated data from Steps 18–20 Scale: 1 (strongly disagree)–5 (strongly agree)	score = N, reason ≤ 3 sentences	Statistics computed after parallel measurement by 5 LLMs: mean = (Σ s_i_)/n sum = Σ s_i_ std = stdev(s_i_) CV = std/mean × 100 (%) (n = 5, s_i_ = score of the i-th LLM)
Justifiability	Across the outcomes derived from the CREM-based ethical decision-making procedure, compare the intuitive moral judgment of the ethical dilemma with the final ethical decision and assess whether justification has been achieved through an enhanced ethical principle, thereby establishing justifiability. Target: integrated data from Steps 18–20 Scale: 1–5 points	score = N, reason ≤ 3 sentences	Statistics computed after parallel measurement by 5 LLMs: mean = (Σ s_i_)/n sum = Σ s_i_ std = stdev(s_i_) CV = std/mean × 100 (%)
Procedural Validity	The following is the execution result of the 20-step CREM-based ethical decision-making process. Assign a 5-point-scale score only to the executed steps and provide the reason in three sentences or fewer. For N/A (non-executed) stages, respond with score = N/A. Target: all 20 CREM steps (A1–A20) evaluated in a single prompt Non-executed steps are excluded from the statistics	A1: score = N, reason = … … A20: score = N, reason = …	- Stage mean x^−^_k_ = (Σ s_ki_)/n (mean of 5 LLMs) - Representative value mean = (Σ x^−^_k_)/m (m = number of executed stages, k ∈ executed stages) sum = Σ x^−^_k_ std = stdev(x^−^_k_) CV = std/mean × 100 (%)

### Appendix C.2. NAI (Normative Alignment Index)—Detailed Process

The NAI measures the degree of alignment between the final decision of CREM Steps 6·16·17 and the ethical principles. After extracting ethical principles/cases (up to 15) in Steps 3·11, measurement is carried out by the five LLMs in two stages.

**Table A5:** NAI (Normative Alignment Index) Evaluation Query and Formula.

Stage	Evaluation Query (LLM Prompt)	Response Format	Formula
Step 1 Ethical Relevance	Rate the relevance of the corresponding ethical principle/case to the given dilemma and final ethical decision on a 0–5 scale, and briefly explain your reasoning. (0: not relevant at all–5: extremely relevant) Each principle measured by 5 LLMs Filter: top 50% by descending R_i_ AND R_i_ ≥ 3.5	score = N, reason = …	Relevance per principle R_i_ = (Σ r_l_)/5 (r_l_ = 0–5 score of LLM ℓ)
Step 2 Normative Alignment	Rate the normative alignment between the final decision and the corresponding ethical principle/case on three dimensions, each on a 1–5 scale: 1. Consistency: the principle and final decision align without contradiction 2. Justifiability: the principle contributes to justifying the decision as its basis 3. Priority: principles align without conflict	consistency = N, justifiability = N, priority = N, reason = …	Alignment per LLM aℓ = (C + J + P)/3 Alignment per principle A_i_ = (Σ aℓ)/5 Reliability: Cronbach’s α (when ≥ 2 principles)
Final Aggregate (GAI)	Using the relevance (R_i_) of the filtered principles as weights, compute a weighted average of the alignment (A_i_) to derive the overall alignment index.	—	GAI = Σ(A_i_ × R_i_)/Σ R_i_ Representative mean = GAI std, sum, CV computed in parallel

Note. std = sample standard deviation (stdev; when ≥2 scores, 0 if a single score). CV (coefficient of variation) = std ÷ mean × 100 (%)—the smaller the value, the higher the inter-LLM agreement. Cronbach’s α = [k/(k − 1)] × [1 − Σσ^2^_i_/σ^2^_total], k = number of principles—inter-rater (LLM) reliability.
