# Supplementary material for: Implementing a Cognitively Grounded Artificial Moral Advisor: A Multi-LLM Multi-Agent Approach Based on the Cognitive–Reflective Equilibration Model
Source: J Intell. 2026 Sep 2;14(9):212. doi: 10.3390/jintelligence14090212 (PMC13608209; doi:10.3390/jintelligence14090212)
Supplement: Supplementary file 1 [file jintelligence-14-00212-s001.zip › Document_S2.Four_Architecture_Statistics_Python_Sourcecode.pdf]

Document_S1.Four_Architecture_Statistics_Python_Sourcecode

```
#!/usr/bin/env python3
# -*- coding: utf-8 -*-
"""
=======================================================================
==============
CREA Four-Architecture Statistical Analysis
=======================================================================
==============
Reproducible analysis source for the manuscript:

  "Implementing a Cognitively Grounded Artificial Moral Advisor:
   A Multi-LLM Multi-Agent Approach Based on the
   Cognitive-Reflective Equilibration Model"
  (MDPI Journal of Intelligence, jintelligence-4460888)

This script reproduces, from the raw data files, every statistic reported in
the four-architecture comparison of the Results section:

  1. Data validation and execution-level score reconstruction
     - Consistency / Justification / Procedural validity are re-aggregated
       directly from the evaluator-level raw scores (5 LLM evaluators per
       execution unit).
     - NAI is taken from the NAI source files, with an optional correction
       table applied (Multi-LLM / GPT / R2, 20 values; see NAI_CORRECTIONS).
  2. Descriptive statistics by architecture (Table: Descriptive)
  3. Omnibus Friedman tests across the four matched architectures
  4. All six pairwise architecture comparisons per metric:
     paired t-test, 95% CI, Cohen's dz, Wilcoxon signed-rank test,
     Holm correction within metric (Table: Pairwise)
  5. Linear mixed-effects robustness checks
     (fixed: architecture, executor LLM, round; random intercept: dilemma)
  6. Cronbach's alpha internal-consistency analysis
     (metric-specific 5-item, 15-item, execution-level 4-/3-indicator scales)
  7. Alpha-difference tests (paired bootstrap CIs + paired permutation p)
  8. Manuscript figures: mean profiles, overall means with 95% CIs,
     alpha by architecture, box plots (overall and metric-wise)
  9. Export of all tables to a single Excel workbook
```

```
Requirements:
  python >= 3.10, pandas, numpy, scipy, statsmodels, matplotlib, openpyxl

Usage:
  python crea_statistical_analysis.py
  (adjust the CONFIG block below to point at the raw data files)

Notes on interpretation:
  Cronbach's alpha is computed as the internal consistency of measurement
  scores (the five LLM-derived ratings of the same execution unit treated
  as items; form-equivalent to a standardized agreement index closely
  related to ICC(2,k)).  It is NOT interpreted as conventional human
  inter-rater reliability or as evidence of normative validity.
================================================================
===============
"""

import warnings
import numpy as np
import pandas as pd
from scipy import stats

warnings.filterwarnings("ignore")

#
================================================================
=============
# CONFIG
#
================================================================
=============

RAW_FILES = {
    # comparison set : (evaluator-level file, NAI file, execution-total file)
    "01": ("01_S_MAgent_Cons_Just_PV.xlsx", "01_S_MAgent_NAI.xlsx", "01_S_MAgent_Tot.xlsx"),
    "02": ("02_MMLLM_Cons_Just_PV.xlsx",    "02_MMLLM_NAI.xlsx",    "02_MMLLM_Tot.xlsx"),
    "03":        ("03_MLLMMLLMKRB_Cons_Just_PV.xlsx",        "03_MLLMMLLMKRB_NAI.xlsx",
"03_MLLMMLLMKRB_Tot.xlsx"),
```

```
}

# Canonical source of each architecture (set, architecture label in file)
ARCH_SOURCE = {
    "Single":          ("01", "Single"),
    "Multi":           ("01", "Multi"),
    "Multi-LLM":       ("02", "Multi-LLM"),
    "Multi-LLM-KRB": ("03", "Multi-LLM-KRB"),
}
ARCHS = list(ARCH_SOURCE)

# Apply the documented NAI corrections for Multi-LLM / GPT / R2?
APPLY_NAI_CORRECTIONS = True

# NAI corrections (architecture Multi-LLM, executor GPT, round R2).
# Values taken from the corrected NAI source file; documented in the
# data-validation report (v3), Table C.
NAI_CORRECTIONS = {
    "1. Human_Medical": 4.22,   "2. Human_Environment": 3.84,
    "3. Human_Counseling": 4.42, "4. Human_Social Welfare": 3.88,
    "5. Human_School": 4.18,     "6. Human_War": 3.44,
    "7. Human_Rescue": 4.18,     "8. Human_Finance": 4.67,
    "9. Human_Construction": 4.67, "10. Human_Job": 4.44,
    "11. AI_AI Recruitment": 4.38, "12. AI_AI Speaker": 4.02,
    "13. AI_Face Recognition CCTV": 4.16, "14. AI_Police Robot": 4.09,
    "15. AI_Deepfake": 4.04,     "16. AI_Cleaning Robot": 3.94,
    "17. AI_Drone Delivery": 4.20, "18. AI_AI Weapon": 4.11,
    "19. AI_AI Generated Image": 3.82, "20. AI_Care Robot": 3.80,
}

SEED = 20260805              # RNG seed for bootstrap / permutation resampling
N_RESAMPLES = 3000           # resamples for alpha-difference tests
OUTPUT_XLSX = "CREA_four_architecture_analysis_output.xlsx"
MAKE_FIGURES = True

KEY = ["Dilemma", "ExLLM", "Round"]              # matched execution-unit index
METRICS = ["Cons", "Just", "PV", "NAI", "Overall"]
MNAMES = {"Cons": "Consistency", "Just": "Justification",
          "PV": "Procedural validity", "NAI": "NAI", "Overall": "Overall mean"}
```

```
# All six pairwise comparisons (A, B); differences are B - A
PAIRS = [("Single", "Multi"), ("Multi", "Multi-LLM"),
         ("Multi-LLM", "Multi-LLM-KRB"), ("Single", "Multi-LLM"),
         ("Single", "Multi-LLM-KRB"), ("Multi", "Multi-LLM-KRB")]
# Adjacent development-stage pairs (used for the alpha-difference table)
ADJACENT_PAIRS = PAIRS[:3]

rng = np.random.default_rng(SEED)

#
==========================================================================
=============
# 1. LOADING AND DATA VALIDATION
#
==========================================================================
=============

def load_evaluator(path: str) -> pd.DataFrame:
    """Evaluator-level file: 5 LLM evaluators x 500 execution units x 2 archs."""
    df = pd.ExcelFile(path).parse(0)
    df.columns = ["Dilemma", "Arch", "ExLLM", "Round", "Evaluator",
                  "Cons", "Just", "PV"]
    return df

def load_nai(path: str) -> pd.DataFrame:
    df = pd.ExcelFile(path).parse(0)
    df.columns = ["Dilemma", "Arch", "ExLLM", "Round", "NAI"]
    return df

def load_total(path: str) -> pd.DataFrame:
    """Execution-level compiled file (used for validation and latency only)."""
    df = pd.ExcelFile(path).parse(0)
    df.columns = ["No", "DateTime", "Arch", "Dilemma", "ExLLM", "Round",
                  "Cons", "Just", "PV", "NAI", "Elapsed"]
    return df

def load_all():
    ev = {k: load_evaluator(v[0]) for k, v in RAW_FILES.items()}
```

```
    na = {k: load_nai(v[1]) for k, v in RAW_FILES.items()}
    to = {k: load_total(v[2]) for k, v in RAW_FILES.items()}
    return ev, na, to

def validate_duplicate_sources(ev, na) -> list:
    """Architectures appearing in two files must be identical in both."""
    report = []
    dup = [("Multi", "01", "02"), ("Multi-LLM", "02", "03")]
    for arch, s1, s2 in dup:
        a = (ev[s1][ev[s1].Arch == arch].groupby(KEY)[["Cons", "Just", "PV"]]
             .mean().sort_index())
        b = (ev[s2][ev[s2].Arch == arch].groupby(KEY)[["Cons", "Just", "PV"]]
             .mean().sort_index())
        report.append((f"{arch} evaluator scores ({s1} vs {s2})",
                       float((a - b).abs().max().max())))
        an = na[s1].query("Arch==@arch").set_index(KEY)["NAI"].sort_index()
        bn = na[s2].query("Arch==@arch").set_index(KEY)["NAI"].sort_index()
        report.append((f"{arch} NAI ({s1} vs {s2})", float((an - bn).abs().max())))
    return report

def apply_nai_corrections(nai_df: pd.DataFrame) -> tuple[pd.DataFrame, int]:
    """Replace the 20 documented Multi-LLM / GPT / R2 NAI values."""
    df = nai_df.copy()
    mask = (df.ExLLM == "GPT") & (df.Round == "R2")
    n = 0
    for i in df[mask].index:
        d = df.at[i, "Dilemma"]
        if d in NAI_CORRECTIONS:
            df.at[i, "NAI"] = NAI_CORRECTIONS[d]
            n += 1
    return df, n

def build_execution_level(ev, na) -> dict[str, pd.DataFrame]:
    """One row per matched execution unit; metrics re-aggregated from raw."""
    data = {}
    for arch, (src, label) in ARCH_SOURCE.items():
        e = (ev[src][ev[src].Arch == label]
             .groupby(KEY)[["Cons", "Just", "PV"]].mean())
        n = na[src].query("Arch==@label")
```

```
        if arch == "Multi-LLM" and APPLY_NAI_CORRECTIONS:
            n, applied = apply_nai_corrections(n)
            print(f"   NAI corrections applied to Multi-LLM: {applied} rows")
        d = e.join(n.set_index(KEY)["NAI"]).reset_index()
        d["Overall"] = d[["Cons", "Just", "PV", "NAI"]].mean(axis=1)
        data[arch] = d.set_index(KEY).sort_index()
        assert len(data[arch]) == 500, f"{arch}: expected 500 execution units"
    idx = data["Single"].index
    for arch in ARCHS:
        assert data[arch].index.equals(idx), f"{arch}: index mismatch"
    return data

#
==================================================================
=============
# 2. DESCRIPTIVE STATISTICS
#
==================================================================
=============

def descriptives(data) -> pd.DataFrame:
    rows = []
    for arch in ARCHS:
        for m in METRICS:
            x = data[arch][m]
            rows.append({
                "Metric": MNAMES[m], "Architecture": arch, "N": len(x),
                "Mean": x.mean(), "SD": x.std(ddof=1),
                "Variance": x.var(ddof=1),
                "CV (%)": 100 * x.std(ddof=1) / x.mean(),
                "Median": x.median(), "Min": x.min(), "Max": x.max(),
            })
    return pd.DataFrame(rows)

#
==================================================================
=============
# 3. OMNIBUS FRIEDMAN TESTS
#
```

```
==============================================================
=============

def friedman_tests(data) -> pd.DataFrame:
    """Friedman chi-square over the 4 matched architectures, per metric."""
    rows = []
    for m in METRICS:
        cols = [data[a][m].values for a in ARCHS]
        chi2, p = stats.friedmanchisquare(*cols)
        rows.append({"Metric": MNAMES[m], "Test": "Friedman chi-square",
                     "chi2": chi2, "df": len(ARCHS) - 1, "p": p})
    return pd.DataFrame(rows)

#
==============================================================
=============
# 4. PAIRWISE COMPARISONS (paired t, Wilcoxon, Cohen's dz, Holm)
#
==============================================================
=============

def holm_adjust(pvals: list[float]) -> np.ndarray:
    """Holm step-down adjustment with monotonicity enforcement."""
    p = np.asarray(pvals, dtype=float)
    order = np.argsort(p)
    m = len(p)
    adj = np.empty(m)
    running_max = 0.0
    for rank, i in enumerate(order):
        val = min(1.0, (m - rank) * p[i])
        running_max = max(running_max, val)
        adj[i] = running_max
    return adj

def paired_comparison(x: pd.Series, y: pd.Series) -> dict:
    """Paired t-test with 95% CI, Cohen's dz, Wilcoxon signed-rank."""
    d = (y - x).values
    n = len(d)
    md, sd = d.mean(), d.std(ddof=1)
```

```
    se = sd / np.sqrt(n)
    tval = md / se
    p = 2 * stats.t.sf(abs(tval), n - 1)
    half = stats.t.ppf(0.975, n - 1) * se
    try:
        wp = stats.wilcoxon(y.values, x.values).pvalue
    except ValueError:          # all differences zero
        wp = np.nan
    return dict(A_mean=x.mean(), B_mean=y.mean(), diff=md,
                ci_low=md - half, ci_high=md + half,
                t=tval, df=n - 1, p=p, dz=md / sd, wilcoxon_p=wp)

def pairwise_table(data) -> pd.DataFrame:
    rows = []
    for m in METRICS:
        res = [paired_comparison(data[a][m], data[b][m]) for a, b in PAIRS]
        adj = holm_adjust([r["p"] for r in res])
        for (a, b), r, hp in zip(PAIRS, res, adj):
            rows.append({"Metric": MNAMES[m], "A": a, "B": b, **r,
                         "holm_p": hp})
    return pd.DataFrame(rows)

#
============================================================
=============
# 5. LINEAR MIXED-EFFECTS ROBUSTNESS CHECKS
#
============================================================
=============

def lmm_table(data) -> pd.DataFrame:
    """metric ~ architecture + executor LLM + round, random intercept: dilemma.

    Executor LLM and round are modeled as fixed effects because each has
    only five levels, below the conventional threshold for stable
    random-effects variance estimation.
    """
    import statsmodels.formula.api as smf
    rows = []
```

```
    for a, b in PAIRS:
        dd = pd.concat([data[a].reset_index().assign(Arch=a),
                        data[b].reset_index().assign(Arch=b)])
        dd["Arch"] = pd.Categorical(dd["Arch"], categories=[a, b])
        for m in METRICS:
            fit = smf.mixedlm(f"{m} ~ C(Arch) + C(ExLLM) + C(Round)",
                              dd, groups=dd["Dilemma"]).fit(reml=True)
            term = [c for c in fit.params.index if c.startswith("C(Arch)")][0]
            rows.append({"A": a, "B": b, "Metric": MNAMES[m],
                         "beta": fit.params[term], "SE": fit.bse[term],
                         "z": fit.params[term] / fit.bse[term],
                         "p": fit.pvalues[term],
                         "Var_dilemma": float(fit.cov_re.iloc[0, 0]),
                         "sigma2_resid": float(fit.scale)})
    return pd.DataFrame(rows)

#
============================================================================
# 6. CRONBACH'S ALPHA
#
============================================================================

def cronbach_alpha(mat: np.ndarray) -> float:
    """mat: observations x items."""
    k = mat.shape[1]
    item_var = mat.var(axis=0, ddof=1).sum()
    total_var = mat.sum(axis=1).var(ddof=1)
    return k / (k - 1) * (1 - item_var / total_var)

def evaluator_wide(ev, arch) -> dict[str, pd.DataFrame]:
    """500 x 5 matrices (execution units x evaluators) per metric,
    plus the concatenated 15-item matrix."""
    src, label = ARCH_SOURCE[arch]
    d = ev[src][ev[src].Arch == label]
    out = {}
    for m in ["Cons", "Just", "PV"]:
        out[m] = (d.pivot_table(index=KEY, columns="Evaluator", values=m)
```

```
                    .sort_index())
    out["15"] = pd.concat([out["Cons"], out["Just"], out["PV"]], axis=1)
    return out

SCALES = ["Cons", "Just", "PV", "15", "exec4", "exec3"]
SCALE_NAMES = {
    "Cons": "Consistency (5 items)", "Just": "Justification (5 items)",
    "PV": "Procedural validity (5 items)", "15": "15-item measurement scale",
    "exec4": "Execution-level 4-indicator scale",
    "exec3": "Execution-level 3-indicator scale (excl. NAI)",
}

def scale_matrix(wide, data, arch, scale) -> np.ndarray:
    if scale in ("Cons", "Just", "PV", "15"):
        return wide[arch][scale].values
    cols = ["Cons", "Just", "PV", "NAI"] if scale == "exec4" else ["Cons", "Just", "PV"]
    return data[arch][cols].values

def alpha_table(wide, data) -> pd.DataFrame:
    rows = []
    for scale in SCALES:
        for arch in ARCHS:
            a = cronbach_alpha(scale_matrix(wide, data, arch, scale))
            rows.append({"Scale": SCALE_NAMES[scale], "Architecture": arch,
                         "Cronbach_alpha": a})
    return pd.DataFrame(rows)

#
==================================================================
=============
# 7. ALPHA-DIFFERENCE TESTS (paired bootstrap CI + paired permutation p)
#
==================================================================
=============

def alpha_difference(m1: np.ndarray, m2: np.ndarray,
                     n_resamples: int = N_RESAMPLES) -> dict:
    """Both matrices are aligned on the same 500 matched execution units.
```

```
    Bootstrap: resample execution units with replacement; recompute both
    alphas on the same resample -> percentile 95% CI of (alpha2 - alpha1).
    Permutation: swap the two architectures' item vectors within randomly
    chosen execution units -> two-sided p for H0: delta alpha = 0.
    """
    n = m1.shape[0]
    d_obs = cronbach_alpha(m2) - cronbach_alpha(m1)
    boot = np.empty(n_resamples)
    for i in range(n_resamples):
        idx = rng.integers(0, n, n)
        boot[i] = cronbach_alpha(m2[idx]) - cronbach_alpha(m1[idx])
    lo, hi = np.percentile(boot, [2.5, 97.5])
    extreme = 0
    for _ in range(n_resamples):
        swap = rng.random(n) < 0.5
        a = np.where(swap[:, None], m2, m1)
        b = np.where(swap[:, None], m1, m2)
        if abs(cronbach_alpha(b) - cronbach_alpha(a)) >= abs(d_obs):
            extreme += 1
    return dict(d_alpha=d_obs, ci_low=lo, ci_high=hi,
                perm_p=extreme / n_resamples)

def alpha_diff_table(wide, data, pairs) -> pd.DataFrame:
    rows = []
    for a, b in pairs:
        for scale in SCALES:
            m1 = scale_matrix(wide, data, a, scale)
            m2 = scale_matrix(wide, data, b, scale)
            r = alpha_difference(m1, m2)
            rows.append({"Scale": SCALE_NAMES[scale], "A": a, "B": b,
                         "alpha_A": cronbach_alpha(m1),
                         "alpha_B": cronbach_alpha(m2), **r,
                         "Result": "Significant" if r["perm_p"] < .05
                                   else "Not significant"})
    return pd.DataFrame(rows)

#
============================================================
============
```

```
# 8. EXECUTION LATENCY (computational-expenditure reporting)
#
==========================================================================
=============

def latency_table(to) -> pd.DataFrame:
    rows = []
    for arch, (src, label) in ARCH_SOURCE.items():
        e = to[src].query("Arch==@label")["Elapsed"]
        rows.append({"Architecture": arch, "N": len(e), "Mean_s": e.mean(),
                     "SD_s": e.std(ddof=1), "Median_s": e.median(),
                     "Min_s": e.min(), "Max_s": e.max(),
                     "Total_hours": e.sum() / 3600})
    return pd.DataFrame(rows)

#
==========================================================================
=============
# 9. FIGURES
#
==========================================================================
=============

def make_figures(data, alphas: pd.DataFrame):
    import matplotlib
    matplotlib.use("Agg")
    import matplotlib.pyplot as plt
    plt.rcParams.update({"font.family": "DejaVu Sans", "font.size": 10,
                         "axes.spines.top": False, "axes.spines.right": False})
    core = ["Cons", "Just", "PV", "NAI", "Overall"]
    labels = ["Consistency", "Justification", "Procedural₩nValidity",
              "NAI", "Overall₩nMean"]
    colors = {"Single": "#6E7B8B", "Multi": "#C97B4A",
              "Multi-LLM": "#4A8BC9", "Multi-LLM-KRB": "#2166AC"}

    # Figure 1 — mean score profiles
    fig, ax = plt.subplots(figsize=(7.2, 4.2))
    x = np.arange(len(core))
    for arch in ARCHS:
```

```
    means = [data[arch][m].mean() for m in core]
    ax.plot(x, means, marker="o", label=arch, color=colors[arch], lw=1.8)
ax.set_xticks(x); ax.set_xticklabels(labels)
ax.set_ylabel("Mean score (1–5)"); ax.set_ylim(3.8, 5.0)
ax.legend(frameon=False, fontsize=9)
ax.set_title("Mean score profiles across the four CREA architectures")
ax.grid(axis="y", color="#E4E7EB", lw=0.6); ax.set_axisbelow(True)
fig.tight_layout(); fig.savefig("fig_mean_profiles.png", dpi=200)
plt.close(fig)

# Figure 2 — overall means with 95% CIs
fig, ax = plt.subplots(figsize=(6.4, 4.0))
for i, arch in enumerate(ARCHS):
    x_ = data[arch]["Overall"]
    m = x_.mean()
    half = stats.t.ppf(0.975, len(x_) - 1) * x_.std(ddof=1) / np.sqrt(len(x_))
    ax.bar(i, m, 0.6, color=colors[arch])
    ax.errorbar(i, m, yerr=half, color="#222222", capsize=4, lw=1.2)
    ax.text(i, m + 0.05, f"{m:.3f}", ha="center", fontsize=9)
ax.set_xticks(range(4)); ax.set_xticklabels(ARCHS, fontsize=9)
ax.set_ylabel("Overall mean (1–5)"); ax.set_ylim(4.0, 4.75)
ax.set_title("Overall mean scores by architecture with 95% CIs")
ax.grid(axis="y", color="#E4E7EB", lw=0.6); ax.set_axisbelow(True)
fig.tight_layout(); fig.savefig("fig_overall_ci.png", dpi=200)
plt.close(fig)

# Figure 3 — metric-specific alpha by architecture
fig, ax = plt.subplots(figsize=(7.2, 4.0))
scales3 = ["Consistency (5 items)", "Justification (5 items)",
           "Procedural validity (5 items)", "15-item measurement scale"]
w = 0.19
for j, arch in enumerate(ARCHS):
    vals = [alphas[(alphas["Scale"] == s)
                   & (alphas["Architecture"] == arch)]
            ["Cronbach_alpha"].iloc[0] for s in scales3]
    ax.bar(np.arange(len(scales3)) + (j - 1.5) * w, vals, w,
           label=arch, color=colors[arch])
ax.set_xticks(range(len(scales3)))
ax.set_xticklabels(["Consistency", "Justification",
```

```
                    "Procedural validity", "15-item scale"], fontsize=9)
ax.set_ylabel("Cronbach's α"); ax.set_ylim(0.6, 1.0)
ax.axhline(0.7, color="#999999", lw=0.8, ls=":")
ax.legend(frameon=False, fontsize=8, ncols=2)
ax.set_title("Internal consistency (Cronbach's α) by architecture")
ax.grid(axis="y", color="#E4E7EB", lw=0.6); ax.set_axisbelow(True)
fig.tight_layout(); fig.savefig("fig_alpha.png", dpi=200)
plt.close(fig)

# Figure 4 — box plots (overall mean; metric-wise)
fig, ax = plt.subplots(figsize=(6.4, 4.0))
bp = ax.boxplot([data[a]["Overall"] for a in ARCHS], labels=ARCHS,
                patch_artist=True, widths=0.55,
                flierprops=dict(marker="o", markersize=3, alpha=0.5))
for patch, arch in zip(bp["boxes"], ARCHS):
    patch.set_facecolor(colors[arch]); patch.set_alpha(0.55)
for med in bp["medians"]:
    med.set_color("#222222")
ax.set_ylabel("Overall mean (1–5)"); ax.tick_params(axis="x", labelsize=9)
ax.set_title("Overall mean score distributions by architecture")
ax.grid(axis="y", color="#E4E7EB", lw=0.6); ax.set_axisbelow(True)
fig.tight_layout(); fig.savefig("fig_box_overall.png", dpi=200)
plt.close(fig)

fig, axes = plt.subplots(1, 5, figsize=(13.0, 3.6), sharey=False)
for ax, m, lab in zip(axes, core, ["Consistency", "Justification",
                                   "Procedural Validity", "NAI",
                                   "Overall Mean"]):
    bp = ax.boxplot([data[a][m] for a in ARCHS], patch_artist=True,
                    widths=0.6, flierprops=dict(marker="o", markersize=2,
                                                alpha=0.4))
    for patch, arch in zip(bp["boxes"], ARCHS):
        patch.set_facecolor(colors[arch]); patch.set_alpha(0.55)
    for med in bp["medians"]:
        med.set_color("#222222")
    ax.set_title(lab, fontsize=10)
    ax.set_xticklabels(["S", "M", "ML", "ML+₩nKRB"], fontsize=8)
    ax.grid(axis="y", color="#E4E7EB", lw=0.6); ax.set_axisbelow(True)
fig.suptitle("Metric-wise score distributions across the four architectures",
```

```
                    y=1.02, fontsize=12)
    fig.tight_layout()
    fig.savefig("fig_box_metrics.png", dpi=200, bbox_inches="tight")
    plt.close(fig)
    print("   figures written: fig_mean_profiles / fig_overall_ci / "
          "fig_alpha / fig_box_overall / fig_box_metrics")

#
=================================================================
=============
# MAIN
#
=================================================================
=============

def main():
    print("1) Loading raw files ...")
    ev, na, to = load_all()

    print("2) Validating duplicate architecture sources ...")
    for name, maxdiff in validate_duplicate_sources(ev, na):
        status = "OK" if maxdiff == 0 else f"MISMATCH (max |diff| = {maxdiff})"
        print(f"     {name}: {status}")

    print("3) Reconstructing execution-level dataset from evaluator raw data ...")
    data = build_execution_level(ev, na)

    print("4) Descriptive statistics ...")
    desc = descriptives(data)

    print("5) Omnibus Friedman tests ...")
    fried = friedman_tests(data)
    print(fried.round(3).to_string(index=False))

    print("6) Pairwise comparisons (6 per metric, Holm-adjusted) ...")
    pw = pairwise_table(data)

    print("7) Linear mixed-effects robustness checks ...")
    lmm = lmm_table(data)
```

```python
    print("8) Cronbach's alpha ...")
    wide = {a: evaluator_wide(ev, a) for a in ARCHS}
    alph = alpha_table(wide, data)

    print(f"9) Alpha-difference tests ({N_RESAMPLES} paired bootstrap / "
          f"permutation resamples) ...")
    adiff_adj = alpha_diff_table(wide, data, ADJACENT_PAIRS)
    adiff_all = alpha_diff_table(wide, data, PAIRS)

    print("10) Execution latency ...")
    lat = latency_table(to)

    print(f"11) Exporting tables to {OUTPUT_XLSX} ...")
    with pd.ExcelWriter(OUTPUT_XLSX) as w:
        desc.round(4).to_excel(w, sheet_name="Descriptive", index=False)
        fried.round(6).to_excel(w, sheet_name="Friedman_Omnibus", index=False)
        pw.round(6).to_excel(w, sheet_name="Pairwise_Comparisons", index=False)
        lmm.round(6).to_excel(w, sheet_name="LMM_Robustness", index=False)
        alph.round(4).to_excel(w, sheet_name="Cronbach_Alpha", index=False)
        adiff_adj.round(4).to_excel(w, sheet_name="Alpha_Diff_Adjacent", index=False)
        adiff_all.round(4).to_excel(w, sheet_name="Alpha_Diff_AllPairs", index=False)
        lat.round(3).to_excel(w, sheet_name="Latency", index=False)

    if MAKE_FIGURES:
        print("12) Figures ...")
        make_figures(data, alph)

    print("Done.")

if __name__ == "__main__":
    main()
```
