# Supplementary material for: Implementing a Cognitively Grounded Artificial Moral Advisor: A Multi-LLM Multi-Agent Approach Based on the Cognitive–Reflective Equilibration Model
Source: J Intell. 2026 Sep 2;14(9):212. doi: 10.3390/jintelligence14090212 (PMC13608209; doi:10.3390/jintelligence14090212)
Supplement: Supplementary file 1 [file jintelligence-14-00212-s001.zip › Document_S3.Full_pairwise_analysis_Python_Sourcecode.pdf]

Document_S2.Full_pairwise_analysis_Python_Sourcecode

```
import pandas as pd, numpy as np, json, warnings
from scipy import stats
warnings.filterwarnings("ignore")
rng = np.random.default_rng(20260805)

# ---------- load ----------
def load_ev(f):
    df = pd.ExcelFile(f).parse(0)
    df.columns = ["Dilemma","Arch","ExLLM","Round","Evaluator","Cons","Just","PV"]
    return df
def load_nai(f):
    df = pd.ExcelFile(f).parse(0)
    df.columns = ["Dilemma","Arch","ExLLM","Round","NAI"]
    return df
def load_tot(f):
    df = pd.ExcelFile(f).parse(0)
    df.columns =
["No","DateTime","Arch","Dilemma","ExLLM","Round","Cons","Just","PV","NAI","Elapsed"]
    return df

ev = {f: load_ev(f+"_Cons_Just_PV.xlsx") for f in
["01_S_MAgent","01_S_MLLM","01_S_MLLMKRB","02_MMLLM","03_MLLMMLLMKRB"]}
na = {f: load_nai(f+"_NAI.xlsx") for f in ev}
to = {f: load_tot(f+"_Tot.xlsx") for f in ev}

KEY = ["Dilemma","ExLLM","Round"]

# ---------- duplicate-source verification ----------
def exec_from_ev(df, arch):
    d = df[df.Arch==arch].groupby(KEY)[["Cons","Just","PV"]].mean().sort_index()
    return d
checks = []
# 01 triples identical?
for f in ["01_S_MLLM","01_S_MLLMKRB"]:
    same =
ev[f].sort_values(KEY+["Arch","Evaluator"]).reset_index(drop=True)[["Cons","Just","PV"]].equals(

ev["01_S_MAgent"].sort_values(KEY+["Arch","Evaluator"]).reset_index(drop=True)[["Cons","Just","P
V"]])
    checks.append((f"{f} == 01_S_MAgent (evaluator)", bool(same)))
# Multi: 01 vs 02
a = exec_from_ev(ev["01_S_MAgent"],"Multi"); b = exec_from_ev(ev["02_MMLLM"],"Multi")
checks.append(("Multi: 01 vs 02 max|diff|", float((a-b).abs().max().max())))
# MLLM: 02 vs 03
a = exec_from_ev(ev["02_MMLLM"],"Multi-LLM"); b = exec_from_ev(ev["03_MLLMMLLMKRB"],"Multi-LLM")
checks.append(("Multi-LLM: 02 vs 03 max|diff|", float((a-b).abs().max().max())))
# NAI: Multi 01 vs 02, MLLM 02 vs 03
a = na["01_S_MAgent"].query("Arch=='Multi'").set_index(KEY)["NAI"].sort_index()
b = na["02_MMLLM"].query("Arch=='Multi'").set_index(KEY)["NAI"].sort_index()
```

```
checks.append(("Multi NAI: 01 vs 02 max|diff|", float((a-b).abs().max())))
a = na["02_MMLLM"].query("Arch=='Multi-LLM'").set_index(KEY)["NAI"].sort_index()
b = na["03_MLLMMLLMKRB"].query("Arch=='Multi-LLM'").set_index(KEY)["NAI"].sort_index()
checks.append(("MLLM NAI: 02 vs 03 max|diff|", float((a-b).abs().max())))
for c in checks: print("CHECK", c)

# ---------- canonical evaluator-level per architecture ----------
EV = {
 "Single":    ev["01_S_MAgent"][ev["01_S_MAgent"].Arch=="Single"],
 "Multi":     ev["01_S_MAgent"][ev["01_S_MAgent"].Arch=="Multi"],
 "Multi-LLM": ev["02_MMLLM"][ev["02_MMLLM"].Arch=="Multi-LLM"],
 "Multi-LLM-KRB": ev["03_MLLMMLLMKRB"][ev["03_MLLMMLLMKRB"].Arch=="Multi-LLM-KRB"],
}
NAI_RAW = {
 "Single": na["01_S_MAgent"].query("Arch=='Single'"),
 "Multi": na["01_S_MAgent"].query("Arch=='Multi'"),
 "Multi-LLM": na["02_MMLLM"].query("Arch=='Multi-LLM'"),
 "Multi-LLM-KRB": na["03_MLLMMLLMKRB"].query("Arch=='Multi-LLM-KRB'"),
}

# v3-documented corrections for Multi-LLM / GPT / R2 (Table C)
corr = {
 "1. Human_Medical":4.22,"2. Human_Environment":3.84,"3. Human_Counseling":4.42,
 "4. Human_Social Welfare":3.88,"5. Human_School":4.18,"6. Human_War":3.44,
 "7. Human_Rescue":4.18,"8. Human_Finance":4.67,"9. Human_Construction":4.67,
 "10. Human_Job":4.44,"11. AI_AI Recruitment":4.38,"12. AI_AI Speaker":4.02,
 "13. AI_Face Recognition CCTV":4.16,"14. AI_Police Robot":4.09,"15. AI_Deepfake":4.04,
 "16. AI_Cleaning Robot":3.94,"17. AI_Drone Delivery":4.2,"18. AI_AI Weapon":4.11,
 "19. AI_AI Generated Image":3.82,"20. AI_Care Robot":3.8,
}
def apply_corr(df):
    df = df.copy()
    m = (df.ExLLM=="GPT") & (df.Round=="R2")
    matched = 0
    for i in df[m].index:
        dil = df.at[i,"Dilemma"]
        if dil in corr:
            df.at[i,"NAI"] = corr[dil]; matched += 1
    return df, matched
NAI_COR = dict(NAI_RAW)
NAI_COR["Multi-LLM"], nmatch = apply_corr(NAI_RAW["Multi-LLM"])
print("CHECK corrected NAI rows applied:", nmatch,
      "| MLLM NAI raw mean:", round(NAI_RAW["Multi-LLM"].NAI.mean(),4),
      "corrected mean:", round(NAI_COR["Multi-LLM"].NAI.mean(),4))
# how many of the raw values equal the 'old' values in Table C? (sanity)
old_tab = {"1. Human_Medical":4.8,"20. AI_Care Robot":4.16,"19. AI_AI Generated Image":4.87}
sub = NAI_RAW["Multi-LLM"].query("ExLLM=='GPT' and Round=='R2'").set_index("Dilemma")["NAI"]
print("CHECK sample raw NAI values (expect pre-correction):", {k: float(sub.get(k, np.nan)) for k
in old_tab})

# ---------- execution-level dataset builder ----------
def build(arch, nai_src):
```

```
    e = EV[arch].groupby(KEY)[["Cons","Just","PV"]].mean()
    n = nai_src[arch].set_index(KEY)["NAI"]
    d = e.join(n).reset_index()
    d["Overall"] = d[["Cons","Just","PV","NAI"]].mean(axis=1)
    d["Arch"] = arch
    return d.set_index(KEY).sort_index()

DATA_C = {a: build(a, NAI_COR) for a in EV}   # corrected NAI (primary)
DATA_R = {a: build(a, NAI_RAW) for a in EV}   # raw NAI (sensitivity)

METRICS = ["Cons","Just","PV","NAI","Overall"]
MNAMES = {"Cons":"Consistency","Just":"Justification","PV":"Procedural
validity","NAI":"NAI","Overall":"Overall mean"}
PAIRS = [("Single","Multi"),("Multi","Multi-LLM"),("Multi-LLM","Multi-LLM-KRB"),
         ("Single","Multi-LLM"),("Single","Multi-LLM-KRB"),("Multi","Multi-LLM-KRB")]

def paired_stats(A, B, m):
    x = A[m]; y = B[m]
    assert x.index.equals(y.index) and len(x)==500
    d = (y - x).values
    n = len(d); md = d.mean(); sd = d.std(ddof=1)
    t = md / (sd/np.sqrt(n)); p = 2*stats.t.sf(abs(t), n-1)
    ci = stats.t.ppf(0.975, n-1) * sd/np.sqrt(n)
    dz = md/sd
    try:
        wp = stats.wilcoxon(y.values, x.values).pvalue
    except ValueError:
        wp = np.nan
    return dict(A_mean=x.mean(), B_mean=y.mean(), diff=md, lo=md-ci, hi=md+ci, t=t, df=n-1, p=p,
dz=dz, wilcoxon_p=wp)

def holm(pvals):
    idx = np.argsort(pvals); m = len(pvals); adj = np.empty(m); mx = 0
    for r, i in enumerate(idx):
        v = min(1.0, (m-r)*pvals[i]); mx = max(mx, v); adj[i] = mx
    return adj

def run_pairwise(DATA):
    rows = []
    for m in METRICS:
        res = [paired_stats(DATA[a], DATA[b], m) for a,b in PAIRS]
        adj = holm([r["p"] for r in res])
        for (a,b), r, hp in zip(PAIRS, res, adj):
            rows.append(dict(metric=MNAMES[m], A=a, B=b, **r, holm_p=hp))
    return pd.DataFrame(rows)

PW_C = run_pairwise(DATA_C)
PW_R = run_pairwise(DATA_R)
print("₩n===== PAIRWISE (corrected NAI) ====="); print(PW_C.round(4).to_string())
print("₩n===== NAI/Overall rows (raw NAI sensitivity) =====")
print(PW_R[PW_R.metric.isin(["NAI","Overall mean"])].round(4).to_string())
```

```
# ---------- LMM ----------
import statsmodels.formula.api as smf
lmm_rows = []
for a,b in PAIRS:
    A = DATA_C[a].reset_index(); B = DATA_C[b].reset_index()
    dd = pd.concat([A,B])
    dd["arch"] = pd.Categorical(dd["Arch"], categories=[a,b])
    for m in METRICS:
        md    =    smf.mixedlm(f"{m}    ~    C(arch)    +    C(ExLLM)    +    C(Round)",    dd,
groups=dd["Dilemma"]).fit(reml=True)
        cn = [c for c in md.params.index if c.startswith("C(arch)")][0]
        lmm_rows.append(dict(A=a,B=b,metric=MNAMES[m],beta=md.params[cn],se=md.bse[cn],
                             z=md.params[cn]/md.bse[cn],p=md.pvalues[cn],
                             var_dilemma=float(md.cov_re.iloc[0,0]),sigma2=float(md.scale)))
LMM = pd.DataFrame(lmm_rows)
print("₩n===== LMM ====="); print(LMM.round(4).to_string())

# ---------- Cronbach alpha ----------
def alpha(mat):  # rows obs, cols items
    k = mat.shape[1]
    vi = mat.var(axis=0, ddof=1).sum(); vt = mat.sum(axis=1).var(ddof=1)
    return k/(k-1)*(1 - vi/vt)

def wide_ev(arch):  # dict metric -> 500x5 matrix aligned on KEY
    d = EV[arch].copy()
    out = {}
    for m in ["Cons","Just","PV"]:
        w = d.pivot_table(index=KEY, columns="Evaluator", values=m).sort_index()
        out[m] = w
    out["15"] = pd.concat([out["Cons"],out["Just"],out["PV"]], axis=1)
    return out
WIDE = {a: wide_ev(a) for a in EV}
def exec_mat(arch, k):
    d = DATA_C[arch]
    cols = ["Cons","Just","PV","NAI"] if k==4 else ["Cons","Just","PV"]
    return d[cols]

SCALES = ["Cons","Just","PV","15","exec4","exec3"]
SNAMES = {"Cons":"Consistency (5)","Just":"Justification (5)","PV":"Procedural validity (5)",
          "15":"15-item scale","exec4":"Exec 4-indicator","exec3":"Exec 3-indicator"}
def get_mat(arch, s):
    if s in ("Cons","Just","PV","15"): return WIDE[arch][s].values
    return exec_mat(arch, 4 if s=="exec4" else 3).values

ALPHAS = {(a,s): alpha(get_mat(a,s)) for a in EV for s in SCALES}
print("₩n===== ALPHA by architecture =====")
for s in SCALES:
    print(SNAMES[s], {a: round(ALPHAS[(a,s)],3) for a in EV})

NBOOT = 3000
def alpha_diff_test(a1, a2, s):
    M1 = get_mat(a1,s); M2 = get_mat(a2,s)
```

```
    n = M1.shape[0]
    d_obs = alpha(M2) - alpha(M1)
    boots = np.empty(NBOOT)
    for i in range(NBOOT):
        idx = rng.integers(0, n, n)
        boots[i] = alpha(M2[idx]) - alpha(M1[idx])
    lo, hi = np.percentile(boots, [2.5, 97.5])
    cnt = 0
    for i in range(NBOOT):
        sw = rng.random(n) < 0.5
        A = np.where(sw[:,None], M2, M1); B = np.where(sw[:,None], M1, M2)
        if abs(alpha(B) - alpha(A)) >= abs(d_obs): cnt += 1
    return d_obs, lo, hi, cnt/NBOOT

ad_rows = []
for a,b in PAIRS:
    for s in SCALES:
        d0, lo, hi, pp = alpha_diff_test(a,b,s)
        ad_rows.append(dict(A=a,B=b,scale=SNAMES[s],alpha_A=ALPHAS[(a,s)],alpha_B=ALPHAS[(b,s)],
                            d_alpha=d0,lo=lo,hi=hi,perm_p=pp))
        print("ADIFF", a, b, SNAMES[s], round(d0,3), f"[{lo:.3f},{hi:.3f}]", "p=", round(pp,3))
ADIFF = pd.DataFrame(ad_rows)

# ---------- elapsed time (R1-13) ----------
TOT = {
 "Single": to["01_S_MAgent"].query("Arch=='Single'"),
 "Multi": to["01_S_MAgent"].query("Arch=='Multi'"),
 "Multi-LLM": to["02_MMLLM"].query("Arch=='Multi-LLM'"),
 "Multi-LLM-KRB": to["03_MLLMMLLMKRB"].query("Arch=='Multi-LLM-KRB'"),
}
el_rows = []
for a, d in TOT.items():
    e = d["Elapsed"]
    el_rows.append(dict(Arch=a, N=len(e), mean=e.mean(), sd=e.std(ddof=1), median=e.median(),
                    min=e.min(), max=e.max(), total_hours=e.sum()/3600))
ELAPSED = pd.DataFrame(el_rows)
print("₩n===== ELAPSED ====="); print(ELAPSED.round(2).to_string())

# ---------- descriptives ----------
desc_rows = []
for a in EV:
    d = DATA_C[a]
    for m in METRICS:
        x = d[m]
        desc_rows.append(dict(Arch=a, metric=MNAMES[m], N=len(x), mean=x.mean(), sd=x.std(ddof=1),
                              median=x.median(), min=x.min(), max=x.max()))
DESC = pd.DataFrame(desc_rows)

# ---------- export ----------
with pd.ExcelWriter("Full_pairwise_reanalysis_from_raw.xlsx") as w:
    DESC.round(4).to_excel(w, sheet_name="Descriptive", index=False)
    PW_C.round(6).to_excel(w, sheet_name="Pairwise_correctedNAI", index=False)
```

```
    PW_R.round(6).to_excel(w, sheet_name="Pairwise_rawNAI", index=False)
    LMM.round(6).to_excel(w, sheet_name="LMM", index=False)
    pd.DataFrame([{ "arch":a, "scale":SNAMES[s], "alpha":round(ALPHAS[(a,s)],4)} for a in EV for
s in SCALES]).to_excel(w, sheet_name="Alpha", index=False)
    ADIFF.round(4).to_excel(w, sheet_name="Alpha_diff", index=False)
    ELAPSED.round(3).to_excel(w, sheet_name="Elapsed", index=False)
json.dump({
  "pairwise_corrected": PW_C.to_dict("records"),
  "pairwise_raw": PW_R.to_dict("records"),
  "lmm": LMM.to_dict("records"),
  "alpha": {f"{a}|{s}": ALPHAS[(a,s)] for a in EV for s in SCALES},
  "alpha_diff": ADIFF.to_dict("records"),
  "elapsed": ELAPSED.to_dict("records"),
}, open("results.json","w"), default=float)
print("₩nEXPORTED")
```
