# Supplementary material for: Implementing a Cognitively Grounded Artificial Moral Advisor: A Multi-LLM Multi-Agent Approach Based on the Cognitive–Reflective Equilibration Model
Source: J Intell. 2026 Sep 2;14(9):212. doi: 10.3390/jintelligence14090212 (PMC13608209; doi:10.3390/jintelligence14090212)
Supplement: Supplementary file 1 [file jintelligence-14-00212-s001.zip › Document_S4.MultiLLM_latency_cost_analysis_Python_Sourcecode.pdf]

Document_S3. MultiLLM_latency_cost_analysis_Python_Sourcecode

```
#!/usr/bin/env python3
# -*- coding: utf-8 -*-
"""
build_table_s3.py
==============================================================================
Regenerates Table S3 — "MultiLLM calls, tokens, latency, cost: total data" —
from the stored CREA execution records (reasonbank JSON files).

Output workbook, eleven sheets:

    Unit_Prices              editable unit prices; every cost figure recalculates
                              from these five rows
    T1_Condition_Summary      calls, tokens, latency and cost by condition
    Single_by_LLM             executor-side detail, single-agent condition
    Multi_by_LLM              executor-side detail, multi-agent condition
    MLLM_by_LLM               executor-side detail, multi-LLM condition
    KRB_by_LLM                executor-side detail, multi-LLM+KRB condition
    Measurement_Cost          evaluator-side call breakdown and cost
    Provenance                data sources, measurement rules, caveats
    Truncation                step-output length vs the 400-character bundle limit
    Measurement_Forensics     signatures of the two procedural-validity regimes
    Per_Run_All               one row per execution unit (2,000 rows)

------------------------------------------------------------------------------
INPUT
------------------------------------------------------------------------------
One JSON file per execution unit, written by the system at the end of each run:

    agent_mode        'single' | 'multi'
    llm_type          executor model key
    sub_llms          auxiliary models used at fan-out steps ([] outside the
                      two multi-LLM conditions)
    kb_selection      knowledge-base selection (None when EPKB is unused)
    use_rb            True only in the Multi-LLM+KRB condition
    elapsed           wall-clock seconds
    timestamp         ISO 8601 start time
```

crem_result {'ethical_dilemma', 'R1','A1', ...}; only executed steps
are present, so the A-keys give the executed-step count
evaluation_scores consistency / justifiability / procedural_validity / nai
evaluation_output the four aggregate means

The architectural condition is read from the record itself rather than from folder names:

agent_mode == 'single' -> Single-Agent
use_rb is True -> Multi-LLM+KRB
sub_llms non-empty -> Multi-LLM
otherwise -> Multi-Agent

COUNTING RULES

Executor calls
steps one synthesis call per recorded R/A pair
aux len(sub_llms) x number of executed fan-out steps
(fan-out steps are 3, 11, 12, 13, 18)
exec_calls steps + aux

Executor tokens
up upper bound: the full accumulated transcript is resent at each step
lo lower bound: dilemma + own request + preceding answer only
out the step responses
The cost columns use the upper bound.

Measurement calls (five evaluators throughout)
consistency 5
justifiability 5
procedural validity 5 single-agent: all twenty step slots
go into ONE prompt per evaluator
5 x executed steps multi-agent family: one prompt per
executed step per evaluator
NAI relevance 5 x total_principles
NAI alignment 5 x filtered_count

Measurement tokens

mout the stored evaluator reason text, summed over every response.
Procedural-validity slots recorded as "not executed" are excluded.
Verified against the reference workbook on the 500 single-agent runs:
mean absolute error 0.02%, condition total within 30 tokens.

min_ reconstructed, because the records preserve every evaluator RESPONSE
but not the PROMPT that produced it:

```
min_ = 5 x [ dilemma
            + 2 x (steps 18-20)            consistency + justifiability
            + bundled 20-step prompt         single-agent only,
                                             each step truncated to 400 chars
            + BUNDLE_INSTRUCTION_TOKENS ]
      + 5 x (principles + filtered) x NAI_CONTEXT_TOKENS
```

In the multi-agent family the bundled term is replaced by the sum of
the individual step texts, one prompt per step.
The two constants below were calibrated against the reference
workbook on the 500 single-agent runs: mean absolute error 0.22%,
maximum 0.76%, condition total within one token.

Cost

cost = input / 1e6 * price_in + output / 1e6 * price_out, at the rates in effect on the execution dates. Executor cost is charged to the executing model. Measurement cost is charged at the mean of the five evaluator prices, since every evaluator receives the same prompt.

Deduplication

Records are keyed on (condition, executor, round, dilemma) and the latest timestamp is kept: 2,140 stored files -> 2,000 analysed execution units.
The Truncation and Measurement_Forensics sheets deliberately use ALL stored records, including superseded retries, because they describe the evaluator output rather than the experiment.

USAGE

```
python build_table_s3.py --root <reasonbank archives> ₩
                         --out Table_S3_calls_tokens_latency_cost.xlsx
```

```
    python build_table_s3.py --root <...> --validate <reference workbook>

Requires: python >= 3.10, pandas, numpy, openpyxl
--------------------------------------------------------------------------------
"""

from __future__ import annotations

import argparse
import json
import re
import sys
from collections import defaultdict
from pathlib import Path

import numpy as np
import pandas as pd
import openpyxl
from openpyxl.styles import Alignment, Border, Font, PatternFill, Side
from openpyxl.utils import get_column_letter

#
==========================================================================
=============
# CONFIGURATION
#
==========================================================================
=============

FANOUT_STEPS = (3, 11, 12, 13, 18)          # steps that fan out to auxiliary models
N_EVALUATORS = 5                              # evaluator ensemble size
BUNDLE_TRUNCATION = 400                       # characters kept per step in the bundle

#: Calibrated prompt constants (see the docstring).   Both are properties of the
#: measurement prompt templates, not of the data.
BUNDLE_INSTRUCTION_TOKENS = 278               # fixed instructions in a bundled prompt
NAI_CONTEXT_TOKENS = 270                      # context carried by one NAI call

#: Unit prices, USD per 1M tokens, in effect on the execution dates.
```

```
PRICES = {
    "GPT":      dict(model="GPT-4.1",          inp=2.00, out=8.00,
                     src="Standard uncached API rate; cached input $0.50/M; Batch API discount
excluded."),
    "Claude":   dict(model="Claude Haiku 4.5", inp=1.00, out=5.00,
                     src="Claude API standard global rate; prompt caching and batch discounts
excluded."),
    "Gemini":   dict(model="Gemini 2.5 Flash", inp=0.30, out=2.50,
                     src="Stable Gemini 2.5 Flash rate; output price includes thinking tokens."),
    "Grok":     dict(model="Grok-3",           inp=3.00, out=15.00,
                     src="Original Grok-3 rate; cached input $0.75/M. From 2026-05-15 the grok-
3 slug was "
                         "billed as Grok-4.3 at $1.25/M input and $2.50/M output; only 9 of 400
Grok "
                         "executions post-date that change."),
    "DeepSeek": dict(model="DeepSeek V3",      inp=0.27, out=1.10,
                     src="Official DeepSeek-V3 list rate; input uses cache-miss price (cache hit
$0.07/M)."),
}
LLM_ORDER = ["GPT", "Claude", "Gemini", "Grok", "DeepSeek"]
EXECUTOR_ALIASES = {"gpt": "GPT", "claude": "Claude", "gemini": "Gemini",
                    "grok": "Grok", "deepseek": "DeepSeek"}

#: (internal tag, sheet name, display label)
ARCHITECTURES = [
    ("Single", "Single_by_LLM", "Single-Agent"),
    ("Multi",  "Multi_by_LLM",  "Multi-Agent"),
    ("MLLM",   "MLLM_by_LLM",   "Multi-LLM"),
    ("KRB",    "KRB_by_LLM",    "Multi-LLM+KRB"),
]
TAG_LABEL = {t: l for t, _, l in ARCHITECTURES}


#
==============================================================
=============
# TOKEN ESTIMATION
#
==============================================================
```

```
=============

def est_tokens(text) -> float:
    """max(words * 1.3, characters / 4).

    Used because provider tokenizers were unavailable in the offline analysis
    environment; agrees with tiktoken to roughly +/-15% on English prose.
    """
    if not text:
        return 0.0
    s = str(text)
    return max(len(s.split()) * 1.3, len(s) / 4.0)


#
=================================================================
=============
# LOADING
#
=================================================================
=============

def condition_tag(record: dict) -> str:
    if str(record.get("agent_mode", "")).lower() == "single":
        return "Single"
    if record.get("use_rb"):
        return "KRB"
    return "MLLM" if (record.get("sub_llms") or []) else "Multi"


def parse_round(path: Path) -> str:
    for p in path.parents:
        m = re.fullmatch(r"R([1-5])", p.name.strip())
        if m:
            return f"R{m.group(1)}"
    return "R?"


def dilemma_label(record: dict) -> str:
```

```
    text = str(record.get("dilemma", "")).strip()
    first = text.split("₩n", 1)[0].strip()
    return first if first else (record.get("dilemma_title") or "unknown")


def load_records(root: Path) -> list[dict]:
    out = []
    for fp in sorted(root.rglob("*.json")):
        if not fp.name.lower().startswith("reasonbank"):
            continue
        try:
            rec = json.loads(fp.read_text(encoding="utf-8"))
        except (json.JSONDecodeError, UnicodeDecodeError) as exc:
            print(f"   ! unreadable, skipped: {fp} ({exc})", file=sys.stderr)
            continue
        rec["_path"] = str(fp)
        rec["_arch"] = condition_tag(rec)
        rec["_round"] = parse_round(fp)
        rec["_llm"] = EXECUTOR_ALIASES.get(str(rec.get("llm_type", "")).lower(), "?")
        rec["_dilemma"] = dilemma_label(rec)
        out.append(rec)
    return out


def deduplicate(records: list[dict]) -> list[dict]:
    keep: dict[tuple, dict] = {}
    for rec in records:
        key = (rec["_arch"], rec["_llm"], rec["_round"], rec["_dilemma"])
        prev = keep.get(key)
        if prev is None or str(rec.get("timestamp", "")) > str(prev.get("timestamp", "")):
            keep[key] = rec
    print(f"   deduplication: {len(records)} files -> {len(keep)} execution units")
    return list(keep.values())


#
===============================================================================
=============
# PER-RECORD MEASUREMENT
```

```
#
==========================================================================
=============

def executed_steps(record: dict) -> list[int]:
    cr = record.get("crem_result") or {}
    return sorted(int(m.group(1)) for k in cr if (m := re.fullmatch(r"A(₩d{1,2})", k)))


def evaluator_entries(node) -> list[dict]:
    """Per-evaluator dicts inside a score node, skipping aggregates and flags."""
    if not isinstance(node, dict):
        return []
    return [v for k, v in node.items()
            if k not in ("stats", "executed", "Ri", "Ai", "per_llm",
                         "alignment_stats", "relevance_llms")
            and isinstance(v, dict) and "score" in v]


def reference_counts(record: dict, steps: list[int]) -> tuple[int, int]:
    """Steps whose output cites a knowledge-base or reasoning-bank reference."""
    cr = record.get("crem_result") or {}
    kb = rb = 0
    kb_pat = re.compile(r"knowledge base|EPKB|₩[KB", re.I)
    rb_pat = re.compile(r"reasoning bank|prior case|ERB|₩[RB", re.I)
    for s in steps:
        text = str(cr.get(f"A{s}") or "")
        if kb_pat.search(text):
            kb += 1
        if rb_pat.search(text):
            rb += 1
    return kb, rb


def measure_record(record: dict) -> dict:
    """All Per_Run_All columns for one execution unit."""
    cr = record.get("crem_result") or {}
    es = record.get("evaluation_scores") or {}
    nai = es.get("nai") or {}
```

```
steps = executed_steps(record)
bundled = record["_arch"] == "Single"

# ---- executor side ----
ensemble = len(record.get("sub_llms") or [])
aux = ensemble * sum(1 for s in steps if s in FANOUT_STEPS)

dilemma_tok = est_tokens(cr.get("ethical_dilemma") or record.get("dilemma"))
up = lo = out = 0.0
context = dilemma_tok
previous_answer = 0.0
for s in steps:
    req = est_tokens(cr.get(f"R{s}"))
    ans = est_tokens(cr.get(f"A{s}"))
    up += context + req
    lo += dilemma_tok + req + previous_answer
    context += req + ans
    previous_answer = ans
    out += ans

# ---- measurement calls ----
n_principles = int(nai.get("total_principles") or 0)
n_filtered = int(nai.get("filtered_count") or 0)
pv_scores = N_EVALUATORS * len(steps)          # procedural-validity scores
pv_calls = N_EVALUATORS if bundled else pv_scores
rel_calls = N_EVALUATORS * n_principles
align_calls = N_EVALUATORS * n_filtered
mcalls = 2 * N_EVALUATORS + pv_calls + rel_calls + align_calls

# ---- measurement input (reconstructed) ----
closing = sum(est_tokens(cr.get(f"A{s}")) for s in (18, 19, 20) if f"A{s}" in cr)
if bundled:
    pv_material = sum(est_tokens(str(cr.get(f"A{s}", ""))[:BUNDLE_TRUNCATION]) for s in steps)
else:
    pv_material = sum(est_tokens(cr.get(f"A{s}")) for s in steps)
min_ = (N_EVALUATORS * (est_tokens(record.get("dilemma"))
                        + 2 * closing
                        + pv_material
                        + BUNDLE_INSTRUCTION_TOKENS)
```

```
            + (rel_calls + align_calls) * NAI_CONTEXT_TOKENS)

    # ---- measurement output (stored evaluator text) ----
    mout = 0.0
    for component in ("consistency", "justifiability"):
        mout += sum(est_tokens(e.get("reason")) for e in evaluator_entries(es.get(component) or
{}))
    for node in ((es.get("procedural_validity") or {}).get("per_step") or {}).values():
        mout += sum(est_tokens(e.get("reason")) for e in evaluator_entries(node)
                    if e.get("score") not in ("N/A", None))
    for node in (nai.get("all_relevance") or {}).values():
        mout += sum(est_tokens(e.get("reason")) for e in evaluator_entries(node))
    for node in (nai.get("alignment_results") or {}).values():
        mout += sum(est_tokens(v.get("reason")) for v in (node.get("per_llm") or {}).values()
                    if isinstance(v, dict))

    kb, rb = reference_counts(record, steps)

    return {
        "arch": record["_arch"], "ExLLM": record["_llm"], "round": record["_round"],
        "dilemma": record["_dilemma"], "ts": str(record.get("timestamp", ""))[:19],
        "elapsed": float(record.get("elapsed") or 0.0),
        "steps": len(steps), "aux": aux, "exec_calls": len(steps) + aux,
        "up": round(up), "lo": round(lo), "out": round(out),
        "mcalls": mcalls, "pv": pv_scores, "rel": rel_calls, "align": align_calls,
        "min_": round(min_), "mout": round(mout),
        "kb": kb, "rb": rb, "esc": len(steps),
        "princ": n_principles, "filt": n_filtered,
    }


#
===========================================================================
=============
# WHOLE-ARCHIVE DIAGNOSTICS   (all stored records, retries included)
#
===========================================================================
=============
```

```python
def truncation_table(records: list[dict]) -> pd.DataFrame:
    """Step-output length against the 400-character bundle limit."""
    rows = []
    for tag, _, label in ARCHITECTURES:
        lengths = []
        for rec in (r for r in records if r["_arch"] == tag):
            cr = rec.get("crem_result") or {}
            lengths += [len(str(cr.get(f"A{s}") or "")) for s in executed_steps(rec)]
        if not lengths:
            continue
        arr = np.array(lengths, dtype=float)
        retained = np.minimum(arr, BUNDLE_TRUNCATION) / np.maximum(arr, 1)
        rows.append({
            "Condition": label,
            "Executed steps (all stored records)": len(arr),
            "Mean characters per step": int(round(arr.mean())),
            "Median": int(np.median(arr)),
            "Steps exceeding 400 characters": f"{100 * (arr > BUNDLE_TRUNCATION).mean():.1f}%",
            "Mean retained proportion": f"{100 * retained.mean():.1f}%",
        })
    return pd.DataFrame(rows)


def forensics_table(records: list[dict]) -> pd.DataFrame:
    """Signatures of the two procedural-validity scoring regimes."""
    acc = defaultdict(lambda: dict(n=0, chars=[], empty=0, named=0, flag=0))
    label_pat = re.compile(r"₩b(step₩s*₩d{1,2}|A₩d{1,2})₩b", re.I)
    for rec in records:
        tag = rec["_arch"]
        per_step = ((rec.get("evaluation_scores") or {}).get("procedural_validity") or {}).get("per_step") or {}
        for node in per_step.values():
            if isinstance(node, dict) and "executed" in node:
                acc[tag]["flag"] += 1
            for entry in evaluator_entries(node):
                reason = str(entry.get("reason") or "")
                acc[tag]["n"] += 1
                acc[tag]["chars"].append(len(reason))
                if not reason.strip():
```

```
                    acc[tag]["empty"] += 1
                if label_pat.search(reason):
                    acc[tag]["named"] += 1
    rows = []
    for tag, _, label in ARCHITECTURES:
        d = acc.get(tag)
        if not d or d["n"] == 0:
            continue
        arr = np.array(d["chars"], dtype=float)
        rows.append({
            "Indicator": label,
            "Reasons analysed": d["n"],
            "Mean reason length (characters)": round(arr.mean(), 1),
            "Median length": int(np.median(arr)),
            "Empty after parsing": f"{100 * d['empty'] / d['n']:.1f}%",
            "Reasons naming a step": f"{100 * d['named'] / d['n']:.1f}%",
            "Per-step 'executed' flag": "present" if d["flag"] else "absent",
        })
    return pd.DataFrame(rows)


#
================================================================
=============
# WORKBOOK
#
================================================================
=============

AC, HB, SB = "1F4E79", "D9E2F3", "EDF1F9"
F_T = Font(name="Arial", size=12, bold=True, color=AC)
F_H = Font(name="Arial", size=10, bold=True)
F_B = Font(name="Arial", size=10)
F_N = Font(name="Arial", size=9, italic=True, color="666666")
F_I = Font(name="Arial", size=10, color="0000FF")
FILL_H = PatternFill("solid", fgColor=HB)
FILL_S = PatternFill("solid", fgColor=SB)
FILL_Y = PatternFill("solid", fgColor="FFFF00")
_TH = Side(style="thin", color="BFBFBF")
```

```python
BD = Border(left=_TH, right=_TH, top=_TH, bottom=_TH)
CEN = Alignment(horizontal="center", vertical="center", wrap_text=True)
LEF = Alignment(horizontal="left", vertical="center", wrap_text=True)


def _header(ws, row, values):
    for j, v in enumerate(values, 1):
        c = ws.cell(row, j, v)
        c.font, c.fill, c.border, c.alignment = F_H, FILL_H, BD, CEN


def _row(ws, row, values, left_cols=()):
    for j, v in enumerate(values, 1):
        c = ws.cell(row, j, v)
        c.font, c.border = F_B, BD
        c.alignment = LEF if (j - 1) in left_cols else CEN


def _note(ws, row, ncols, text, height=70):
    ws.merge_cells(start_row=row, start_column=1, end_row=row, end_column=ncols)
    c = ws.cell(row, 1, text)
    c.font, c.alignment = F_N, LEF
    ws.row_dimensions[row].height = height


def _widths(ws, widths):
    for j, w in enumerate(widths, 1):
        ws.column_dimensions[get_column_letter(j)].width = w


def build_workbook(df: pd.DataFrame, records: list[dict], out_path: Path) -> None:
    wb = openpyxl.Workbook()
    wb.remove(wb.active)

    # ---------------- Unit_Prices ----------------
    ws = wb.create_sheet("Unit_Prices")
    ws.cell(1, 1, "Unit price assumptions (USD per 1M tokens) — rates in effect during the "
                  "experiment period, 2026-04-20 to 2026-05-30").font = F_T
    ws.cell(2, 1, "Prices are those applicable on the execution dates recorded in the logs, "
```

```
                   "not prices at the time of writing.").font = F_N
_header(ws, 4, ["Executor LLM", "Model", "Input $/1M", "Output $/1M",
                    "Price basis and source (as of the execution dates)"])
for i, name in enumerate(LLM_ORDER):
    r = 5 + i
    p = PRICES[name]
    _row(ws, r, [name, p["model"], p["inp"], p["out"], p["src"]], left_cols=(0, 1, 4))
    for j in (3, 4):
        c = ws.cell(r, j)
        c.font, c.fill, c.number_format = F_I, FILL_Y, "0.00"
_note(ws, 11, 5,
      "Legend: the yellow cells are editable inputs; every cost figure in this workbook "
      "recalculates from these five rows. Values are standard on-demand list prices using "
      "uncached / cache-miss input, and exclude batch discounts, prompt caching, tool and "
      "search charges, taxes, and reseller markups.")
_widths(ws, [14, 20, 12, 12, 95])
price_row = {name: 5 + i for i, name in enumerate(LLM_ORDER)}

# ---------------- by-LLM sheets ----------------
meas_total_row = {}
for tag, sheet, label in ARCHITECTURES:
    sub = df[df["arch"] == tag]
    if sub.empty:
        continue
    ws = wb.create_sheet(sheet)
    ws.cell(1, 1, f"{label}: executor-side calls, tokens, latency, estimated cost").font = F_T
    _header(ws, 3, ["Executor LLM", "Model", "Runs", "Mean steps /run",
                    "Mean auxiliary calls /run", "Mean executor calls /run",
                    "Total executor calls", "Mean input tok /run", "Mean output tok /run",
                    "Total input tok (upper)", "Total input tok (lower)",
                    "Total output tok", "Executor cost ($)", "Mean latency (s)"])
    r = 3
    for name in LLM_ORDER:
        s = sub[sub["ExLLM"] == name]
        if s.empty:
            continue
        r += 1
        pr = price_row[name]
        _row(ws, r, [name, PRICES[name]["model"], len(s),
```

```
                              round(s["steps"].mean(), 2), round(s["aux"].mean(), 2),
                              round(s["exec_calls"].mean(), 2), int(s["exec_calls"].sum()),
                              int(s["up"].mean()), int(s["out"].mean()),
                              int(s["up"].sum()), int(s["lo"].sum()), int(s["out"].sum()),
                              f"=J{r}/1000000*Unit_Prices!C{pr}+L{r}/1000000*Unit_Prices!D{pr}",
                              round(s["elapsed"].mean(), 1)], left_cols=(0, 1))
            ws.cell(r, 13).number_format = "$#,##0.00"
        total = r + 1
        _row(ws, total, [f"Total ({label})", "",
                              f"=SUM(C4:C{r})", f"=AVERAGE(D4:D{r})", f"=AVERAGE(E4:E{r})",
                              f"=G{total}/C{total}", f"=SUM(G4:G{r})",
                              f"=J{total}/C{total}", f"=L{total}/C{total}",
                              f"=SUM(J4:J{r})", f"=SUM(K4:K{r})", f"=SUM(L4:L{r})",
                              f"=SUM(M4:M{r})", f"=AVERAGE(N4:N{r})"], left_cols=(0,))
        for j in range(1, 15):
            ws.cell(total, j).fill = FILL_S
        ws.cell(total, 13).number_format = "$#,##0.00"
        _note(ws, total + 2, 14,
              "Note. Executor side only. Input tokens (upper) assume the full accumulated "
              "transcript is resent at each step; (lower) assumes a minimal handoff of dilemma "
              "plus own request plus preceding answer. The cost column uses the upper bound. "
              "Auxiliary sub-model calls are counted as |sub_llms| x executed fan-out steps.")
        _widths(ws, [14, 20, 8, 12, 14, 14, 13, 13, 13, 15, 15, 13, 13, 12])
        meas_total_row[tag] = None   # filled in by the Measurement_Cost sheet below

    # ---------------- Measurement_Cost ----------------
    ws = wb.create_sheet("Measurement_Cost")
    ws.cell(1, 1, "Evaluator-side (measurement) call breakdown and cost — all four conditions").font
= F_T
    _header(ws, 2, ["Condition / component", "Calls /run (mean)", "Calls (all runs)",
                    "Share within condition", "Input tok", "Output tok", "Cost ($)"])
    r = 2
    for tag, _, label in ARCHITECTURES:
        s = df[df["arch"] == tag]
        if s.empty:
            continue
        bundled = tag == "Single"
        pv_label = ("Procedural validity (1 bundled 20-step prompt x 5 evaluators)" if bundled
                    else "Procedural validity (1 prompt per executed step x 5 evaluators)")
```

```
        pv_run = N_EVALUATORS if bundled else s["pv"].mean()
        components = [
            ("Consistency (1 bundled prompt x 5 evaluators)", N_EVALUATORS),
            ("Justifiability (1 bundled prompt x 5 evaluators)", N_EVALUATORS),
            (pv_label, pv_run),
            ("NAI relevance (1 prompt per principle x 5 evaluators)", s["rel"].mean()),
            ("NAI alignment (1 prompt per filtered principle x 5 evaluators)", s["align"].mean()),
        ]
        r += 1
        _row(ws, r, [label, "", "", "", "", "", ""], left_cols=(0,))
        for j in range(1, 8):
            ws.cell(r, j).fill = FILL_S
        first = r + 1
        for name, per_run in components:
            r += 1
            _row(ws, r, [name, round(per_run, 1), int(round(per_run * len(s))), "", "", "", ""],
                 left_cols=(0,))
        r += 1
        total_row = r
        prices_in = "SUMPRODUCT(Unit_Prices!C5:C9)/5"
        prices_out = "SUMPRODUCT(Unit_Prices!D5:D9)/5"
        _row(ws, total_row, [f"Total — {label}", round(s["mcalls"].mean(), 1),
                             int(s["mcalls"].sum()), 1.0,
                             int(s["min_"].sum()), int(s["mout"].sum()),

f"={prices_in}*E{total_row}/1000000+{prices_out}*F{total_row}/1000000"],
             left_cols=(0,))
        for j in range(1, 8):
            ws.cell(total_row, j).fill = FILL_S
        ws.cell(total_row, 7).number_format = "$#,##0.00"
        for rr in range(first, total_row):
            ws.cell(rr, 4, f"=C{rr}/$C${total_row}").number_format = "0.0%"
        ws.cell(total_row, 4).number_format = "0.0%"
        meas_total_row[tag] = total_row
    _note(ws, r + 2, 7,
          "Note. Five evaluator models are queried for every component in every condition. "
          "Procedural validity is the sole asymmetry: the single-agent condition places all "
          "twenty step slots in one prompt, whereas the multi-agent family issues one prompt "
          "per executed step. Measurement cost is charged at the mean of the five evaluator "
```

```
        "prices, since each evaluator receives the same prompt.")
_widths(ws, [58, 16, 14, 16, 14, 14, 12])

# ---------------- T1_Condition_Summary ----------------
ws = wb.create_sheet("T1_Condition_Summary", 1)
ws.cell(1, 1, "Table. Model calls, tokens, latency, and estimated cost by architectural "
              "condition (measured from the reasonbank JSON records)").font = F_T
heads = ["Condition", "Runs", "Execution dates", "Executor calls /run",
         "Measurement calls /run", "Total calls /run", "Total calls",
         "Executor input tok", "Executor output tok", "Measurement input tok",
         "Measurement output tok", "Total tokens", "Mean latency (s)", "SD",
         "Median (s)", "vs Single", "Executor cost ($)", "Measurement cost ($)",
         "Total cost ($)"]
_header(ws, 3, heads)
base = df.loc[df["arch"] == "Single", "elapsed"].mean()
r = 3
for tag, sheet, label in ARCHITECTURES:
    s = df[df["arch"] == tag]
    if s.empty:
        continue
    r += 1
    n_llm = s["ExLLM"].nunique()
    exec_ref = f"='{sheet}'!M{4 + n_llm}"
    meas_ref = f"=Measurement_Cost!G{meas_total_row[tag]}"
    dates = f"{s['date'].min()} ~ {s['date'].max()}"
    _row(ws, r, [label, len(s), dates,
                 round(s["exec_calls"].mean(), 2), round(s["mcalls"].mean(), 2),
                 round((s["exec_calls"] + s["mcalls"]).mean(), 2),
                 int((s["exec_calls"] + s["mcalls"]).sum()),
                 int(s["up"].sum()), int(s["out"].sum()),
                 int(s["min_"].sum()), int(s["mout"].sum()),
                 int(s["up"].sum() + s["out"].sum() + s["min_"].sum() + s["mout"].sum()),
                 round(s["elapsed"].mean(), 1), round(s["elapsed"].std(ddof=1), 1),
                 round(s["elapsed"].median(), 1),
                 f"{s['elapsed'].mean() / base:.2f}x" if base else "",
                 exec_ref, meas_ref, f"=Q{r}+R{r}"], left_cols=(0, 2))
    for j in (8, 9, 10, 11, 12):
        ws.cell(r, j).number_format = "#,##0"
    for j in (13, 14, 15):
```

```
            ws.cell(r, j).number_format = "0.0"
        for j in (17, 18, 19):
            ws.cell(r, j).number_format = "$#,##0.00"
_note(ws, r + 2, len(heads),
      "Note. N = 500 matched execution units per condition, recovered from the stored "
      "reasonbank records; duplicate retries were removed by keeping the latest run per "
      "dilemma x executor x round. Executor calls = executed CREM steps plus, in the "
      "multi-LLM conditions, auxiliary sub-model calls (|sub_llms| x fan-out steps). "
      "Measurement calls = evaluator-LLM calls: 5 consistency, 5 justifiability, "
      "5 per principle for NAI relevance, 5 per filtered principle for NAI alignment, and "
      "procedural validity at 5 per execution in the single-agent condition against "
      "5 x executed steps in the multi-agent family. Token counts use the heuristic "
      "estimator max(words x 1.3, characters / 4) because provider tokenizers were "
      "unavailable offline (approximately +/-15%). Costs recalculate from the Unit_Prices "
      "sheet at the rates in effect on the execution dates.")
_widths(ws, [16, 8, 22] + [14] * 9 + [12, 10, 12, 10, 14, 15, 13])

# ---------------- Provenance ----------------
ws = wb.create_sheet("Provenance")
ws.cell(1, 1, "Data provenance, measurement rules, and known caveats").font = F_T
_header(ws, 2, ["Item", "Detail"])
n_files = len(records)
counts = ", ".join(f"{TAG_LABEL[t]} {sum(1 for x in records if x['_arch'] == t)}"
                   for t, _, _ in ARCHITECTURES)
items = [
    ("Source records",
     f"{n_files} reasonbank JSON files ({counts}). Duplicate retries removed by keeping the "
     f"latest timestamp per dilemma x executor LLM x round, leaving {len(df)} execution units."),
    ("Condition assignment",
     "Read from the records themselves: agent_mode = 'single' gives the single-agent "
     "condition; among multi-agent records, use_rb = True gives Multi-LLM+KRB and a "
     "non-empty sub_llms list gives Multi-LLM. Folder names are not used."),
    ("Executor calls",
     "Executed CREM steps (one model call each) plus |sub_llms| x executed fan-out steps "
     f"in the multi-LLM conditions; fan-out steps are {', '.join(map(str, FANOUT_STEPS))}."),
    ("Measurement calls",
     "5 consistency + 5 justifiability + procedural validity (5 bundled in the single-agent "
     "condition, 5 x executed steps in the multi-agent family) + 5 x principles + "
     "5 x filtered principles."),
```

```
        ("Tokens",
         "Heuristic estimator max(words x 1.3, characters / 4); provider tokenizers were "
         "unavailable offline, so expect approximately +/-15%. Auxiliary sub-model outputs are "
         "embedded in the synthesis text and are not separately recoverable."),
        ("Measurement input tokens",
         "Reconstructed: the records preserve every evaluator response but not the prompt that "
         f"produced it. Constants BUNDLE_INSTRUCTION_TOKENS = {BUNDLE_INSTRUCTION_TOKENS} and "
         f"NAI_CONTEXT_TOKENS = {NAI_CONTEXT_TOKENS} were calibrated against the reference "
         "workbook; mean absolute error 0.22% per run."),
        ("Measurement output tokens",
         "Read directly from the stored evaluator reason text; procedural-validity slots "
         "recorded as 'not executed' are excluded. Mean absolute error against the reference "
         "workbook 0.02% per run."),
        ("400-character truncation",
         "The bundled procedural-validity prompt inserts each step as str(content)[:400]. "
         "Retained proportions by condition are given on the Truncation sheet."),
        ("Self-evaluation",
         "The measurement stage enables the full five-model registry irrespective of the "
         "executing model, so every execution is scored by an ensemble that includes its own "
         "executor."),
        ("Prices",
         "Rates in effect on the execution dates (2026-04-20 to 2026-05-30). From 2026-05-15 "
         "the grok-3 slug was billed as Grok-4.3; nine of the 400 Grok executions post-date "
         "that change."),
        ("Excluded from cost",
         "Batch discounts, prompt caching, tool and search charges, taxes, and cloud or "
         "reseller markups."),
        ("Model configuration",
         "gpt-4.1; claude-haiku-4-5-20251001; gemini-2.5-flash with thinking_budget = 0; "
         "grok-3; deepseek-chat. All at temperature 0.0, max_tokens 3072, max_retries 3."),
    ]
    for i, (k, v) in enumerate(items, start=3):
        _row(ws, i, [k, v], left_cols=(0, 1))
    _widths(ws, [26, 130])

    # ---------------- Truncation and Forensics ----------------
    trunc = truncation_table(records)
```

```
    ws = wb.create_sheet("Truncation")
    ws.cell(1, 1, "Step-output length and the 400-character truncation in the bundled prompt").font
= F_T
    ws.cell(2, 1, "The single-agent bundled prompt inserts each step as str(content)[:400]. Step "
                  "outputs are longer in the multi-agent family, so any bundled evaluation of "
                  "those conditions would lose more text.").font = F_N
    _header(ws, 3, list(trunc.columns))
    for i, row in enumerate(trunc.itertuples(index=False), start=4):
        _row(ws, i, list(row), left_cols=(0,))
    _note(ws, 4 + len(trunc) + 1, len(trunc.columns),
          "Note. Computed over all stored records, including superseded retries, because these "
          "figures describe the evaluator's view of the material rather than the experiment.")
    _widths(ws, [18, 28, 22, 12, 26, 22])

    forensic = forensics_table(records)
    ws = wb.create_sheet("Measurement_Forensics")
    ws.cell(1, 1, "Signatures of the two procedural-validity scoring regimes").font = F_T
    ws.cell(2, 1, "Standalone per-step replies are longer than items parsed out of one bundled "
                  "twenty-item reply, never fail extraction, and restate the step label; a "
                  "bundled reply carries the label outside the reason text.").font = F_N
    _header(ws, 3, list(forensic.columns))
    for i, row in enumerate(forensic.itertuples(index=False), start=4):
        _row(ws, i, list(row), left_cols=(0,))
    _widths(ws, [18, 16, 26, 14, 18, 20, 20])

    # ---------------- Per_Run_All ----------------
    ws = wb.create_sheet("Per_Run_All")
    ws.cell(1, 1, f"Per-run measurements, all four conditions ({len(df)} runs after "
                  f"deduplication)").font = F_T
    cols = ["arch", "ExLLM", "round", "dilemma", "ts", "elapsed", "steps", "aux",
            "exec_calls", "up", "lo", "out", "mcalls", "pv", "rel", "align",
            "min_", "mout", "kb", "rb", "esc", "princ", "filt"]
    _header(ws, 2, cols)
    for i, row in enumerate(df[cols].itertuples(index=False), start=3):
        for j, v in enumerate(row, 1):
            c = ws.cell(i, j, v)
            c.font, c.border = F_B, BD
    _widths(ws, [10, 10, 8, 30, 20] + [9] * 18)
```

```
    wb.save(out_path)
    print(f"   workbook written to {out_path}")


#
=====================================================================
=============
# VALIDATION
#
=====================================================================
=============

def validate(df: pd.DataFrame, reference: Path) -> None:
    """Compare the per-run table against a reference workbook, row by row."""
    ref = pd.read_excel(reference, sheet_name="Per_Run_All", skiprows=2)
    ref["_key"] = ref["ts"].astype(str).str[:19]
    cur = df.copy()
    cur["_key"] = cur["ts"].astype(str).str[:19]
    merged = cur.merge(ref, on="_key", suffixes=("", "_ref")).drop_duplicates("_key")
    print(f"₩n   validation: {len(merged)} runs matched by timestamp")
    print(f"   {'column':<12}{'exact':>8}{'mean |err|':>13}{'max |err|':>12}")
    for col in ["elapsed", "steps", "aux", "exec_calls", "up", "lo", "out",
                "mcalls", "pv", "rel", "align", "min_", "mout", "princ", "filt"]:
        if f"{col}_ref" not in merged:
            continue
        a = merged[col].astype(float)
        b = merged[f"{col}_ref"].astype(float)
        exact = 100 * (a == b).mean()
        rel = ((a - b).abs() / b.replace(0, np.nan)).dropna()
        print(f"   {col:<12}{exact:>7.1f}%{100 * rel.mean():>12.3f}%{100 * rel.max():>11.3f}%")


#
=====================================================================
=============
# MAIN
#
=====================================================================
=============
```

```
def main() -> int:
    ap = argparse.ArgumentParser(description="Regenerate Table S3 from the execution records.")
    ap.add_argument("--root", required=True, type=Path,
                    help="directory containing the reasonbank JSON archives")
    ap.add_argument("--out", type=Path,
                    default=Path("Table_S3_calls_tokens_latency_cost.xlsx"))
    ap.add_argument("--validate", type=Path, default=None,
                    help="optional reference workbook to check the per-run table against")
    args = ap.parse_args()

    print(f"Reading execution records from {args.root} ...")
    records = load_records(args.root)
    if not records:
        print("No reasonbank records found.", file=sys.stderr)
        return 1
    print(f"   loaded {len(records)} files")
    kept = deduplicate(records)

    df = pd.DataFrame(measure_record(r) for r in kept)
    df["date"] = pd.to_datetime(df["ts"], errors="coerce").dt.date
    df["arch"] = pd.Categorical(df["arch"], [t for t, _, _ in ARCHITECTURES], ordered=True)
    df = df.sort_values(["arch", "ExLLM", "round", "dilemma"]).reset_index(drop=True)

    build_workbook(df, records, args.out)
    if args.validate:
        validate(df, args.validate)
    return 0


if __name__ == "__main__":
    raise SystemExit(main())
```
